# De novo methylation of histone H3K23 by the methyltransferases EHMT1/GLP and EHMT2/G9a

**DOI:** 10.1186/s13072-022-00468-1

**Published:** 2022-11-21

**Authors:** David A. Vinson, Kimberly E. Stephens, Robert N. O’Meally, Shri Bhat, Blair C. R. Dancy, Robert N. Cole, Srinivasan Yegnasubramanian, Sean D. Taverna

**Affiliations:** 1 Department of Pharmacology and Molecular Sciences, Johns Hopkins University School of Medicine, Baltimore, MD 21205 USA; 2 Center for Epigenetics, Johns Hopkins University School of Medicine, Baltimore, MD 21205 USA; 3 Department of Pediatrics, Division of Infectious Diseases, University of Arkansas for Medical Sciences, Arkansas Children’s Research Institute, Little Rock, AR 72202 USA; 4 Department of Biological Chemistry, Johns Hopkins University School of Medicine, Baltimore, MD 21205 USA; 5 Sidney Kimmel Comprehensive Cancer Center, Johns Hopkins University School of Medicine, Baltimore, MD 21205 USA; 6 Walter Reed Army Institute of Research, Silver Spring, MD 20910-7500 USA

**Keywords:** Histone, H3K23 methylation, H3K18 methylation, G9a, EHMT2, GLP, EHMT1, Epigenetics

## Abstract

Epigenetic modifications to histone proteins serve an important role in regulating permissive and repressive chromatin states, but despite the identification of many histone PTMs and their perceived role, the epigenetic writers responsible for generating these chromatin signatures are not fully characterized. Here, we report that the canonical histone H3K9 methyltransferases EHMT1/GLP and EHMT2/G9a are capable of catalyzing methylation of histone H3 lysine 23 (H3K23). Our data show that while both enzymes can mono- and di-methylate H3K23, only EHMT1/GLP can tri-methylate H3K23. We also show that pharmacologic inhibition or genetic ablation of EHMT1/GLP and/or EHMT2/G9a leads to decreased H3K23 methylation in mammalian cells. Taken together, this work identifies H3K23 as a new direct methylation target of EHMT1/GLP and EHMT2/G9a, and highlights the differential activity of these enzymes on H3K23 as a substrate.

**Supplementary Information:**

The online version contains supplementary material available at 10.1186/s13072-022-00468-1.

## Background

Post-translational modifications (PTMs) on lysines of histone proteins have long been implicated in regulating gene expression, DNA replication, DNA damage repair, transposon silencing and other template-dependent processes [1–7]. Histone lysine methylation is added by histone methyltransferases (HMTs) that catalyze the addition of one (mono-), two (di-) or three (tri-) methyl groups (–CH_3_) onto a single lysine [5, 7–9]. These different modification states can often facilitate differential binding of effector proteins, which ‘read’ the various modification states and help direct additional chromatin modifying complexes to specific genomic regions and help drive locus-specific chromatin remodeling events [10–15]. The HMTs responsible for the addition of methyl groups to histones are sometimes capable of writing more than one modification state, but often must work in concert with other proteins and HMTs to generate all three modification states genome-wide [3, 4, 16–20].

Histone H3, 1 of the 4 core histones, contains several well-characterized lysine targets for modification by methyltransferases within its N-terminal tail, including H3K4, H3K9, H3K27, and H3K36. In eukaryotes where methylation is detected at these positions, some methylation events are linked to gene repression and facilitating a closed chromatin conformation (H3K9me and H3K27me), while methylation at other H3 lysines is connected to active gene transcription and facilitating an open chromatin conformation (H3K4me and H3K36me) [3, 4, 10, 16, 21–24]. Another conserved documented site of histone H3 methylation in organisms ranging from ciliates to mammals is H3K23. Although the biological roles for methylation on this residue are poorly characterized, they have been associated with gene repression and maintaining genome integrity [25–28]. While methyltransferases catalyzing H3K23 methylation have been identified in protists, nematodes, and plants, mammalian writers of these methylation marks have not been reported.

In this study, we report that H3K23 represents a new target of the enzymes euchromatic histone methyltransferase 1 (EHMT1/GLP) and euchromatic histone methyltransferase 2 (EHMT2/G9a). More specifically, we report that while both EHMT1/GLP and EHMT2/G9a enzymes can catalyze the addition of mono- and di-methylation on H3K23, only EHMT1/GLP can catalyze the addition of tri-methylation on H3K23 in vitro. Additionally, we show that perturbations in EHMT1/GLP and/or EHMT2/G9a at either the protein or gene level leads to decreased methylation on H3K23 in vivo. Interestingly, our in vitro and in vivo studies also revealed H3K18 as a new target for mono-, di- and tri-methylation by both EHMT1/GLP and EHMT2/G9a. Taken together, this work establishes histone H3 lysines 18 and 23 as new methylation targets for EHMT1/GLP and EHMT2/G9a, and identifies their differential activity on H3K23.

## Results

### EHMT1/GLP and EHMT/G9a can de novo methylate histone H3K23 in vitro

**Fig. 1:**
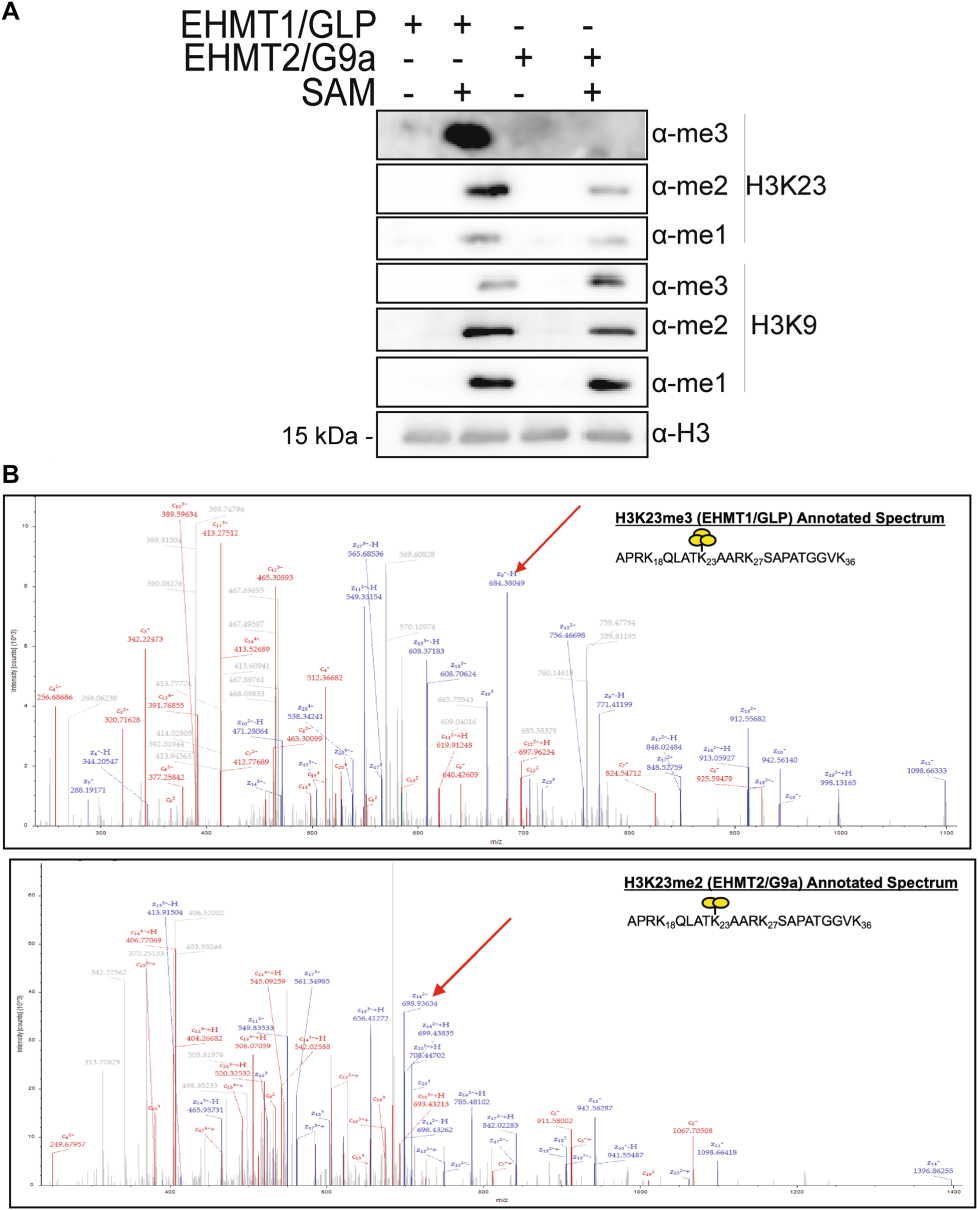
EHMT1/GLP and EHMT2/G9a can de novo methylate histone H3K9 and H3K23 in vitro. **A** Recombinant histone H3.1 was incubated with SAM and either recombinant EHMT1/GLP or EHMT2/G9a for 24 h, and these in vitro HMT reactions were subjected to western blotting to detect the abundance of various H3K9 and H3K23 methylation states. **B** Mass spectrometry was used to validate the presence of specific methylation states catalyzed by EHMT1/GLP or EHMT2/G9a in the in vitro HMT assays. Representative spectra for H3K23me3 in the EHMT1/GLP HMT reactions and H3K23me2 in the EHMT2/G9a reactions are shown in panel B. Please also see Additional file 1: Fig. S1, which contains ions and mass error tables that correspond to this spectra, and contains spectra of additional methylation states detected in the in vitro HMT assays

While H3K23 methylation has been observed in mammalian chromatin, mammalian HMTs that catalyze H3K23 methylation have not been reported. However, in other species such as *A. thaliana*, *C. elegans* and *T. thermophila*, histone methyltransferases that modify or are similar to those that modify H3K9 and H3K27 have been shown to also target H3K23 [25–28]. To screen which mammalian HMTs act on histone H3K23, we employed an in vitro histone methyltransferase assay. We chose to screen potential H3K23 methyltransferase activity of six known mammalian H3K9 methyltransferases, Suv39h1, Suv39h2, SetDB1, SetDB2, EHMT1/GLP, and EHMT2/G9a. As previously reported, [4, 5, 7, 8], recombinant Suv39h1, Suv39h2, SetDB1, SetDB2, EHMT1/GLP, and EHMT2/G9a, were all able to de novo methylate H3K9 to different extents (data not shown). Of the HMTs screened, only EHMT1/GLP and EHMT2/G9a showed activity on H3K23. More specifically, our data show that while both EHMT1/GLP and EHMT2/G9a can mono- and di-methylate H3K23, only EHMT1/GLP can tri-methylate H3K23 as evaluated both by western blotting using histone modification specific antibodies for H3K9 and H3K23 (Fig. 1A) and electron transfer dissociation (EthD) mass spectrometry (Fig. 1B). To validate that EHMT1/GLP and EHMT2/G9a were not indiscriminately targeting lysines on H3, we tested for two additional well described histone H3 tri-methyl marks by western blotting: H3K4me3 and H3K36me3. Our data show that neither H3K4me3 nor H3K36me3 are generated in our in vitro HMT system by EHMT1/GLP or EHMT2/G9a, suggesting that these enzymes are indeed discriminating between targets and methylation states (Additional file 1: Fig. S1B). In our western blotting and mass spectrometry analysis, we also observed H3K27 and H3K18 methylation to various extents (Additional file 1: Fig. S1A–B). While the ability of EHMT1/GLP and EHMT2/G9a to catalyze methylation at H3K27 has been previously reported [36], we could not find examples in the literature of either of these two enzymes targeting H3K18. A summary of methylation sites targeted by EHMT1/GLP and EHMT2/G9a as detected by our combined western blotting and mass spectrometry data can be found in Table 1. Taken together, this data identifies H3K18 and H3K23 as new targets of EHMT1/GLP and EHMT2/G9a.

**Table 1:** Detectable histone H3 methylation states for the in vitro HMT assays with EHMT1/GLP and EHMT2/G9a

EHMT1/GLP			
	Mono-methyl	Di-methyl	Tri-methyl
Lysine 4	X	X	X
Lysine 9	✔	✔	✔
Lysine 14	X	X	X
Lysine 18	✔	✔	✔
Lysine 23	✔	✔	✔
Lysine 27	✔	✔	✔
Lysine 36	X	X	X
EHMT2/G9a			
	Mono-methyl	Di-methyl	Tri-methyl
Lysine 4	X	X	X
Lysine 9	✔	✔	✔
Lysine 14	X	X	X
Lysine 18	✔	✔	✔
Lysine 23	✔	✔	X
Lysine 27	✔	✔	✔

The table shows the individual methylation states, detected by western blot and mass spec., for the in vitro HMT reactions with EHMT1/GLP and EHMT2/G9a

**Table 2:** Sequence and vendor details for synthetic histone peptides

Peptide name	Sequence	Vendor/catalog #	Experiment
H3K9me1	ARTKQTARK(me1)STGGKAPRKQLK-biotin	EpiCypher; 12–0010	ELISA/antibody validation
H3K9me2	ARTKQTARK(me2)STGGKAPRKQLK-biotin	Active Motif; 81046	ELISA/antibody validation
H3K9me3	ARTKQTARK(me3)STGGKAPRKQLK-biotin	Active Motif; 81047	ELISA/antibody validation
H3K18me1	ARTKQTARKSTGGKAPRK(me1)QLK-biotin	Epicypher; 12–0013	ELISA/antibody validation
H3K18me2	ARTKQTARKSTGGKAPRK(me2)QLK-biotin	Epicypher; 12–0014	ELISA/antibody validation
H3K18me3	ARTKQTARKSTGGKAPRK(me3)QLK-biotin	Epicypher; 12–0015	ELISA/antibody validation
H3K23me1	KQLATK(me1)AARKSAPATGGVYK-biotin	Custom made/Biomatik	ELISA/antibody validation
H3K23me2	KQLATK(me2)AARKSAPATGGVYK-biotin	Custom made/Biomatik	ELISA/antibody validation
H3K23me3	KQLATK(me3)AARKSAPATGGVYK-biotin	Custom made/Biomatik	ELISA/antibody validation
H3K27me1	ATKAARK(me1)SAPSTGGVKKPHRYRPGGGK-biotin	Active Motif; 81050	ELISA/antibody validation
H3K27me2	ATKAARK(me2)SAPSTGGVKKPHRYRPGGGK-biotin	Active Motif; 81051	ELISA/antibody validation
H3K27me3	ATKAARK(me3)SAPSTGGVKKPHRYRPGGGK-biotin	Active Motif; 81052	ELISA/antibody validation
H3K9M	ARTKQTARMSTGGKAPRKQLK-biotin	Made in house	Peptide pulldown
H3K23M	KQLASMAARKSAPATGGIK-biotin	Made in house	Peptide pulldown
H3 unmodified (1–25)	ARTKQTARKSTGGKAPRKQLATKAA-biotin	Epicypher; 12–0107	ELISA, peptide pulldown
H3 unmodified (18–36)	KQLATKAARKSAPATGGVYK-biotin	Custom made/Biomatik	ELISA, peptide pulldown

The table details the sequence, vendor/catalog number and designates the experiment for which the peptides were used

### Pharmacologic inhibition of EHMT1/GLP and EHMT2/G9a by UNC0642 inhibits production of H3K23 methylation in vitro

**Fig. 2:**
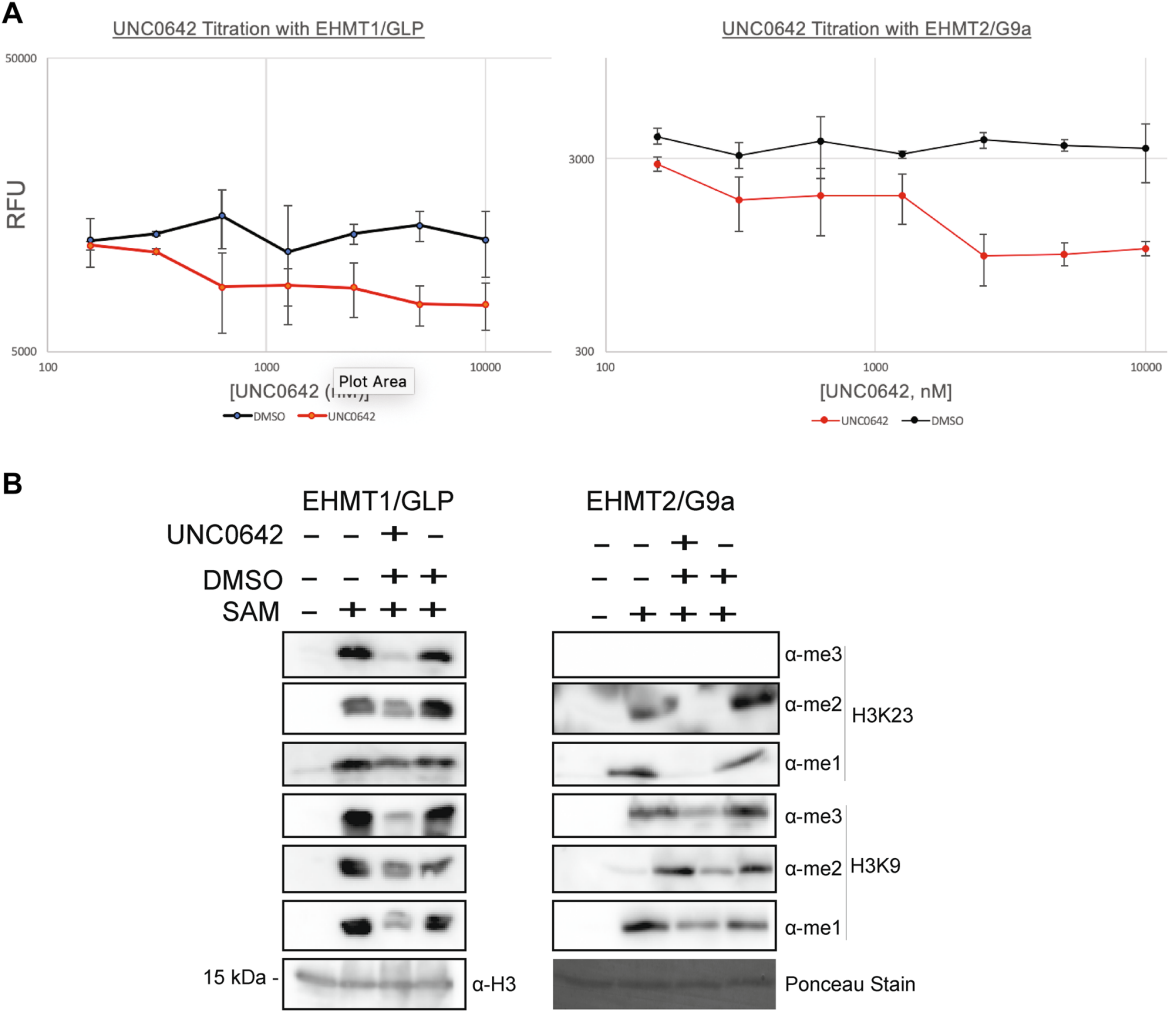
Pharmacologic inhibition of EHMT1/GLP and EHMT2/GLP decreases H3K9 and H3K23 methylation in vitro. **A** A dose titration was performed using the competitive inhibitor UNC0642 with either EHMT1/GLP or EHMT2/G9a, and the enzyme activity in the presence of the inhibitor or DMSO (the vehicle) was measured using the MTase-Glo assay. **B** Recombinant histone H3.1 was incubated with either EHMT1/GLP or EHMT2/G9a, SAM, and either DMSO or 10 μM UNC0642 for 24 h, and subjected to western blotting to detect loss of various H3K9 and H3K23 methylation states in the in vitro HMT inhibition reactions

To further corroborate the enzymatic activity of EHMT1/GLP and EHMT2/G9a on H3K23, we assessed whether an inhibitor of these enzymes, UNC0642 [29, 30], could reduce levels of H3K23 methylation states in our biochemical assay system. UNC0642 is a competitive inhibitor which binds the active site of the SET methyltransferase domain, and prevents the enzyme from accommodating a peptide substrate, like recombinant histone H3 [29]. To compare selectivity of the UNC0642 inhibitor among EHMT1/GLP, EHMT2/G9a, and other SET-domain containing H3K9 HMTs, we performed a UNC0642 dose titration against EHMT1/GLP, EHMT2/G9a, SetDB1, and Suv39h2, using the MTase-Glo activity assay. Of the four enzymes tested, only EHMT1/GLP and EHMT2/G9a showed a significant dose-dependent decline in enzyme activity compared to a DMSO control (Fig. 2A and Additional file 1: Fig. S2A). This inhibition is consistent with prior studies showing specificity of UNC0642 to EHMT1/GLP and EHMT2/G9a as compared to other HMTs (PRMT3, SETD7, SETDB1, SETD8) [29]. Using western blot analysis to evaluate the products of our in vitro methyltransferase reaction, we observed that incubation of recombinant EHMT1/GLP or EHMT2/G9a with UNC0642 causes a decrease in both H3K9 and H3K23 methylation (Fig. 2B). Additionally, we observed that UNC0642-treated EHMT1/GLP or EHMT2/G9a reactions decreased production of both H3K18 and H3K27 methylation (Additional file 1: Fig. S2B, and C). This data suggests that specific enzyme activity of EHMT1/GLP and EHMT2/G9a is responsible for methylation at histone H3K9, H3K18, H3K23, and H3K27 in vitro.

### Inhibition of EHMT1/GLP and EHMT2/G9a, by UNC0642, leads to global reduction of H3K23 methylation in mammalian cells

**Fig. 3:**
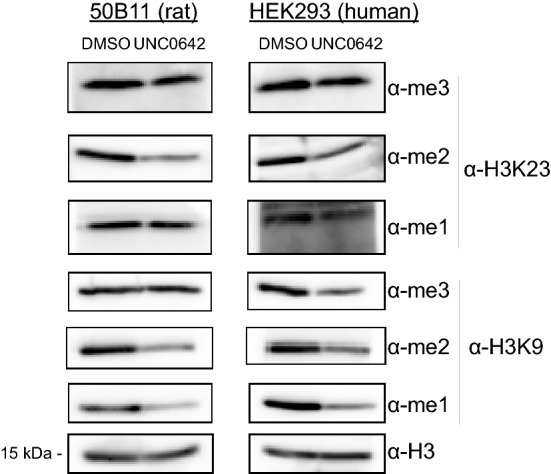
Pharmacologic inhibition of EHMT1/GLP and EHMT2/G9a decreases H3K9 and H3K23 methylation in vivo. 50B11 (rat) or HEK203 (human) cells were treated with either 10 uM UNC0642 or DMSO for 5 days. At the conclusion of the treatment, the cells were harvested, and extracts were blotted for various histone H3K9 or H3K23 methylation states to evaluate changes in these methylation states in vivo

UNC0642 has also been shown to inhibit EHMT1/GLP and EHMT2/G9a methyltransferase activity in vivo [29–31]. We treated various mammalian cell lines with 10 μM UNC0642 for 5 days, after which the cells were harvested, lysed, and blotted for various methylation states of histone H3K9 and H3K23. Consistent with our in vitro studies, our data show that treatment of 50B11 (rat), HEK293 (human), and MC38 (mouse) cell lines with UNC0642 decreased at least one of the methylation states of H3K9 and H3K23 in vivo when compared to a DMSO treated control (Fig. 3 and Additional file 1: Fig. S3A–C). More specifically, mono- and di-methylation were greatly reduced, and tri-methylation was more subtly reduced, if at all, at H3K9 and H3K23. Interestingly, we also observed decreased methylation levels at lysines 18 and, to a lesser extent, 27 (Additional file 1: Fig. S3A–C). Taken together with the known selectivity of the UNC0642 inhibitor, this data implicates the enzymatic activity of EHMT1/GLP and EHMT2/G9a in the catalysis of H3K9, H3K18, H3K23, and H3K27 methylation in vivo.

### Lysine-to-methionine (K-to-M) mutations differentially bind EHMT1/GLP and EHMT2/G9a, and decrease H3K9 and H3K23 methylation levels in vivo

**Fig. 4:**
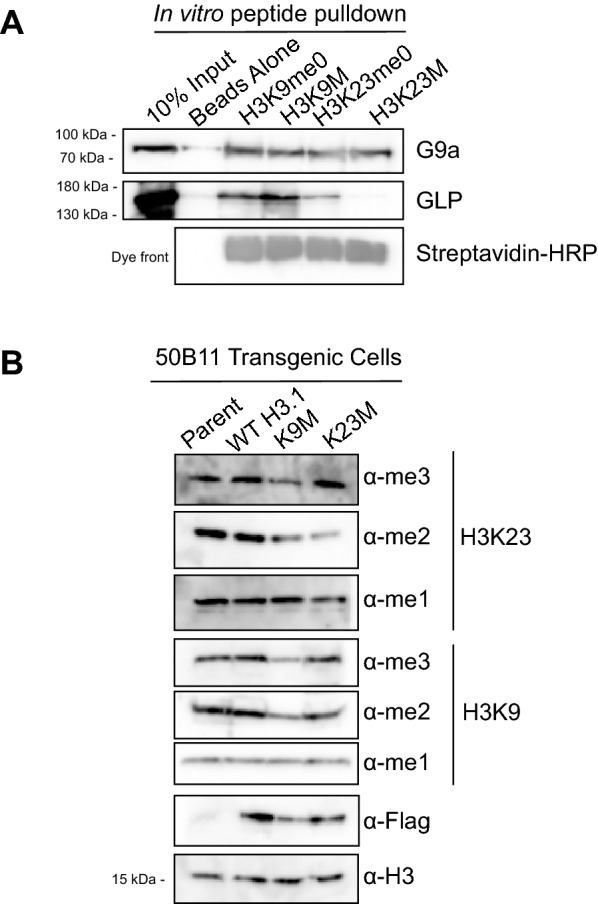
H3K23M differentially interacts with EHMT1/GLP and EHMT2/G9a in vitro and perturbs H3K9 and H3K23 methylation in vivo. **A** Recombinant EHMT1/GLP or EHMT2/G9a were incubated with H3K9me0, H3K9M, H3K23me0, or H3K23M peptides to probe for binding, in vitro, and binding was assessed by western blotting. **B** Histones from 50B11 cells expressing the indicated K-to-M mutations were analyzed using western blotting to determine the effect of these mutations on H3K9 and H3K23 methylation states in vivo

It has been reported that certain lysine-to-methionine (K-to-M) missense mutations in histone proteins can disrupt methylation in a dominant negative fashion at the complementary lysine on native histone [32–34]. While the mechanism of methylation inhibition in K-to-M mutants is not well understood, structural and biochemical work suggests that the methionine mimics a mono-methylated lysine and sequesters the cognate histone methyltransferase (HMT) at the mutated residue, preventing it from methylating lysines on native histone tails [32–34]. We employed this K-to-M approach in an attempt to inhibit H3K23 methylation in vivo, and assessed the ability of these K-to-M mutant histones to bind EHMT1/GLP and EHMT2/G9a in vitro. Accordingly, we employed an in vitro peptide pulldown assay where we separately incubated recombinant EHMT1/GLP or EHMT2/G9a with biotinylated histone peptides representing unmodified H3K9, unmodified H3K23, and the corresponding biotinylated K-to-M mutant peptide (Table 2). Interestingly, our data show that while both enzymes interact with H3K9M peptide as expected [32–34], only recombinant EHMT2/G9a, not EHMT1/GLP, is observed to interact with H3K23M peptide in vitro (Fig. 4A). Intriguingly, our in vitro pulldown data show that while EHMT1/GLP is more enriched in H3K9M vs H3K9me0, EHMT2/G9a seems to be equally enriched in both H3K9me0 and H3K9M, further suggesting these two enzymes differentially interact with histone H3. We next wondered if the H3K9M and H3K23M transgenes decrease H3K23 methylation in vivo, and generated transgenic 50B11 cells expressing wild-type H3.1, H3K9M, or H3K23M mutant histones. Whole cell extracts from these lines were evaluated by western blotting for mono-, di-, and tri-methylation at H3K9 and H3K23. Interestingly, our data show that mono- and di-, but not tri-methylation at H3K23 is perturbed in cells expressing H3K23M. Strikingly, our data also show that only the di- and tri-, but not the mono-, methylation states are perturbed in cells expressing H3K9M mutant histones (Fig. 4B). To ensure that the effects of the H3K9M and H3K23M were not cell type specific, we engineered this set of K-to-M mutants in MC38 cells, and observed a similar ability of H3K9M, but not H3K23M, transgene to perturb H3K23me3 (Additional file 1: Fig. S4A). We did not observe perturbation of H3K23 methylation in K-to-M mutations other than H3K9M or H3K23M (Additional file 1: Fig. S4B). Taken together, our data suggest that EHMT2/G9a, a writer of mono- and di-methylation at H3K23, can bind and be sequestered in vivo by H3K23M, which leads to global perturbations in mono- and di-methylation; whereas EHMT1/GLP, a di- and tri-methyltransferase for lysine 23, is sequestered by H3K9M, leading to global decreases in di- and tri-methylation at H3K23. This data also suggests that, in vivo, EHMT2/G9a may function as the principal mono- and di-methyltransferase for H3K23, while EHMT1/GLP could be the principal tri-methylase for H3K23.

### Deletion of either EHMT1/GLP, EHMT2/G9a, or both genes causes global decreases in H3K23 methylation in mouse embryonic cells

**Fig. 5:**
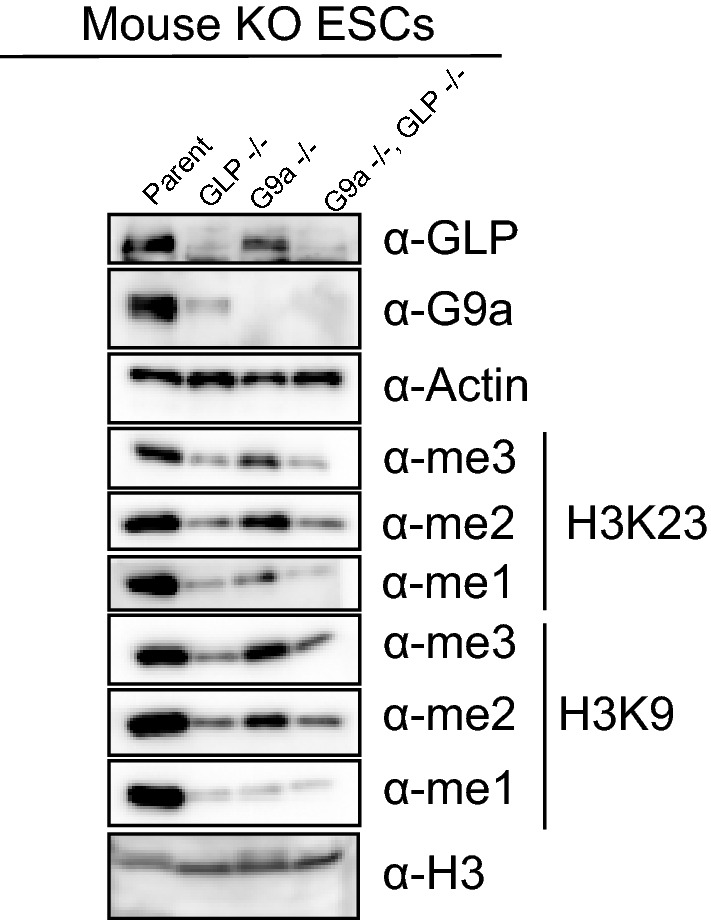
Genetic ablation of GLP, G9a, or both, decreases H3K9 and H3K23 methylation in vivo. Mouse embryonic stem cells were knocked out for GLP, G9a, or both genes, and whole cell extracts were subjected to western blotting with the indicated antibodies to evaluate changes to levels of H3K9 and H3K23 methylation states in vivo

To further validate that EHMT1/GLP and/or EHMT2/G9a contribute to H3K23 methylation levels in vivo, we tested mouse embryonic stem cells in which the genes for either EHMT1/GLP, EHMT2/G9a, or both enzymes were deleted, using western blotting analysis of various methylation states of histone H3K9 and H3K23. Consistent with our inhibition studies, we observe that deletion of either EHMT1/GLP and/or EHMT2/G9A decreases levels of all methylation states of H3K9 and H3K23 (Fig. 5). Interestingly, previous reports [35, 36] have shown that deletion of either EHMT1/GLP and/or EHMT2/G9a perturbs H3K27 methylation levels in vivo, which we were also able to observe (Additional file 1: Fig. S5). Additionally, we observed that deletion of EHMT1/GLP, but not so much EHMT2/G9a, decreased H3K18 methylation levels in vivo (Additional file 1: Fig. S5). Taken together, this data suggests that EHMT1/GLP and/or EHMT2/G9a may indeed contribute to H3K23 and H3K18 methylation levels in mammalian cells, in addition to their well-established roles catalyzing H3K9 methylation, in vivo.

## Conclusions

This study identifies H3K18 and H3K23 as new de novo methylation targets of EHMT1/GLP and EHMT2/G9a. We also show that while both enzymes act on H3K23, they display differential activity in the extent to which they can methylate H3K23; EHMT2/G9a can only mono- and di-methylate H3K23, whereas EHMT1/GLP can mono-, di-, and tri-methylate H3K23 in vitro. We also show, through various perturbations at the gene and protein level in multiple mammalian cell lines, that EHMT1/GLP and EHMT2/G9a contribute to methylation at H3K18 and H3K23 in vivo, suggesting that de novo methylation of these new targets by these enzymes is relevant in vivo and evolutionarily conserved.

## Discussion

Methylation of specific lysine residues on the N-terminal tail of histone H3 helps confer specific chromatin states that regulate access of the underlying DNA sequence to the nuclear environment [3–5, 7, 8, 10, 16, 23, 37]. While many histone lysine methylation sites have been identified, the full complement of enzymes that generate mono-, di-, and tri-methylation at specific residues is not fully known. Here, we identify histone H3K18 and H3K23 as two new targets for the methyltransferases EHMT1/GLP and EHMT2/G9a. We show that while both enzymes can mono-, di-, and tri-methylate H3K18, they display differential activity on H3K23. While both enzymes catalyzed the addition of mono- and di-methylation to H3K23, only EHMT1/GLP catalyzed the tri-methylation of lysine 23 in vitro. We also demonstrate that pharmacological and genetic disruption of EHMT1/GLP and EHMT2/G9a decreased H3K18 and H3K23 methylation in vivo.

More broadly, this work elucidates an expanded repertoire of histone methylation modifications catalyzed by EHMT1/GLP and EHMT2/G9a. While these enzymes were already known to be promiscuous on both non-histone and histone targets [17, 38], this work draws attention to the phenomena that multiple, biologically distinct, histone methylation marks can be catalyzed by the same HMTs in vivo [39, 40]. This work could also shed light on combinatorial post-translational modification states involving these newly identified targets in vivo, as well as provide new insights into mechanisms of PTM cross-talk and how oncohistones can impart changes in the epigenome [41, 42]. While our in vitro HMT system with EHMT1/GLP and EHMT2/G9a revealed the presence of combinatorial H3K18, H3K23, and H3K27 combinatorial methylation states, it remains to be seen if such states exist in cells; but at a minimum our data suggests that the presence of methylation at one target does not impede methylation by EHMT1/GLP or EHMT2/G9a of a neighboring target. Because EHMT1/GLP and EHMT2/G9a exist primarily as a hetero-dimer in vivo rather than individually, as our in vitro system was configured, it also remains unclear what, if any, co-dependence exists for methylation events catalyzed by these enzymes in vivo.

More interestingly, in vivo perturbations in EHMT1/GLP and EHMT2/G9a have been implicated in many cellular processes including autophagy [43], DNA methylation, [35, 44], hypoxia [45], tumor suppression [46–49], chromatin remodeling [20], and synaptic plasticity [50] to name a few, and homozygous loss of EHMT1/GLP results in embryonic lethality in mice [51]. Additionally, both enzymes have been the focus of cancer therapies in recent years [44, 47, 49, 52–54]. While this work does not address the functional role of H3K18 and H3K23 methylation, it is possible that methylation at these targets could contribute to these cellular processes and pathologies. It is also worth noting that H3K23 and H3K9 share a number of chromodomain-containing reader proteins including MPP8, HP1 alpha, HP1 beta, and HP1 gamma [15, 55]. Thus, the extent of the overlap between H3K9, H3K18, and H3K23 interactomes may extend beyond ‘writing’. More extensive studies need to be done to determine the full complement of H3K18 and H3K23 methylation writers, readers, and erasers, and to understand the biological contexts in which such methylation modifications operate.

The EHMT1/GLP and EHMT2/G9a methyltransferases are being pursued as targets for drug development, particularly as potential anti-cancer therapeutics [44, 47, 49, 52–54] including the inhibitor used in this study, UNC0642, as well as multiple additional agents, including a recently described dual inhibitor of EHMT2/G9a and the DNA methyltransferase DNMT1 [44]. These enzymes have been targeted largely due to their well-characterized role in mediating H3K9 methylation, an important heterochromatic mark that is often used by cancer cells to repress large chromatin blocks and repress expression of key tumor suppressor genes and pathways [19, 34, 40, 42]. However, based on the current work, it will be important to assess the impact of such EHMT1/GLP and EHMT2/G9a inhibitors on methylation at other histone lysine methylation modifications including at H3K18, H3K23, and H3K27, since these would represent on-target modifications that, upon EHMT1/GLP or EHMT2/G9a inhibition, may have unanticipated consequences apart from inhibiting H3K9 methylation alone.

Holistically, this work unveils histone H3K18 and H3K23 as new in vivo targets of the methyltransferases EHMT1/GLP and EHMT2/G9a, and demonstrates differential activity between these two enzymes on histone H3K23.

## Methods

### Cell culture methods

50B11 cells were grown and propagated in culture as described previously [56]. Briefly, growth media consisted of NeuroPlex Serum-free Neuronal Medium (Gemini Bioscience, #600-300) and supplemented with Fetal Bovine Serum (10% final, Gemini Biosciences, Cat. 100-106), 100 mM l-glutamate (275 μM final, Gibco, Cat. 25030-081), 20% glucose (0.2% *v*/*v* final), and 10X Gem21 NeuroPlex supplement (1X final, Gemini Biosciences, Cat. 400-160). 50B11 cells were grown in an incubator at 37 ℃, 5% CO_2_ and were passaged no more than 5 times. HEK293 and MC38 cells were grown in 1X DMEM media (Gibco, Cat. 11995-065) containing 10% *v*/*v* FBS (Gemini Biosciences, Cat. 100-106) and 1X Penn/Strep (Gibco, Cat. 10378-016) and were passaged no more than 5 times. Mouse ESCs were grown on TC-treated plates coated with 0.1% *w*/*v* gelatin (Millipore-Sigma, Cat. ES-006-B) with 1X DMEM media (Gibco, Cat. 11995-065) containing 15% *v*/*v* knock-out serum replacement (ThermoFisher, Cat. 10828010), 2-mercaptoethanol (100 μM, Gibco, Cat. 31350-010), Non-essential amino acids (1X, Sigma, Cat. M7145-100 mL), glutamine (2 mM, Gibco, Cat. 25030–081), 1X Penn/Strep as previously described. Mouse ESCs were passaged every 1–2 days to remove differentiated cells and media were changed daily. All cell types were harvested by washing cells 3X in 1X DPBS (Gibco, Cat. 14190-136), trypsinization, and spinning down at 500 rpm at 4 ℃ to pellet cells. All mammalian cell lines were STR profiled and mycoplasma tested for interspecies contamination and mycoplasma bacteria, respectively.

### Generation of transgenic 50B11 cells expressing mutant histones

Plasmids encoding mammalian histone H3.1 with various lysine-to-methionine mutants were constructed using iterative rounds of mutagenic PCR. Once sanger-sequence validated, the resulting H3.1 mutant DNA sequence was subcloned into a lentiviral vector (pLVX-IRES-mCherry). To prepare lentivirus containing the construct encoding the mutant histone, HEK293 cells were plated and grown in 1X DMEM (containing 10% FBS and 1X Penn/Strep) to approximately 70% confluency. To transfect HEK293 cells, Fugene transfection reagent was mixed with 400 μL complete DMEM media and incubated at room temperature for 5 min at a ratio of 3:1 of Fugene to total DNA. To the Fugene/DMEM mixture, 5 μg of the vector encoding mutant H3.1, 3.75 μg of Ä8.9 packaging plasmid and 2.5 μg of VSV-G envelope plasmid was added and allowed to incubate at room temperature for 15 min. The resulting mixture was added to HEK293 cells and incubated at 37 ℃ for 3 h. 90 μL of 1 M sodium butyrate was added (final concentration 110 mg/mL) to open up the chromatin and allow for more efficient integration into the host genome. The following day, the media were discarded and replaced with Opti-MEM, without 1X Penn/Strep, and allowed to incubate for 12–18 h. The virus-enriched supernatant was collected and stored at 4 ℃. This process was repeated 3 times, each time pooling the viral supernatant. Pooled viral supernatant was filtered through a 0.45-μM filter to remove any HEK293 cells carried over. 0.5 mL of viral supernatant was used to transduce 50B11 cells for 24 h. Following transduction, the media were removed and cells allowed to incubate for 2 days. At this point, a small portion of the heterogeneously transduced cells were collected for analysis (Additional file 1: Fig. S4B). The remaining cells were FACS sorted for high vs low mCherry expression into wells in a 384-well plate (1 cell per well). Colonies were expanded, harvested, lysed via resuspension in 2% SDS, and western blotting was performed to evaluate FLAG expression as well as for various methyllysine histone modifications (Fig. 4B).

### Generation of mouse ESCs

Wild-type and knock out mouse ESCs (G9a –/–, GLP –/–, G9a/GLP –/–) were graciously provided by the lab of Dr. Yoichi Shinkai from the Cellular Memory Laboratory at the RIKEN Advanced Science Institute in Japan and generated as previously described [35]. KO cells were validated via western blotting. To generate whole cell lysates for western blotting, cell pellets were resuspended in 2% (w/v) SDS, aggressively vortexed to rupture the cells, and spun down at 16,000 g to clarify lysate from insoluble cellular debris. The resulting protein-rich supernatant was decanted and quantitated using a nanodrop (A280 nm).

### In vitro histone methyltransferase assay

In vitro histone methyltransferase reactions were prepared in buffer containing 20 mM Tris, pH 8.0, 50 mM NaCl, 1 mM EDTA, 3 mM MgCl_2_, 0.1 mg/mL BSA, 1 mM DTT, 20 μM S-adenosyl methionine (Promega, Cat. V7601), 1 μg human recombinant H3.1 (New England Biolabs, Cat. M2503S) and 10 ng of either EHMT1/GLP (Active Motif, Cat. 31920) or G9a/EHMT2 (Active Motif, Cat. 31410) and allowed to incubate for 18–24 h at 25 °C. Reactions were quenched with TFA (0.012% *v*/*v* final concentration). Following quenching, samples were submitted for EthD mass spectrometry or used for western blotting analysis. To prepare samples for western blotting, 1X SDS sample buffer and 1 μL of BME were added to the reactions and the samples were incubated at 98 ^o^C for 5 min, cooled, run on a 16% acrylamide gel, transferred to a PVDF membrane, and blotted for various histone H3 methyl-lysine marks (blotting conditions below).

### In vitro histone methyltransferase inhibition assay

In vitro histone methyltransferase reactions were prepared in buffer containing 20 mM Tris, pH 8.0, 50 mM NaCl, 1 mM EDTA, 3 mM MgCl_2_, 0.1 mg/mL BSA, 1 mM DTT, and 10 μM UNC0642 (or the corresponding amount of DMSO as a control), 20 uM S-adenosyl methionine, 1 μg human recombinant H3.1 and 10 ng of either EHMT1/GLP or EHMT2/G9a. These reactions were allowed to incubate for 18–24 h at 25 ^o^C. Reactions were quenched with TFA (0.012% v/v final concentration). 1X SDS sample buffer and 1 uL of BME was added to the reactions and the samples were subjected to SDS-PAGE separation (run on a 16% gel) followed by western blotting against the histone modifications of interest (see blotting conditions). To assess enzymatic turnover of SAM to SAH by the HMTs, the MTase-Glo kit (Promega, V7601) was used.

### In vivo GLP/G9a inhibition with UNC0642

The corresponding mammalian cell line was cultured in the appropriate media containing 10 μM UNC0642 inhibitor (Sigma Aldrich HY-13980) or the corresponding volume of DMSO as a control for 5 days (media changed daily). All mammalian cell lines were grown at 37 ℃, 5% CO_2_. Upon completion of treatment, whole cell lysates were generated by resuspending cell pellets in 2% SDS, aggressively vortexed to rupture cells, spun down at 16,000 g to clarify from insoluble cellular debris, and decant off the protein-rich supernatant, which was quantitated via a nanodrop (A280 nm).

### Mass spectrometry sample preparation

Each 5 μg of recombinant histone was first resuspended in 50 μl of 20 mM ammonium bicarbonate pH 8.5. Samples were then reduced by adding 5 μL of 7.5 mg/ml (DTT) and put on a heat block at 60 ^o^C for 1 h. After cooling to room temperature samples were then alkylated with 5 μl of 18.5 mg/ml iodoacetamide for 15 min at room temperature in the dark before adding 0.5 μg of Lysyl Endopeptidase MS Grade (Wako/Fuji Osaka, Japan) to make a protein/protease ratio of 10:1. Samples were digested overnight at 37 ℃ before adding 10 ul of 10% *v*/*v* TFA and evaporated to dryness in a speed vac and stored at – 80 ℃. Prior to analysis, samples were resuspended in 100 μl of 10 mM TEAB and subjected to SPE cleanup using stage tips constructed with styrenedivinylbenzene disks (Empore SDB-XC, 3 M Corp.) under basic conditions. This was found to be necessary for retention due to the extremely hydrophilic nature of the modified peptides. Stage tips were wetted with 20 μl 100% acetonitrile followed by preparation with two 20-μl aliquots of 10 mM TEAB followed by loading of 20 μl of the resuspended solution or 1ug of LysC digested protein. This was then eluted with a solution of 10 mM TEAB in 75% *v*/*v* acetonitrile and evaporated to dryness.

### Mass spectrometry analysis

Due to significant loss of these early eluting peptides, a direct on column approach was taken for the stage tipped samples. The entire 1 μg aliquot was resuspended in 5 μl of 2% *v*/*v* acetonitrile, 0.1% formic acid and loaded onto the nanoLC column (75 μm x 20 cm in house packed with ReproSil-Pur C18-AQ, 3 μm Dr. Maisch, Ammerbuch, Germany) at 500 nl/min using an EasyLC chromatography system (Thermo Scientific). Once the entire volume was loaded onto the column a shallow gradient was started at 300 nl/min into an Orbitrap Fusion Lumos mass spectrometer (Thermo Scientific) equipped with electron transfer dissociation (ETD) capability. The samples were run in HCD mode at first, but none of the methylated peptides were positive hits when searched. Upon switching to EThcD, several of the isoforms of methylated peptides were found to exist in the +4, +5, and +6 charge states eluting early in the run before the bulk of the unmodified peptides. Since the methylation of the lysines creates a missed cleavage by the LysC enzyme, the peptides were found to be methylated at K18, K23, and K27 of the same peptide in a variety of permutations. Tri-methylation on K27 and K18 were easily detected, but the H3K23 tri-methyl lysine was not found in the first few runs. At that point, a permutation mass to charge list was created in Skyline (University of Washington) consisting of all peptides that contain a H3K23 tri-methylation in the +4, +5, or +6 and the list was imported as an inclusion list into the instrument acquisition parameters giving the expected peptides priority over any other *m*/*z*. The final method of acquisition ran with a 60-min gradient using a resolution of 120,000 for precursors and 60,000 for fragment ions. The instrument was run using ETD mode with a supplemental collision energy of 20 (EThcD). Since duty cycle was not an issue with the targeted run, the AGC target and maximum injection time were each set to 1000 giving maximum sensitivity for the inclusion masses.

### Mass spectrometry data analysis

Mass spec data were searched against the UniProt version 5640 Homo sapiens database using Mascot search engine version 2.8.0 through Proteome Discoverer version 2.5. The search parameters were set to include up to 5 missed cleavages with LysC as the enzyme and EThcD as the instrument type. Mass tolerances were set to 5 ppm for precursors and 20 ppm for fragments, although this was later filtered to 2 ppm for all conclusive spectra. Dynamic modifications were set as methyl K, di-methyl K, and tri-methyl K, in addition to oxidation on methionine and deamidation on asparagine and glutamine. Carbamidomethylation was set as a static modification. Database hits were filtered at the 1% FDR level using target-decoy validation.

### In vitro peptide pulldowns

To prepare beads for the peptide pulldown, 25 μL of M280 Streptavidin-conjugated Dynabeads (Invitrogen, Cat. # 11205D) were washed 3 times with 1X PBS-T (0.1% Triton-X 100) and resuspended in 500 μL of 1X PBS-T. 1 μg of biotinylated peptide, listed in Table 2, was added and allowed to incubate for 1 h at room temperature. Following peptide conjugation, the peptide-conjugated beads were washed with 1X PBS-T three times to displace any unconjugated peptide. Following the washes, 2 μg of the recombinantly expressed EHMT2/G9a (Active Motif 31410) or 5 μg of recombinantly expressed EHMT1/GLP (Active Motif 31920) was incubated with the peptide-conjugated dynabeads overnight at 4 ℃ with mild agitation. Following incubation, the beads were washed five times with buffer containing 300 mM KCl, 0.2% *v*/*v* Triton-X 100, 1 mM PMSF. Tubes containing the beads were changed on the fifth wash to eliminate any contaminating protein remaining on the sides of the tube. Following the tube-change, a sixth wash was performed. Following the last wash, the beads were resuspended in 1X SDS loading buffer, boiled and subjected to SDS-PAGE for analysis. Flag-tagged EHMT2/G9a was blotted using 1:10,000 anti-Flag antibody (Sigma F1804) followed by a 1:10,000 anti-mouse secondary incubation (GE Healthcare, NA9310). GST-tagged EHMT1/GLP was blotted using anti-GST antibody 1:10,000 (GE Healthcare, 27457701) followed by a rabbit anti-goat HRP secondary incubation (Invitrogen, A27014). The biotinylated peptides were blotted using streptavidin-HRP conjugate (Sigma, #RABHRP3, 1:10,000, 1 h room temperature). All western blots were imaged using an Acura Biosystems Imager.

### ELISA antibody validation

Histone methyl-lysine peptides, listed in Table 2, were serially diluted in high-bind, hydrophobic 96-well plates (Sigma, #M9410) and allowed to incubate 37 ℃, 100 rpm, overnight. The following day, plates were washed with 1X PBS twice, and blocked with 1X PBS containing 4% BSA for 2 h, 37 ℃. Following blocking, plates were washed twice with 1X PBS containing 0.15% Tween-20. All antibodies were diluted 1:10,000 in 1X PBS (containing 4% BSA and 0.15% *v*/*v* Tween-20), added to plates and allowed to incubate at 37 ℃ for 2 h. Peptide competition reactions were pre-incubated 2 h at room temperature with the respective antibody prior to addition to the ELISA plates. Following incubation with primary antibody incubation, the plates were washed 3X with 1X PBS containing 0.15% *v*/*v* Tween-20 and incubated with secondary antibody (1:10,000) for 2 h at 37 °C. Following incubation in secondary antibody, the plates were washed 3X with 1X PBS containing 0.15% *v*/*v* Tween-20. The plates were then incubated with buffer containing dibasic sodium phosphate (20 mM), citric acid (10 mM) and fresh hydrogen peroxide (final concentration: 0.0012%), and incubated for 30 min at room temperature in the dark. The reaction was quenched with 1 M H_2_SO_4_ and imaged on a spectrometer at 492 nm. All data are represented in Additional file 1: Fig. S6.

### Western blotting conditions for in vitro HMT and in vivo inhibition assays

- Anti-H3K9me1; Abcam 8896; 1:5,000
- Anti-H3K9me2; Active motif 39753; 1:5000
- Anti-H3K9me3; Abcam 8898; 1:5000
- Anti-H3K18me1; Diagenode C15410290; 1:500
- Anti-H3K18me2; Diagenode C15410291; 1:500
- Anti-H3K18me3; Diagenode C15410292; 1:500
- Anti-H3K23me1; Active motif 39387; 1:10,000 (1:500 for in vivo)
- Anti-H3K23me2; Abcam 214654; 1:2000
- Anti-H3K23me3; Active motif 61499; 1:500
- Anti-H3K27me1; Active motif 61015; 1:500
- Anti-H3K27me2; Active motif 61435; 1:1000
- Anti-H3K27me3; Millipore-Sigma 07-449; 1:5000
- Anti-EHMT1/GLP; Abcam ab41969; 1:500
- Anti-EHMT2/GLP; Abcam ab185050; 1:500
- Anti-Actin; Thermo Fisher MA1-91399; 1:2000
- Anti-H3; Abcam ab1791; 1:5000
- Anti-GST; GE Healthcare, 27457701; 1:10,000
- Anti-Flag; Sigma Aldrich F1804; 1:10,000.

## Supplementary Information

**Additional file 1: Figure S1.** EHMT1/GLP and EHMT2/G9a de novo methylate H3K18, H3K23, and H3K27, in vitro. (**A**) Annotated spectra, ions counts and mass error tables for the mass spectrometry analysis of EHMT1/GLP and EHMT2/G9a in vitro HMT reactions (**B**) Western blot panel of EHMT1/GLP and EHMT2/G9a in vitro HMT reactions for various H3K18 and H3K27 methylation states. H3K4me3 and H3K36me3 were included as negative controls. 50B11 whole cell lysate was used to validate that antibodies for H3K27me1/2, H3K4me3, and H3K36me3, were functional given these modifications were not detected by western blotting in EHMT1/GLP and EHMT2/G9a in vitro HMT reactions. **Figure S2. **Pharmacologic inhibition of EHMT1/GLP and EHMT2/G9a decreases H3K18 and H3K27 methylation in vitro. (**A**) UNC0642 dose titration with recombinant SetDB1 and Suv39h2, as measured by the MTase-Glo assay. (**B**) Western blotting panel showing production and inhibition of various H3K18 and H3K27 methylation states by EHMT1/GLP and EHMT2/G9a. Unlike H3K27me3, H3K27me1 and H3K27me2 were undetectable in this in vitro assay. **Figure S3.** Pharmacologic inhibition of EHMT1/GLP and EHMT2/G9a decrease H3K18 and H3K27 methylation in vivo. (**A**) Western blot panel of H3K9, H3K18, H3K23 and H3K27 methylation states in MC38 cells treated with DMSO or UNC0642. (**B**) Western blot panel of H3K18 and H3K27 methylation states of 50B11 cells treated with DMSO or UNC0642 (**C**)Western blot panel of H3K18 and H3K27 methylation states of HEK293 cells treated with DMSO or UNC0642. **Figure S4.** H3K9M, but not H3K23M, decrease H3K23me3 in vivo. (**A**) MC38 and (**B**) 50B11 cells lines were transduced with lentivirus carrying plasmids encoding various lysine (K) to methionine (M) mutations at specific residues (e.g. (, 23, 27, etc.) to evaluate their corresponding effect on various histone H3 methylation state in vivo. **Figure S5.** Genetic ablation of GLP, G9a or both perturbs H3K18 and H3K27 methylation in vivo. Western blot of H3K18 and H3K27 methylation states in mouse ESCs knocking down either EHMT1/GLP, EHMT2/G9a or both and their corresponding effect on H3K18 and h3K27 methylation states. **Figure S6.** Antibody validation via ELISA and peptide competition assays. Using a 96-well plate ELISA format, ELISAs and peptide competition assays were done to validate H3K9me1, H39me2, H3K9me3, H3K18me1, H3K18me2, H3K18me3, H3K23me1, H3K23me2 H3K23me3, H3K27me1, H3K27me2, and H3M27me3 antibodies for their target methylation state, cross-reactivity with other methylation states of the same epitope and cross-reactivity with methyl-lysines of a different epitope (e.g. K9 antibodies with K23-methyl peptides, etc.).

## Data Availability

The datasets generated and analyzed in this study are available from the corresponding author upon reasonable request.
