# Supplementary material for: De novo methylation of histone H3K23 by the methyltransferases EHMT1/GLP and EHMT2/G9a
Source: Epigenetics Chromatin. 2022 Nov 21;15:36. doi: 10.1186/s13072-022-00468-1 (PMC9677696; doi:10.1186/s13072-022-00468-1)
Supplement: Supplementary file 1 — Additional file 1: Figure S1. EHMT1/GLP and EHMT2/G9a de novo methylate H3K18, H3K23, and H3K27, in vitro. (A) Annotated spectra, ions counts and mass error tables for the mass spectrometry analysis of EHMT1/GLP and EHMT2/G9a in vitro HMT reactions (B) Western blot panel of EHMT1/GLP and EHMT2/G9a in vitro HMT reactions for various H3K18 and H3K27 methylation states. H3K4me3 and H3K36me3 were included as negative controls. 50B11 whole cell lysate was used to validate that antibodies for H3K27me1/2, H3K4me3, and H3K36me3, were functional given these modifications were not detected by western blotting in EHMT1/GLP and EHMT2/G9a in vitro HMT reactions. Figure S2. Pharmacologic inhibition of EHMT1/GLP and EHMT2/G9a decreases H3K18 and H3K27 methylation in vitro. (A) UNC0642 dose titration with recombinant SetDB1 and Suv39h2, as measured by the MTase-Glo assay. (B) Western blotting panel showing production and inhibition of various H3K18 and H3K27 methylation states by EHMT1/GLP and EHMT2/G9a. Unlike H3K27me3, H3K27me1 and H3K27me2 were undetectable in this in vitro assay. Figure S3. Pharmacologic inhibition of EHMT1/GLP and EHMT2/G9a decrease H3K18 and H3K27 methylation in vivo. (A) Western blot panel of H3K9, H3K18, H3K23 and H3K27 methylation states in MC38 cells treated with DMSO or UNC0642. (B) Western blot panel of H3K18 and H3K27 methylation states of 50B11 cells treated with DMSO or UNC0642 (C)Western blot panel of H3K18 and H3K27 methylation states of HEK293 cells treated with DMSO or UNC0642. Figure S4. H3K9M, but not H3K23M, decrease H3K23me3 in vivo. (A) MC38 and (B) 50B11 cells lines were transduced with lentivirus carrying plasmids encoding various lysine (K) to methionine (M) mutations at specific residues (e.g. (, 23, 27, etc.) to evaluate their corresponding effect on various histone H3 methylation state in vivo. Figure S5. Genetic ablation of GLP, G9a or both perturbs H3K18 and H3K27 methylation in vivo. Western blot of H3K18 and H3K27 methylation state [file 13072_2022_468_MOESM1_ESM.pdf]

A.

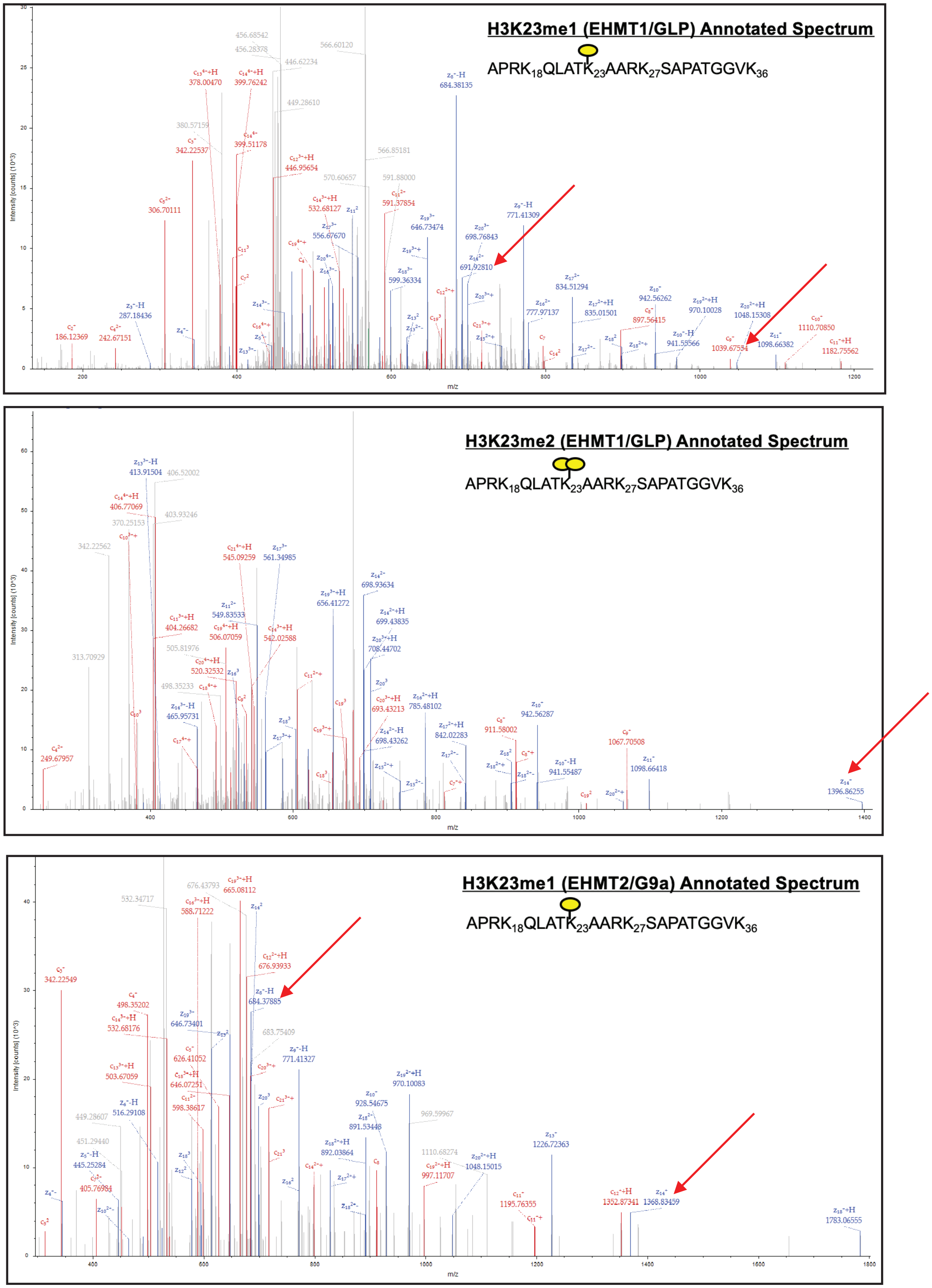

H3K23me1 (EHMT1/GLP) Annotated Spectrum
APRK$_{18}$QLATK$_{23}$AARK$_{27}$SAPATGGVK$_{36}$
H3K23me2 (EHMT1/GLP) Annotated Spectrum
APRK$_{18}$QLATK$_{23}$AARK$_{27}$SAPATGGVK$_{36}$
H3K23me1 (EHMT2/G9a) Annotated Spectrum
APRK$_{18}$QLATK$_{23}$AARK$_{27}$SAPATGGVK$_{36}$

H3K23me1 (EHMT1/GLP) Fragmentation EThcD

#1	c^{+}	$c{+}H^{3+}$	c^{2+}	c^{3+}	Seq.	z^{+}	z^{2+}	$z{-}H^{2+}$	$z{-}H^{3+}$	#2
1	89.07094	30.69777	45.03911	30.36183	A					22
2	186.12370	63.04869	93.56549	62.71275	P	2191.33973	1096.17350	1095.66959	730.78215	21
3	342.22481	115.08240	171.61605	114.74646	R	2094.28697	1047.64712	1047.14321	698.43123	20
4	484.33543	162.45260	242.67135	162.11666	K-Methyl	1938.18586	969.59657	969.09265	646.39753	19
5	612.39400	205.13879	306.70064	204.80285	Q	1796.07524	898.54126	898.03735	599.02732	18
6	725.47807	242.83348	363.24267	242.49754	L	1668.01667	834.51197	834.00806	556.34113	17
7	796.51518	266.51252	398.76123	266.17658	A	1554.93260	777.96994	777.46603	518.64644	16
8	897.56286	300.19508	449.28507	299.85914	T	1483.89549	742.45138	741.94747	494.96741	15
9	1039.67347	347.56528	520.34038	347.22934	K-Methyl	1382.84781	691.92754	691.42363	461.28485	14
10	1110.71059	371.24432	555.85893	370.90838	A	1240.73720	620.87224	620.36832	413.91464	13
11	1181.74770	394.92336	591.37749	394.58742	A	1169.70008	585.35368	584.84977	390.23560	12
12	1337.84881	446.95706	669.42804	446.62112	R	1098.66297	549.83512	549.33121	366.55657	11
13	1507.99073	503.67103	754.49900	503.33509	K-Trimethyl	942.56186	471.78457	471.28065	314.52286	10
14	1595.02275	532.68171	798.01502	532.34577	S	772.41994	386.71361	386.20970	257.80889	9
15	1666.05987	556.36075	833.53357	556.02481	A	685.38792	343.19760	342.69368	228.79821	8
16	1763.11263	588.71167	882.05995	588.37573	P	614.35080	307.67904	307.17513	205.11918	7
17	1834.14974	612.39071	917.57851	612.05477	A	517.29804	259.15266	258.64875	172.76826	6
18	1935.19742	646.07327	968.10235	645.73733	T	446.26092	223.63410	223.13019	149.08922	5
19	1992.21889	665.08042	996.61308	664.74448	G	345.21325	173.11026	172.60635	115.40666	4
20	2049.24035	684.08758	1025.12381	683.75163	G	288.19178	144.59953	144.09562	96.39950	3
21	2148.30876	717.11038	1074.65802	716.77444	V	231.17032	116.08880	115.58489	77.39235	2
22					K	132.10191	66.55459	66.05068	44.36954	1

H3K23me1 (EHMT1/GLP) Fragmentation Mass Error (ppm) EThcD

#1	c^{+}	$c{+}H^{3+}$	$c{+}H^{4+}$	c^{2+}	c^{3+}	Seq.	z^{+}	z^{2+}	$z{-}H^{2+}$	$z{-}H^{3+}$	#2
1						A					22
2	+0.07					P					21
3	-1.62					R					20
4	-1.93			-0.65		K-Methyl					19
5				-1.53		Q		-0.48	-0.83		18
6						L		-1.16	-0.58		17
7	-1.16			-1.53		A		-1.84	-1.54	-1.96	16
8	-1.44					T			-1.78	-1.36	15
9	-1.99					K-Methyl	+0.55	-0.81	-1.79	-1.59	14
10	+1.88				-0.75	A		+0.65	-1.41	-1.33	13
11				-1.78	-1.50	A		+0.60	-1.92	-1.55	12
12		+1.17				R	-0.78	+0.62		-1.46	11
13		+1.00	+1.04		-1.44	K-Trimethyl	-0.81		-1.92		10
14		+0.83	+1.71	-1.99		S					9
15		-1.48				A					8
16		+0.53	+1.29			P					7
17					-1.58	A					6
18		+0.89	+0.58		+0.04	T					5
19		-0.31	+0.37	+0.04	-1.88	G					4
20		-0.46	+0.59			G					3
21		-1.32	+0.36		-1.24	V					2
22						K					1

H3K23me2 (EHMT1/GLP) Fragmentation EThcD

#1	c^{+}	$c+H^{+}$	c^{2+}	c^{3+}	Seq.	z^{+}	z^{2+}	z^{3+}	$z-H^{+}$	$z-H^{2+}$	$z-H^{3+}$	#2
1	89.07094	90.07876	45.03911	30.36183	A							22
2	186.12370	187.13153	93.56549	62.71275	P	2219.37103	1110.18915	740.46186	2218.36321	1109.68524	740.12592	21
3	342.22481	343.23264	171.61605	114.74646	R	2122.31827	1061.66277	708.11094	2121.31044	1061.15886	707.77500	20
4	498.35108	499.35890	249.67918	166.78854	K-Dimethyl	1966.21716	983.61222	656.07724	1965.20933	983.10830	655.74129	19
5	626.40965	627.41748	313.70847	209.47474	Q	1810.09089	905.54908	604.03515	1809.08307	905.04517	603.69921	18
6	739.49372	740.50154	370.25050	247.16942	L	1682.03232	841.51980	561.34896	1681.02449	841.01588	561.01301	17
7	810.53083	811.53866	405.76905	270.84846	A	1568.94825	784.97776	523.65427	1567.94043	784.47385	523.31833	16
8	911.57851	912.58634	456.29289	304.53102	T	1497.91114	749.45921	499.97523	1496.90331	748.95529	499.63929	15
9	1067.70477	1068.71260	534.35603	356.57311	K-Dimethyl	1396.86346	698.93537	466.29267	1395.85563	698.43146	465.95673	14
10	1138.74189	1139.74971	569.87458	380.25215	A	1240.73720	620.87224	414.25058	1239.72937	620.36832	413.91464	13
11	1209.77900	1210.78683	605.39314	403.93118	A	1169.70008	585.35368	390.57155	1168.69226	584.84977	390.23560	12
12	1365.88011	1366.88794	683.44369	455.96489	R	1098.66297	549.83512	366.89251	1097.65514	549.33121	366.55657	11
13	1536.02203	1537.02985	768.51465	512.67886	K-Trimethyl	942.56186	471.78457	314.85880	941.55403	471.28065	314.52286	10
14	1623.05405	1624.06188	812.03067	541.68954	S	772.41994	386.71361	258.14483	771.41212	386.20970	257.80889	9
15	1694.09117	1695.09899	847.54922	565.36857	A	685.38792	343.19760	229.13416	684.38009	342.69368	228.79821	8
16	1791.14393	1792.15176	896.07560	597.71949	P	614.35080	307.67904	205.45512	613.34298	307.17513	205.11918	7
17	1862.18104	1863.18887	931.59416	621.39853	A	517.29804	259.15266	173.10420	516.29021	258.64875	172.76826	6
18	1963.22872	1964.23655	982.11800	655.08109	T	446.26092	223.63410	149.42516	445.25310	223.13019	149.08922	5
19	2020.25019	2021.25801	1010.62873	674.08825	G	345.21325	173.11026	115.74260	344.20542	172.60635	115.40666	4
20	2077.27165	2078.27948	1039.13946	693.09540	G	288.19178	144.59953	96.73545	287.18396	144.09562	96.39950	3
21	2176.34006	2177.34789	1088.67367	726.11821	V	231.17032	116.08880	77.72829	230.16249	115.58489	77.39235	2
22					K	132.10191	66.55459	44.70549	131.09408	66.05068	44.36954	1

H3K23me2 (EHMT1/GLP) Fragmentation Mass Error (ppm) EThcD

#1	c^{+}	$c+H^{+}$	c^{2+}	c^{3+}	Seq.	z^{+}	z^{2+}	$z-H^{+}$	$z-H^{2+}$	$z-H^{3+}$	#2
1					A						22
2	+0.50				P						21
3	-0.10				R		-0.30			-0.89	20
4	-0.43				K-Dimethyl		-0.72				19
5	-0.81		-1.64		Q		+0.89		-1.41	-1.13	18
6			-0.06		L	+0.56	+0.83	+1.14	-0.06	-0.95	17
7	-0.52		-0.95		A	-1.24	+1.92		-0.34		16
8	-0.38		-1.37		T			+0.73	-0.77	+1.00	15
9		+0.76	-1.13		K-Dimethyl	+0.92	+1.93	+0.73	-0.61	+0.21	14
10		-0.58	-1.05		A	+0.11		+1.28	-0.62		13
11	+0.66		-0.99		A	+0.84			-0.36		12
12	+0.08	+1.61	-0.75	-0.64	R	-0.22		+1.21	-0.95		11
13			-0.16	-0.55	K-Trimethyl	+1.45		-0.18	-0.10		10
14			+0.18	-0.86	S			+0.32			9
15					A			-0.50			8
16			+0.18	-0.90	P						7
17			-0.68	-0.83	A			-0.38			6
18			+1.76	-1.16	T			+0.99			5
19			+1.22	-0.64	G			-0.34			4
20			-0.53	-0.52	G	-0.16					3
21				-1.04	V						2
22					K			+0.46			1

H3K23me3 (EHMT1/GLP) Fragmentation EThcD

#1	c^{2+}	c^{3+}	c^{4+}	Seq.	z^{2+}	z^{3+}	$z\text{-}H^{2+}$	$z\text{-}H^{3+}$	#2
1	45.03911	30.36183	23.02319	A					22
2	93.56549	62.71275	47.28638	P	1124.20480	749.80563	1123.70089	749.46969	21
3	171.61605	114.74646	86.31166	R	1075.67842	717.45471	1075.17451	717.11876	20
4	256.68700	171.46043	128.84714	K-Trimethyl	997.62787	665.42100	997.12395	665.08506	19
5	320.71629	214.14662	160.86178	Q	912.55691	608.70703	912.05300	608.37109	18
6	377.25832	251.84131	189.13280	L	848.52762	566.02084	848.02371	565.68490	17
7	412.77688	275.52035	206.89208	A	791.98559	528.32615	791.48168	527.99021	16
8	463.30072	309.20290	232.15400	T	756.46703	504.64711	755.96312	504.31117	15
9	548.37168	365.91688	274.68948	K-Trimethyl	705.94319	470.96455	705.43928	470.62861	14
10	583.89023	389.59591	292.44875	A	620.87224	414.25058	620.36832	413.91464	13
11	619.40879	413.27495	310.20803	A	585.35368	390.57155	584.84977	390.23560	12
12	697.45934	465.30866	349.23331	R	549.83512	366.89251	549.33121	366.55657	11
13	782.53030	522.02263	391.76879	K-Trimethyl	471.78457	314.85880	471.28065	314.52286	10
14	826.04632	551.03330	413.52680	S	386.71361	258.14483	386.20970	257.80889	9
15	861.56487	574.71234	431.28607	A	343.19760	229.13416	342.69368	228.79821	8
16	910.09125	607.06326	455.54927	P	307.67904	205.45512	307.17513	205.11918	7
17	945.60981	630.74230	473.30854	A	259.15266	173.10420	258.64875	172.76826	6
18	996.13365	664.42486	498.57046	T	223.63410	149.42516	223.13019	149.08922	5
19	1024.64438	683.43201	512.82583	G	173.11026	115.74260	172.60635	115.40666	4
20	1053.15511	702.43917	527.08120	G	144.59953	96.73545	144.09562	96.39950	3
21	1102.68932	735.46197	551.84830	V	116.08880	77.72829	115.58489	77.39235	2
22				K	66.55459	44.70549	66.05068	44.36954	1

H3K23me3 (EHMT1/GLP) Fragmentation Mass Error (ppm) EThcD

#1	c^{2+}	c^{3+}	c^{4+}	Seq.	z^{2+}	z^{3+}	$z\text{-}H^{2+}$	$z\text{-}H^{3+}$	#2
1				A					22
2				P					21
3				R		+0.93			20
4	+0.55			K-Trimethyl	-1.35	-0.67	-1.48	-1.86	19
5	+0.03			Q	+0.10	+1.30	-1.19	-1.22	18
6	-0.26			L	+0.04	+1.66	-1.33	-0.82	17
7	-0.03			A	+0.76	+1.80	-0.17	+0.08	16
8	-0.59			T	+0.07	+1.26	+0.87	-0.15	15
9	+0.17	-0.15		K-Trimethyl	-0.32		-1.37	-0.36	14
10	-0.36	-1.09		A					13
11	-0.83	-0.41		A			+1.62		12
12	-0.45	-0.59		R			-0.60		11
13		-0.74	+0.61	K-Trimethyl			+0.03		10
14		-0.92	-0.23	S					9
15				A					8
16		+0.55	-1.26	P					7
17		-0.70		A					6
18		-1.66	-0.25	T					5
19		-0.80	+0.99	G					4
20		+1.93	-0.09	G					3
21			-0.38	V					2
22				K					1

H3K23me1 (EHMT2/G9a) Fragmentation EThcD

#1	c^{+}	$c{+}H^{+}$	$c{+}H^{2+}$	$c{+}H^{3+}$	Seq.	z^{+}	z^{2+}	z^{3+}	$z{-}H^{2+}$	#2
1	89.07094	90.07876	45.54302	30.69777	A					22
2	186.12370	187.13153	94.06940	63.04869	P	2191.33973	1096.17350	731.11809	1095.66959	21
3	342.22481	343.23264	172.11996	115.08240	R	2094.28697	1047.64712	698.76717	1047.14321	20
4	498.35108	499.35890	250.18309	167.12449	K-Dimethyl	1938.18586	969.59657	646.73347	969.09265	19
5	626.40965	627.41748	314.21238	209.81068	Q	1782.05959	891.53343	594.69138	891.02952	18
6	739.49372	740.50154	370.75441	247.50537	L	1654.00102	827.50415	552.00519	827.00023	17
7	810.53083	811.53866	406.27297	271.18440	A	1540.91695	770.96211	514.31050	770.45820	16
8	911.57851	912.58634	456.79681	304.86696	T	1469.87984	735.44356	490.63146	734.93964	15
9	1053.68912	1054.69695	527.85211	352.23717	K-Methyl	1368.83216	684.91972	456.94890	684.41581	14
10	1124.72624	1125.73406	563.37067	375.91621	A	1226.72155	613.86441	409.57870	613.36050	13
11	1195.76335	1196.77118	598.88923	399.59524	A	1155.68443	578.34585	385.89966	577.84194	12
12	1351.86446	1352.87229	676.93978	451.62895	R	1084.64732	542.82730	362.22062	542.32339	11
13	1507.99073	1508.99855	755.00291	503.67103	K-Dimethyl	928.54621	464.77674	310.18692	464.27283	10
14	1595.02275	1596.03058	798.51893	532.68171	S	772.41994	386.71361	258.14483	386.20970	9
15	1666.05987	1667.06769	834.03748	556.36075	A	685.38792	343.19760	229.13416	342.69368	8
16	1763.11263	1764.12046	882.56387	588.71167	P	614.35080	307.67904	205.45512	307.17513	7
17	1834.14974	1835.15757	918.08242	612.39071	A	517.29804	259.15266	173.10420	258.64875	6
18	1935.19742	1936.20525	968.60626	646.07327	T	446.26092	223.63410	149.42516	223.13019	5
19	1992.21889	1993.22671	997.11699	665.08042	G	345.21325	173.11026	115.74260	172.60635	4
20	2049.24035	2050.24818	1025.62773	684.08758	G	288.19178	144.59953	96.73545	144.09562	3
21	2148.30876	2149.31659	1075.16193	717.11038	V	231.17032	116.08880	77.72829	115.58489	2
22					K	132.10191	66.55459	44.70549	66.05068	1

H3K23me1 (EHMT2/G9a) Fragmentation Mass Error (ppm) EThcD

#1	c^{+}	$c{+}H^{+}$	$c{+}H^{2+}$	$c{+}H^{3+}$	Seq.	z^{+}	z^{2+}	z^{3+}	$z{-}H^{2+}$	#2
1					A					22
2					P					21
3	-1.97				R			-1.88		20
4	-1.89				K-Dimethyl			-0.84		19
5	-1.38				Q		-1.17	-1.78	-0.30	18
6					L		-1.78			17
7					A		-0.53			16
8	-1.38	-0.63			T					15
9					K-Methyl	-1.78	+0.95			14
10					A	-1.70	+1.04			13
11	-0.17	+1.89			A		+1.53			12
12		-0.83	+0.67	+1.43	R					11
13				+0.88	K-Dimethyl	-0.58			0.00	10
14			-0.07	-0.09	S					9
15					A					8
16				-0.93	P					7
17					A					6
18				+1.17	T					5
19			-0.08	-1.05	G					4
20				-1.00	G					3
21				+0.38	V					2
22					K					1

H3K23me2 (EHMT2/G9a) Fragmentation EThcD

#1	c+H^{2+}	c+H^{3+}	c+H^{4+}	c^{2+}	Seq.	z^{+}	z+H^{2+}	z+H^{3+}	z^{2+}	#2
1	45.54302	30.69777	23.27515	45.03911	A					22
2	94.06940	63.04869	47.53834	93.56549	P	2219.37103	1110.69307	740.79780	1110.18915	21
3	172.11996	115.08240	86.56362	171.61605	R	2122.31827	1062.16668	708.44688	1061.66277	20
4	250.18309	167.12449	125.59518	249.67918	K-Dimethyl	1966.21716	984.11613	656.41318	983.61222	19
5	314.21238	209.81068	157.60983	313.70847	Q	1810.09089	906.05300	604.37109	905.54908	18
6	370.75441	247.50537	185.88084	370.25050	L	1682.03232	842.02371	561.68490	841.51980	17
7	406.27297	271.18440	203.64012	405.76905	A	1568.94825	785.48168	523.99021	784.97776	16
8	456.79681	304.86696	228.90204	456.29289	T	1497.91114	749.96312	500.31117	749.45921	15
9	534.85994	356.90905	267.93361	534.35603	K-Dimethyl	1396.86346	699.43928	466.62861	698.93537	14
10	570.37849	380.58809	285.69289	569.87458	A	1240.73720	621.37615	414.58652	620.87224	13
11	605.89705	404.26713	303.45216	605.39314	A	1169.70008	585.85759	390.90749	585.35368	12
12	683.94761	456.30083	342.47744	683.44369	R	1098.66297	550.33904	367.22845	549.83512	11
13	769.01856	513.01480	385.01292	768.51465	K-Trimethyl	942.56186	472.28848	315.19475	471.78457	10
14	812.53458	542.02548	406.77093	812.03067	S	772.41994	387.21752	258.48077	386.71361	9
15	848.05313	565.70452	424.53021	847.54922	A	685.38792	343.70151	229.47010	343.19760	8
16	896.57952	598.05544	448.79340	896.07560	P	614.35080	308.18295	205.79106	307.67904	7
17	932.09807	621.73447	466.55267	931.59416	A	517.29804	259.65657	173.44014	259.15266	6
18	982.62191	655.41703	491.81459	982.11800	T	446.26092	224.13801	149.76110	223.63410	5
19	1011.13264	674.42419	506.06996	1010.62873	G	345.21325	173.61417	116.07854	173.11026	4
20	1039.64338	693.43134	520.32533	1039.13946	G	288.19178	145.10344	97.07139	144.59953	3
21	1089.17758	726.45415	545.09243	1088.67367	V	231.17032	116.59271	78.06423	116.08880	2
22					K	132.10191	67.05850	45.04143	66.55459	1

H3K23me2 (EHMT2/G9a) Fragmentation Mass Error (ppm) EThcD

#1	c+H^{2+}	c+H^{3+}	c+H^{4+}	c^{2+}	Seq.	z^{+}	z+H^{2+}	z+H^{3+}	z^{2+}	#2
1					A					22
2					P					21
3					R		-1.10	-0.20		20
4				-1.57	K-Dimethyl			+0.70		19
5					Q		+0.49		+0.28	18
6					L		+1.04	+1.67		17
7					A		+0.84			16
8					T		+0.23			15
9	+1.51			+0.23	K-Dimethyl	+0.65	+1.33		-1.39	14
10		-0.11			A		-0.61		-0.42	13
11	+0.53	+0.76			A				-0.04	12
12	+0.86				R	-1.10			-0.38	11
13		-0.29			K-Trimethyl	-1.07				10
14		-0.74	+0.58		S					9
15					A					8
16					P					7
17		+0.55	+1.77		A					6
18		-1.25	-1.21		T					5
19		-0.64	-1.24	+1.28	G					4
20		-1.14	+0.01		G					3
21			-0.29		V					2
22					K					1

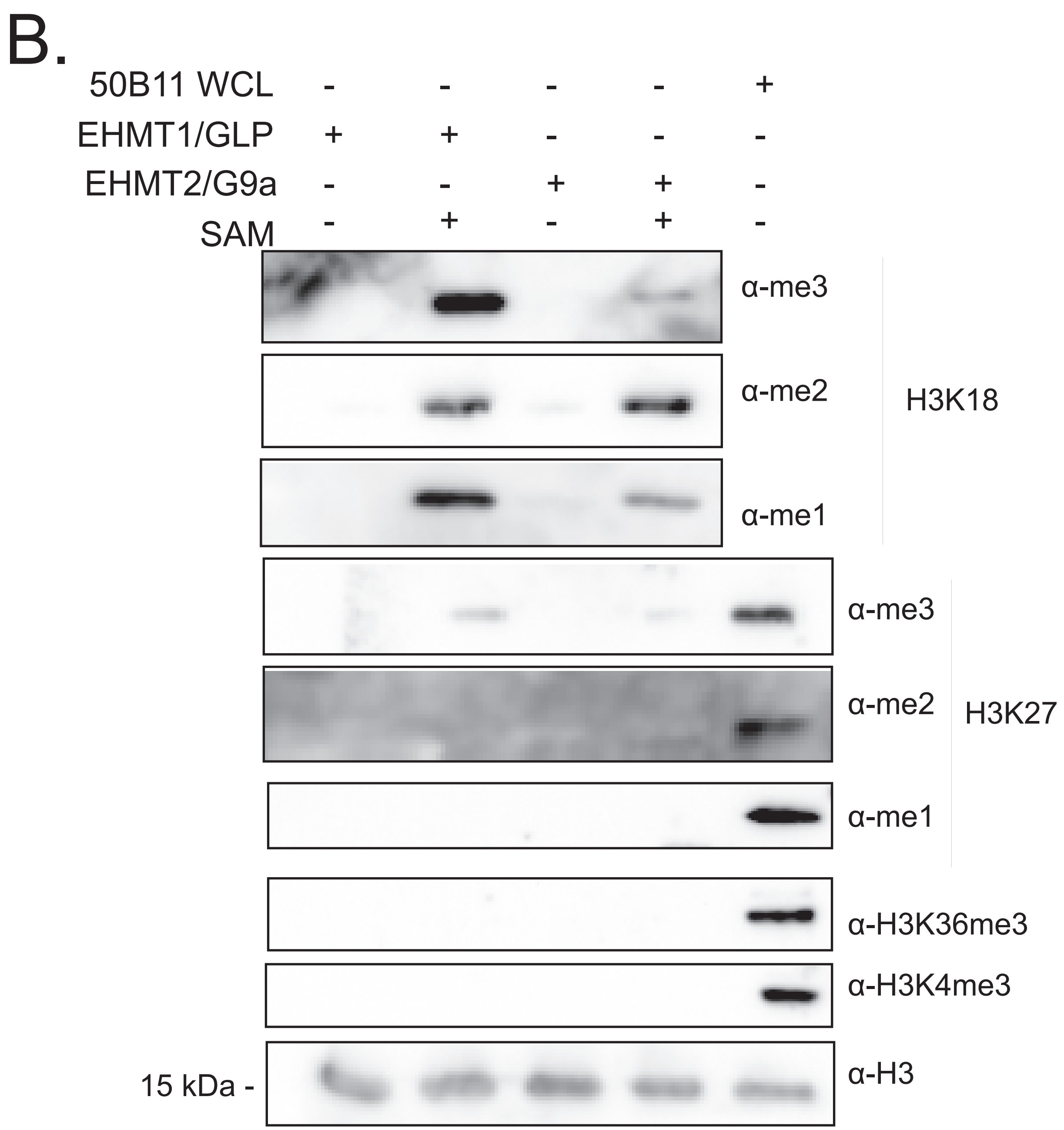

Fig. S1. EHMT1/GLP and EHMT2/G9a de novo methylate H3K18, H3K23, and H3K27, in vitro.

(A) Annotated spectra, ions counts and mass error tables for the mass spectrometry analysis of EHMT1/GLP and EHMT2/G9a in vitro HMT reactions **(B)** Western blot panel of EHMT1/GLP and EHMT2/G9a in vitro HMT reactions for various H3K18 and H3K27 methylation states. H3K4me3 and H3K36me3 were included as negative controls. 50B11 whole cell lysate was used to validate that antibodies for H3K27me1/2, H3K4me3, and H3K36me3, were functional given these modifications were not detected by western blotting in EHMT1/GLP and EHMT2/G9a in vitro HMT reactions.

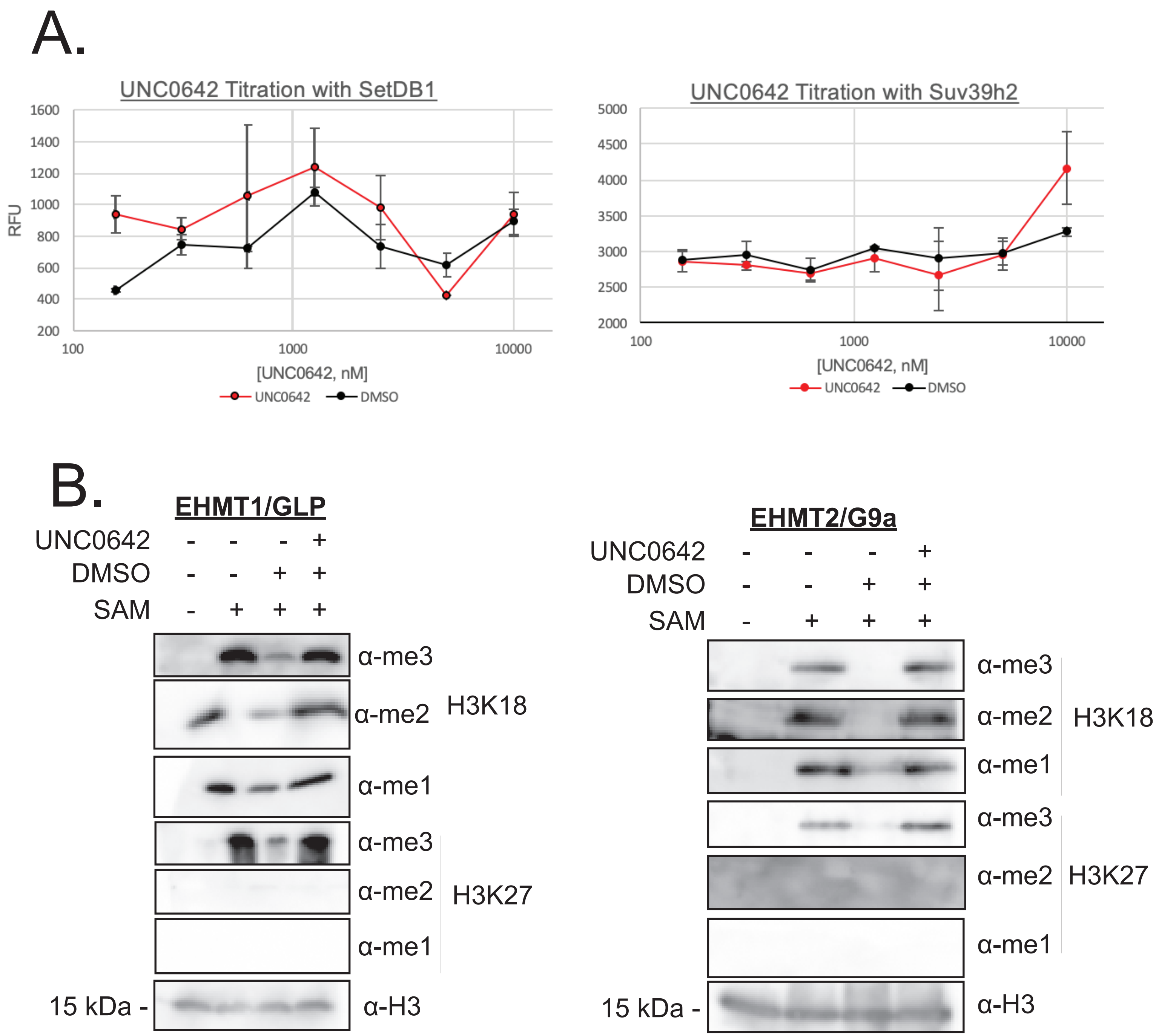

Fig S2. Pharmacologic inhibition of EHMT1/GLP and EHMT2/G9a decreases H3K18 and H3K27 methylation *in vitro*. (A) UNC0642 dose titration with recombinant SetDB1 and Suv39h2, as measured by the MTase-Glo assay. **(B)** Western blotting panel showing production and inhibition of various H3K18 and H3K27 methylation states by EHMT1/GLP and EHMT2/G9a. Unlike H3K27me3, H3K27me1 and H3K27me2 were undetectable in this in vitro assay.

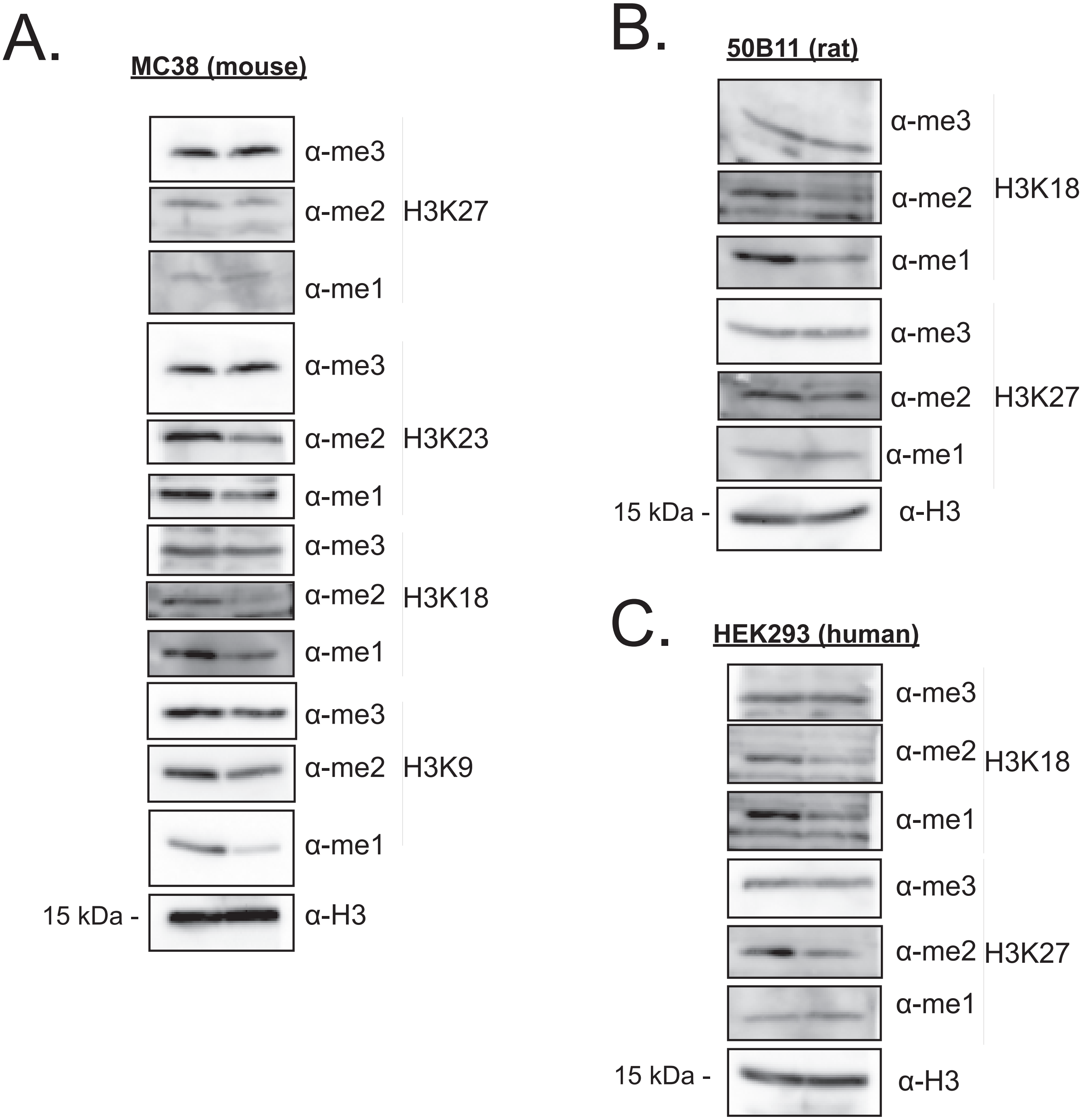

Fig. S3. Pharmacologic inhibition of EHMT1/GLP and EHMT2/G9a decrease H3K18 and H3K27 methylation *in vivo*. (A) Western blot panel of H3K9, H3K18, H3K23 and H3K27 methylation states in MC38 cells treated with DMSO or UNC0642. **(B)** Western blot panel of H3K18 and H3K27 methylation states of 50B11 cells treated iwth DMSO or UNC0642 **(C)**Western blot panel of H3K18 and H3K27 methylation states of HEK293 cells treated iwth DMSO or UNC0642

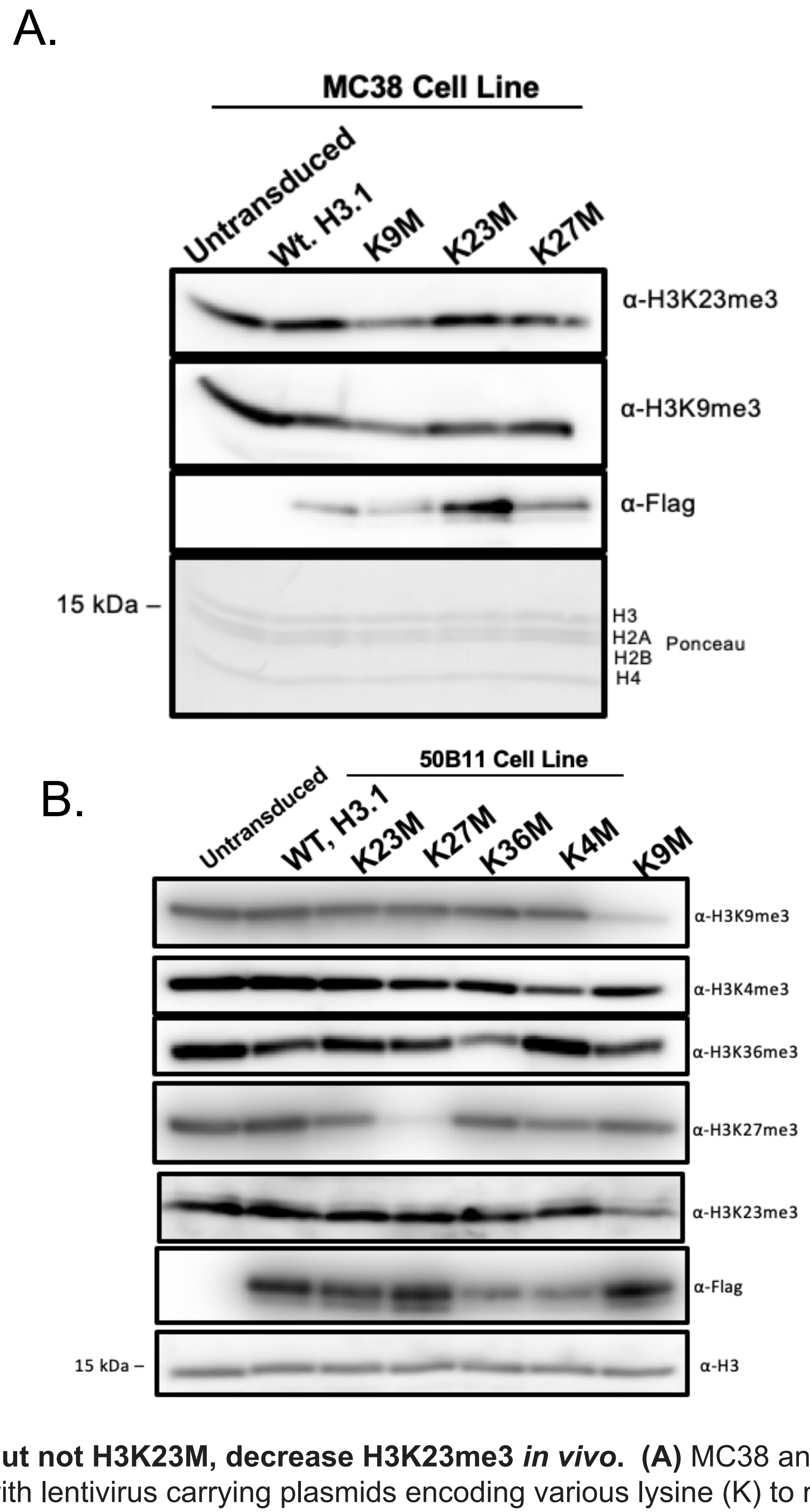

Fig. S4. H3K9M, but not H3K23M, decrease H3K23me3 *in vivo*. (A) MC38 and **(B)** 50B11 cells lines were transduced with lentivirus carrying plasmids encoding various lysine (K) to methionine (M) mutations at specific residues (e.g. (, 23, 27, etc.) to evaluate their corresponding effect on various histone H3 methylation state *in vivo*.

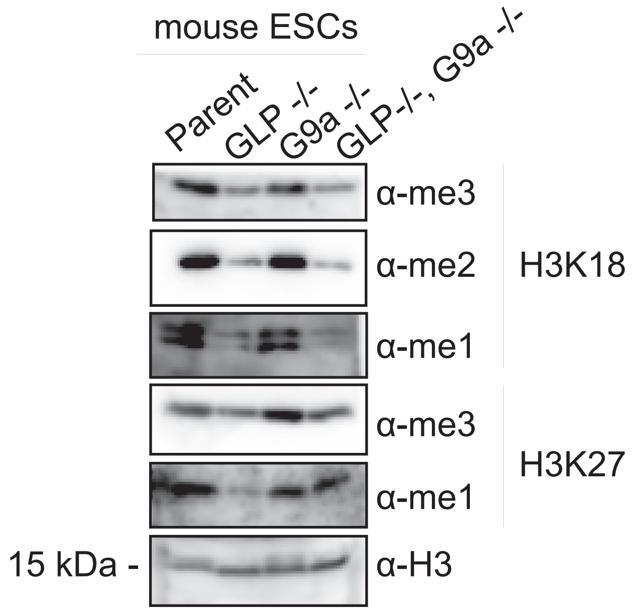

Fig. S5. Genetic ablation of GLP, G9a or both perturbs H3K18 and H3K27 methylation *in vivo*. Western blot of H3K18 and H3K27 methylation states in mouse ESCs knocking down either EHMT1/GLP, EHMT2/G9a or both and their corresponding effect on H3K18 and h3K27 methylation states

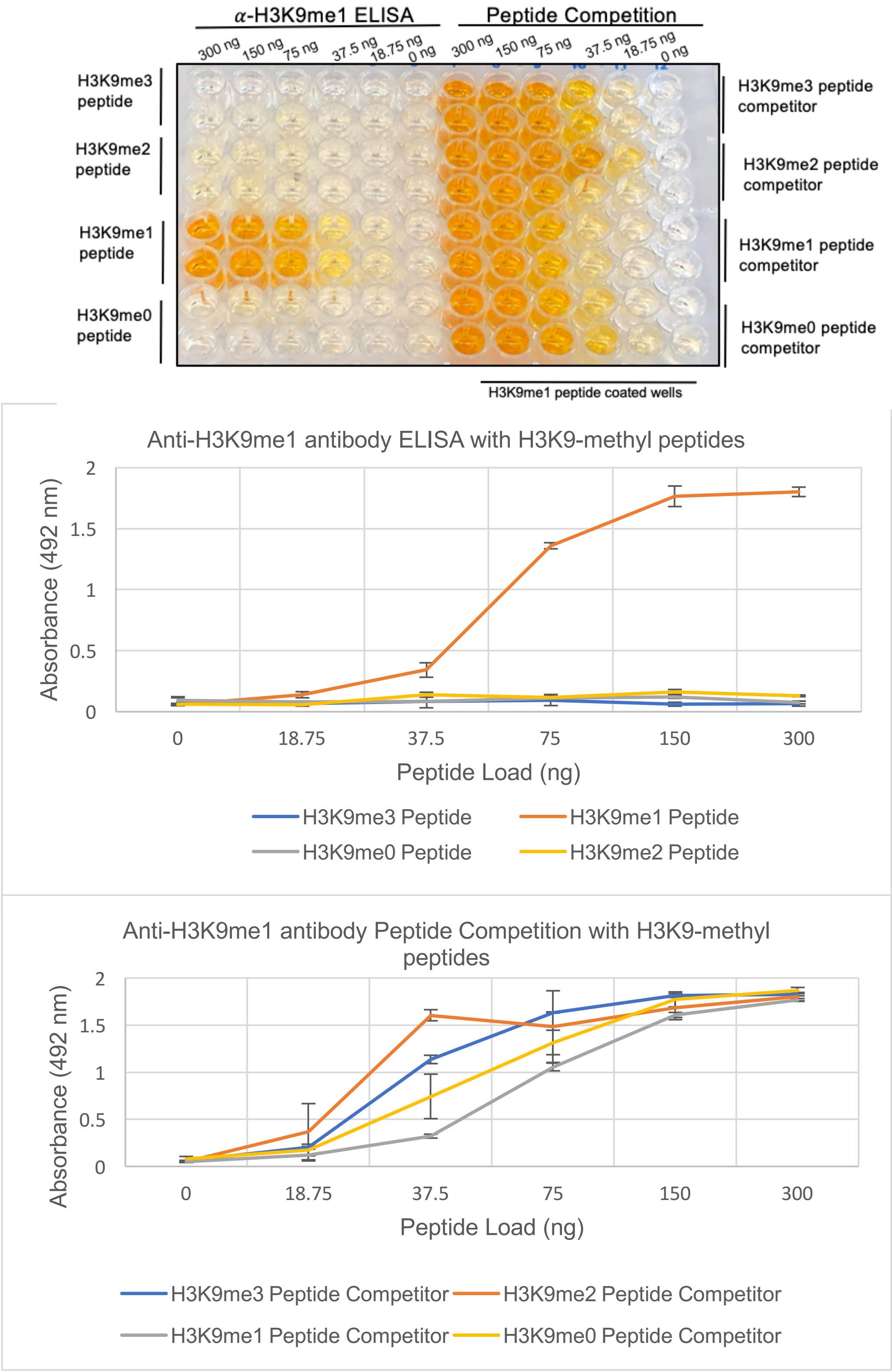
α-H3K9me1 ELISA
Peptide Competition
300 ng
150 ng
75 ng
37.5 ng
18.75 ng
0 ng
300 ng
150 ng
75 ng
37.5 ng
18.75 ng
0 ng
H3K9me3 peptide
H3K9me2 peptide
H3K9me1 peptide
H3K9me0 peptide
H3K9me3 peptide competitor
H3K9me2 peptide competitor
H3K9me1 peptide competitor
H3K9me0 peptide competitor
H3K9me1 peptide coated wells
Anti-H3K9me1 antibody ELISA with H3K9-methyl peptides
Absorbance (492 nm)
2
1.5
1
0.5
0
0
18.75
37.5
75
150
300
Peptide Load (ng)
H3K9me3 Peptide
H3K9me1 Peptide
H3K9me0 Peptide
H3K9me2 Peptide
Anti-H3K9me1 antibody Peptide Competition with H3K9-methyl peptides
Absorbance (492 nm)
2
1.5
1
0.5
0
0
18.75
37.5
75
150
300
Peptide Load (ng)
H3K9me3 Peptide Competitor
H3K9me2 Peptide Competitor
H3K9me1 Peptide Competitor
H3K9me0 Peptide Competitor

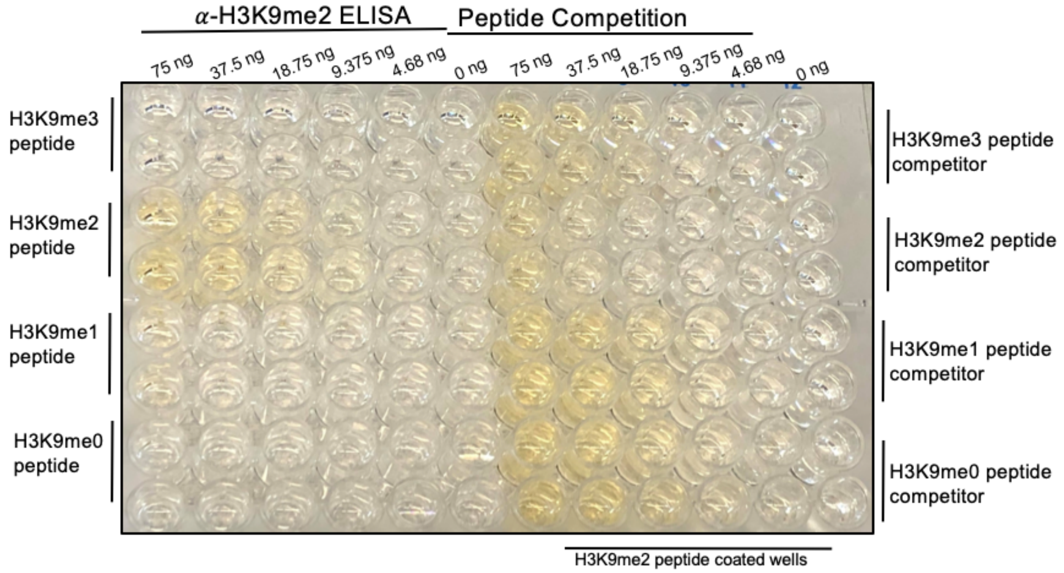
α-H3K9me2 ELISA
Peptide Competition
75 ng
37.5 ng
18.75 ng
9.375 ng
4.68 ng
0 ng
75 ng
37.5 ng
18.75 ng
9.375 ng
4.68 ng
0 ng
H3K9me3 peptide
H3K9me2 peptide
H3K9me1 peptide
H3K9me0 peptide
H3K9me3 peptide competitor
H3K9me2 peptide competitor
H3K9me1 peptide competitor
H3K9me0 peptide competitor
H3K9me2 peptide coated wells

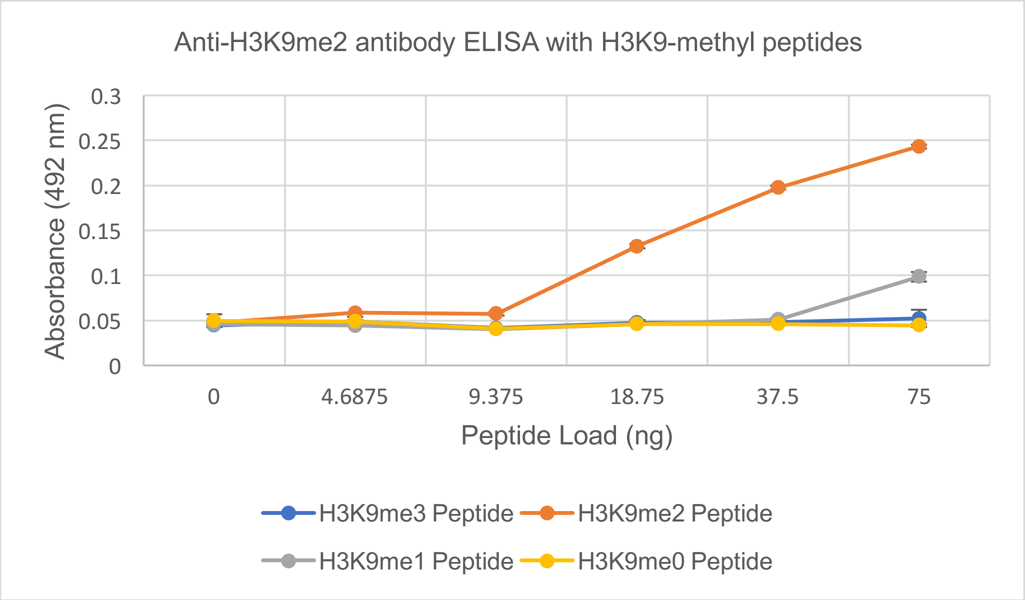
Anti-H3K9me2 antibody ELISA with H3K9-methyl peptides
Absorbance (492 nm)
0.3
0.25
0.2
0.15
0.1
0.05
0
0
4.6875
9.375
18.75
37.5
75
Peptide Load (ng)
H3K9me3 Peptide
H3K9me2 Peptide
H3K9me1 Peptide
H3K9me0 Peptide

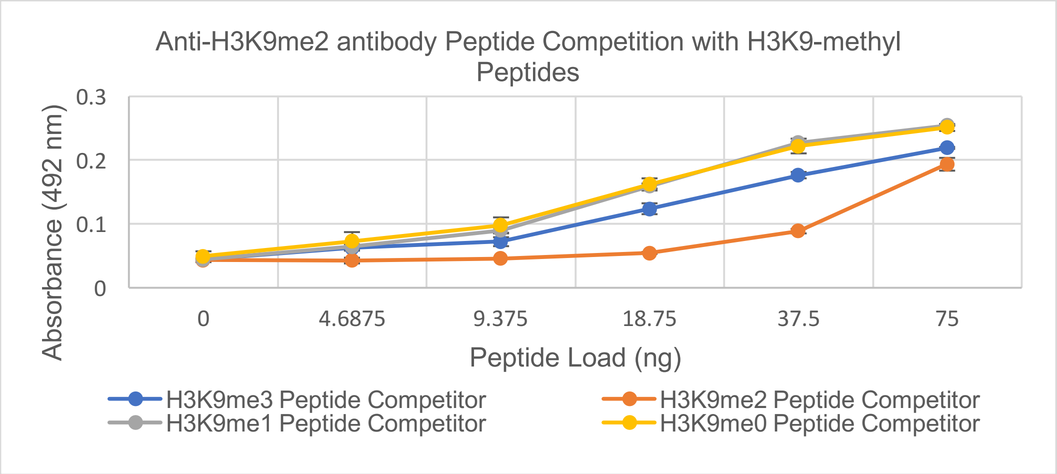
Anti-H3K9me2 antibody Peptide Competition with H3K9-methyl Peptides
Absorbance (492 nm)
0.3
0.2
0.1
0
0
4.6875
9.375
18.75
37.5
75
Peptide Load (ng)
H3K9me3 Peptide Competitor
H3K9me2 Peptide Competitor
H3K9me1 Peptide Competitor
H3K9me0 Peptide Competitor

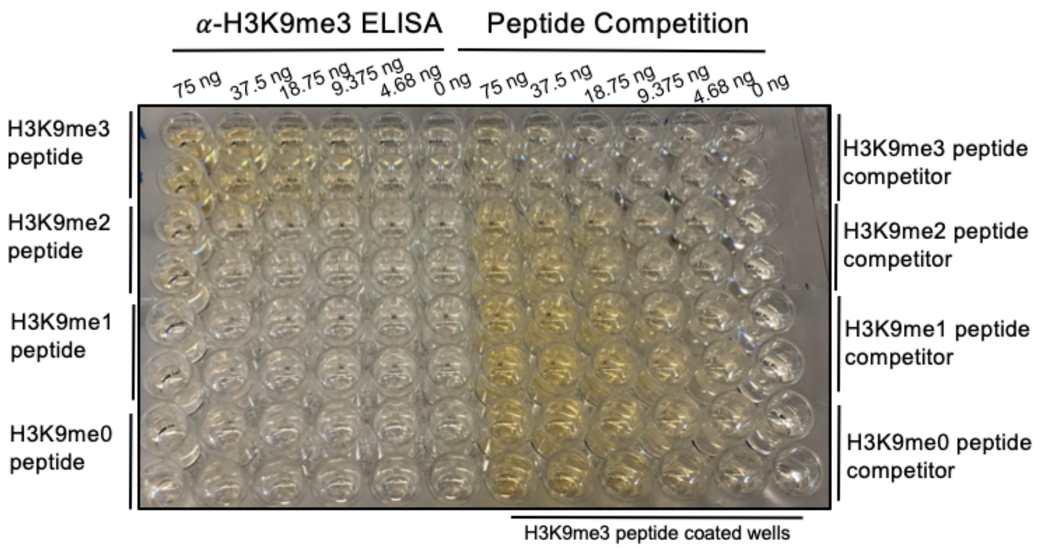

α-H3K9me3 ELISA
Peptide Competition
75 ng
37.5 ng
18.75 ng
9.375 ng
4.68 ng
0 ng
75 ng
37.5 ng
18.75 ng
9.375 ng
4.68 ng
0 ng
H3K9me3 peptide
H3K9me2 peptide
H3K9me1 peptide
H3K9me0 peptide
H3K9me3 peptide competitor
H3K9me2 peptide competitor
H3K9me1 peptide competitor
H3K9me0 peptide competitor
H3K9me3 peptide coated wells

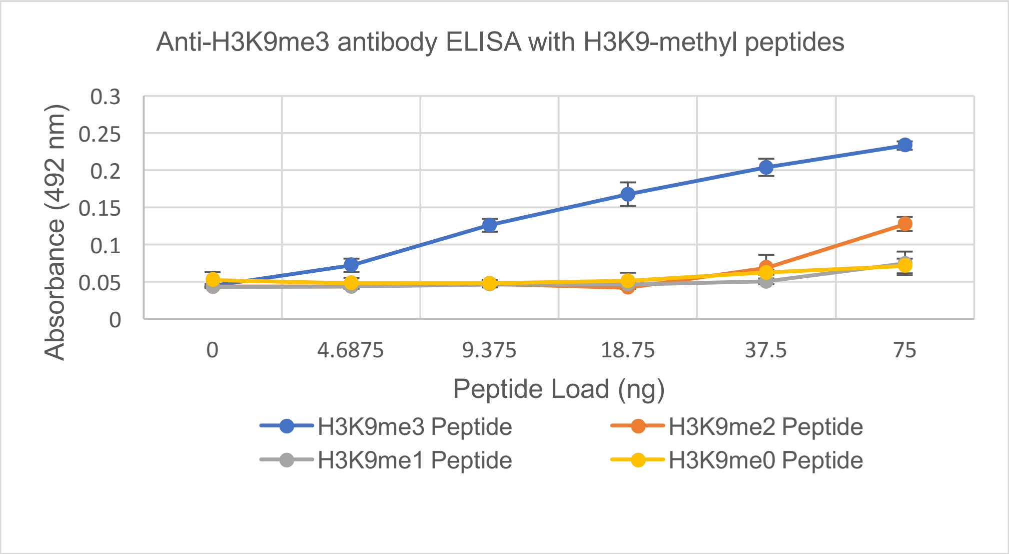

Anti-H3K9me3 antibody ELISA with H3K9-methyl peptides
Absorbance (492 nm)
0.3
0.25
0.2
0.15
0.1
0.05
0
0
4.6875
9.375
18.75
37.5
75
Peptide Load (ng)
H3K9me3 Peptide
H3K9me2 Peptide
H3K9me1 Peptide
H3K9me0 Peptide

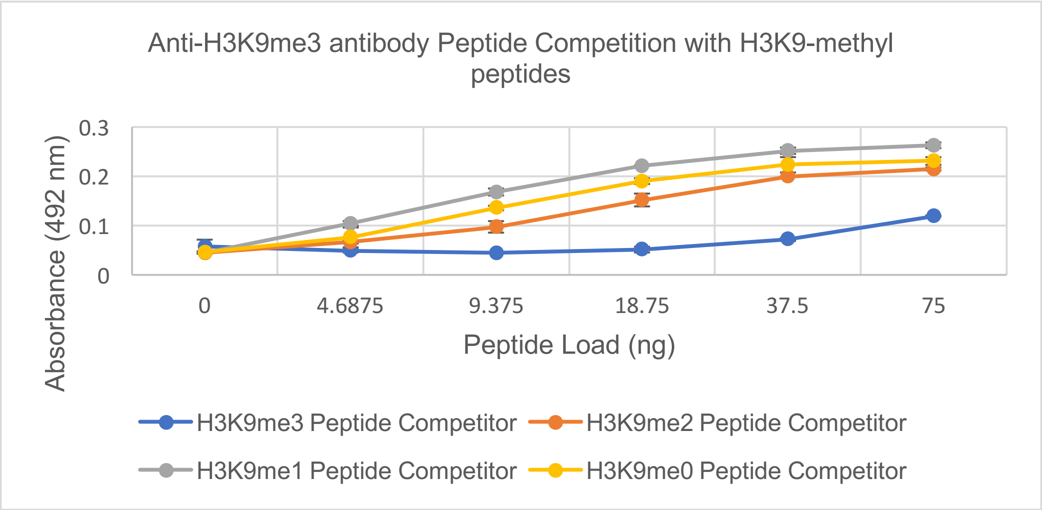

Anti-H3K9me3 antibody Peptide Competition with H3K9-methyl peptides
Absorbance (492 nm)
0.3
0.2
0.1
0
0
4.6875
9.375
18.75
37.5
75
Peptide Load (ng)
H3K9me3 Peptide Competitor
H3K9me2 Peptide Competitor
H3K9me1 Peptide Competitor
H3K9me0 Peptide Competitor

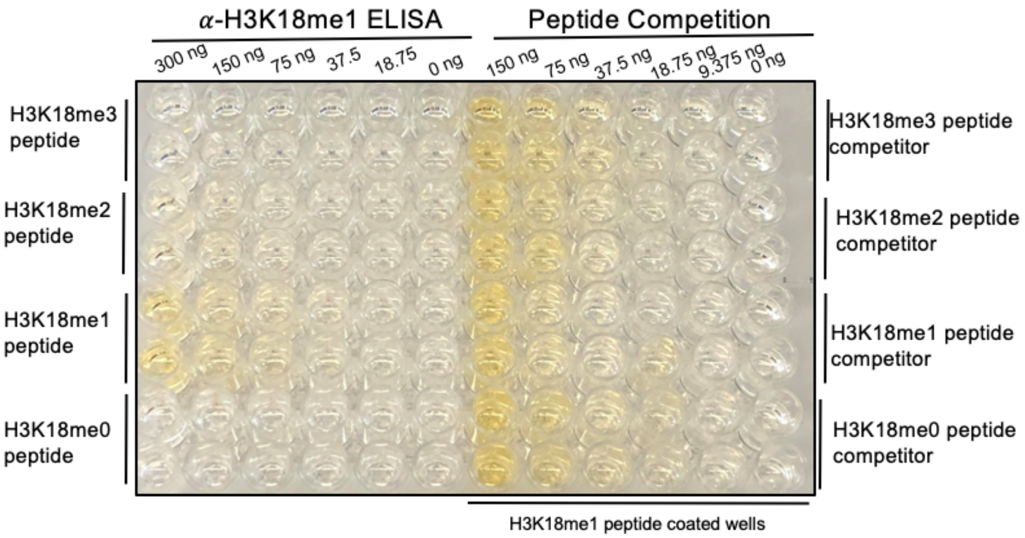
α-H3K18me1 ELISA
Peptide Competition
300 ng
150 ng
75 ng
37.5
18.75
0 ng
150 ng
75 ng
37.5 ng
18.75 ng
9.375 ng
0 ng
H3K18me3 peptide
H3K18me2 peptide
H3K18me1 peptide
H3K18me0 peptide
H3K18me3 peptide competitor
H3K18me2 peptide competitor
H3K18me1 peptide competitor
H3K18me0 peptide competitor
H3K18me1 peptide coated wells

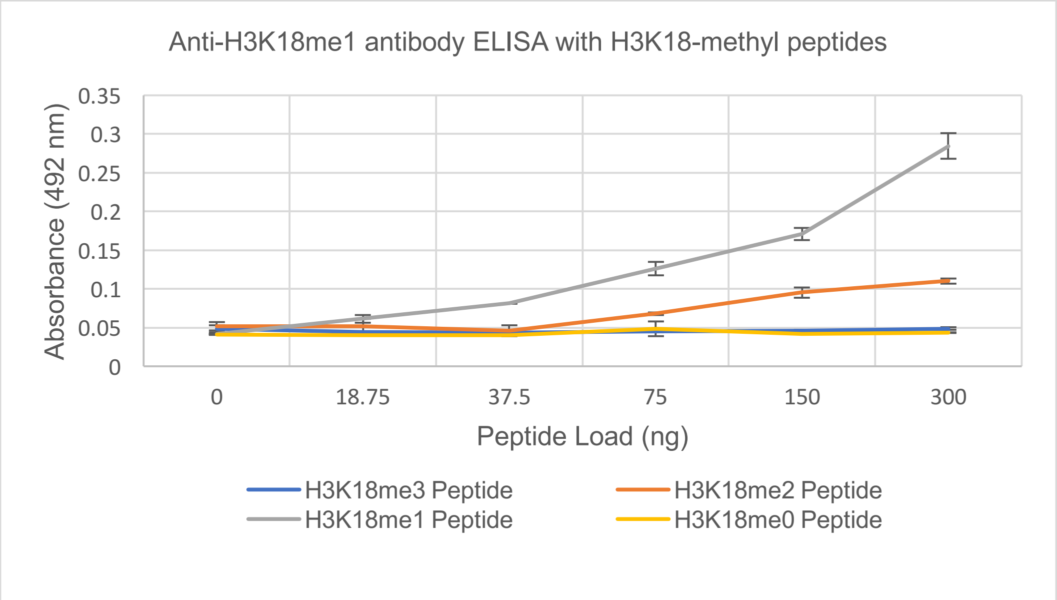
Anti-H3K18me1 antibody ELISA with H3K18-methyl peptides
Absorbance (492 nm)
0.35
0.3
0.25
0.2
0.15
0.1
0.05
0
0
18.75
37.5
75
150
300
Peptide Load (ng)
H3K18me3 Peptide
H3K18me2 Peptide
H3K18me1 Peptide
H3K18me0 Peptide

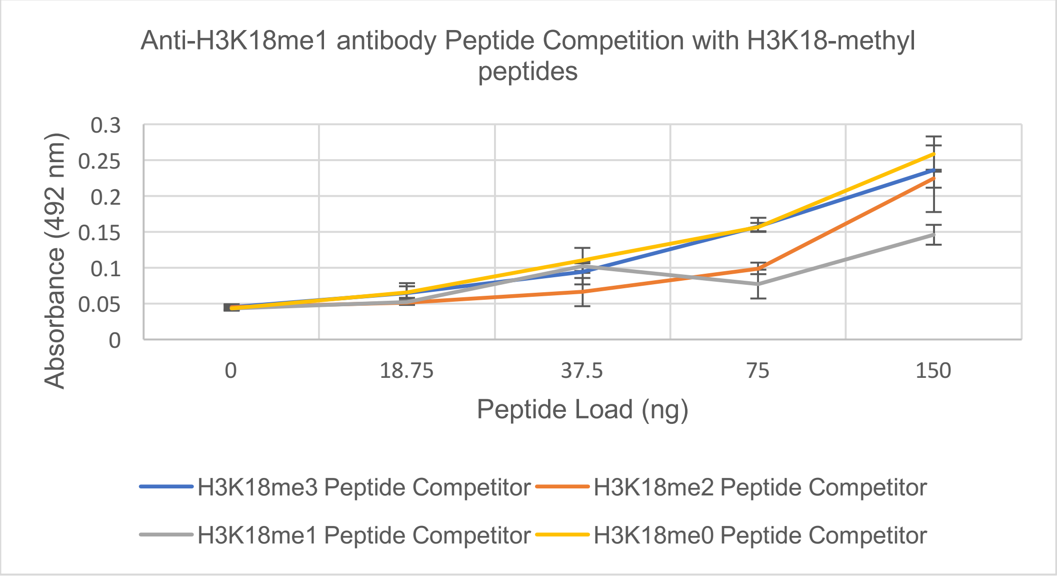
Anti-H3K18me1 antibody Peptide Competition with H3K18-methyl peptides
Absorbance (492 nm)
0.3
0.25
0.2
0.15
0.1
0.05
0
0
18.75
37.5
75
150
Peptide Load (ng)
H3K18me3 Peptide Competitor
H3K18me2 Peptide Competitor
H3K18me1 Peptide Competitor
H3K18me0 Peptide Competitor

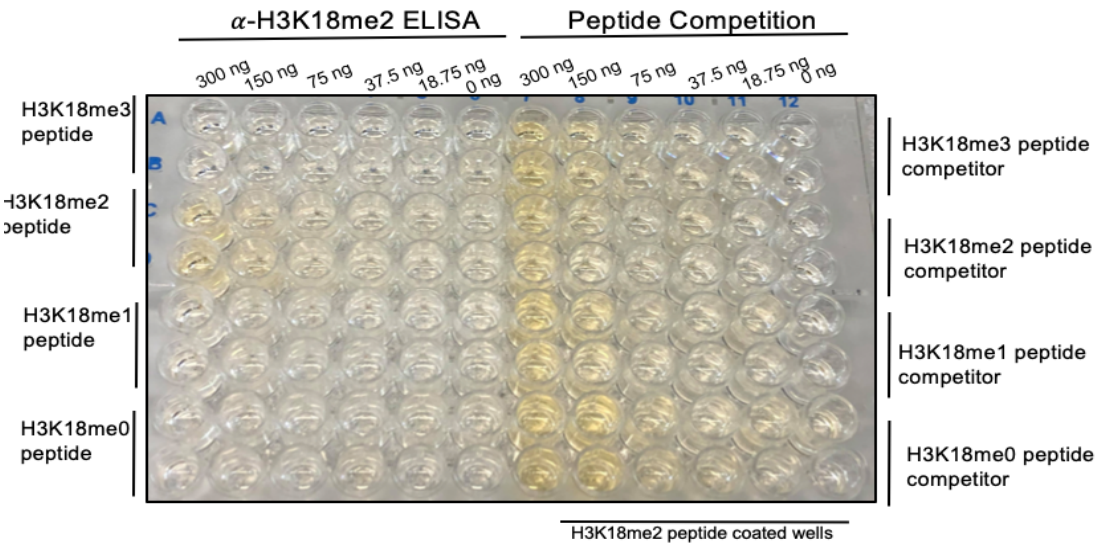

α-H3K18me2 ELISA
Peptide Competition
300 ng
150 ng
75 ng
37.5 ng
18.75 ng
0 ng
300 ng
150 ng
75 ng
37.5 ng
18.75 ng
0 ng
H3K18me3 peptide
H3K18me1 peptide
H3K18me0 peptide
H3K18me3 peptide competitor
H3K18me2 peptide competitor
H3K18me1 peptide competitor
H3K18me0 peptide competitor
H3K18me2 peptide coated wells

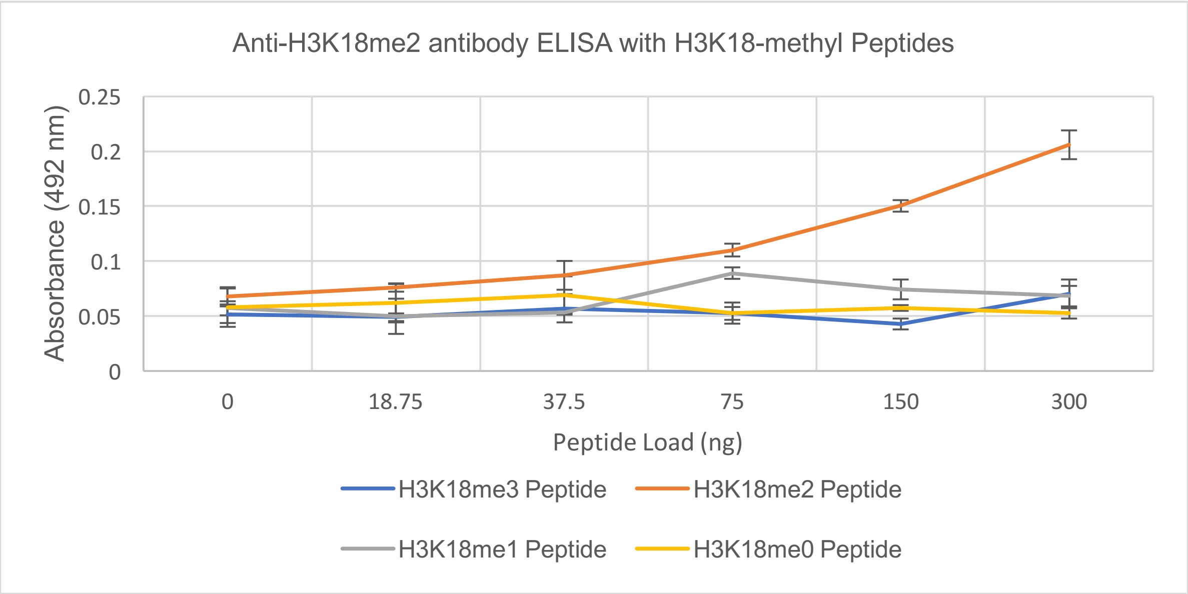

Anti-H3K18me2 antibody ELISA with H3K18-methyl Peptides
Absorbance (492 nm)
0.25
0.2
0.15
0.1
0.05
0
0
18.75
37.5
75
150
300
Peptide Load (ng)
H3K18me3 Peptide
H3K18me2 Peptide
H3K18me1 Peptide
H3K18me0 Peptide

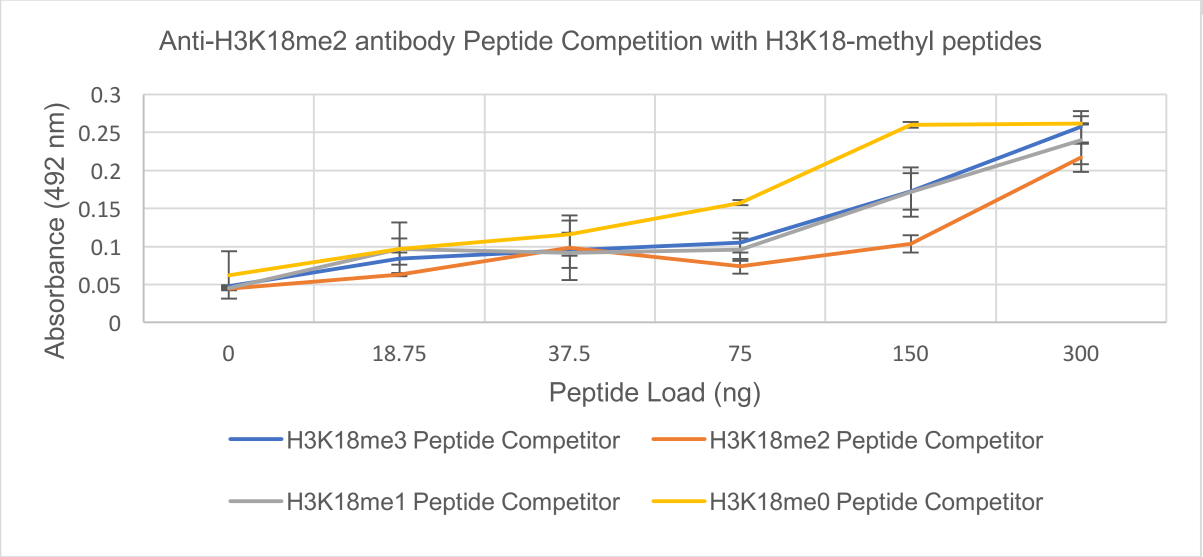

Anti-H3K18me2 antibody Peptide Competition with H3K18-methyl peptides
Absorbance (492 nm)
0.3
0.25
0.2
0.15
0.1
0.05
0
0
18.75
37.5
75
150
300
Peptide Load (ng)
H3K18me3 Peptide Competitor
H3K18me2 Peptide Competitor
H3K18me1 Peptide Competitor
H3K18me0 Peptide Competitor

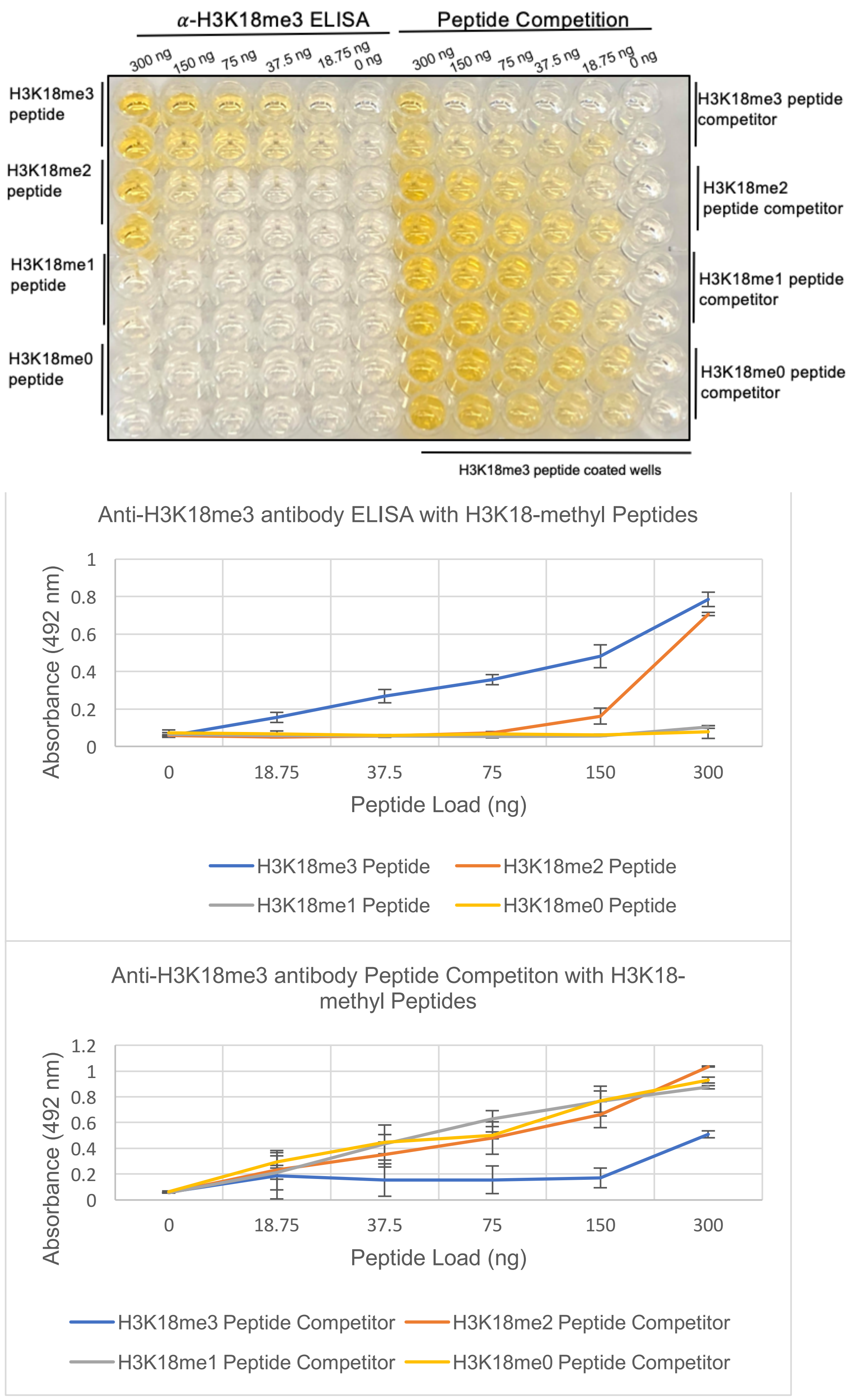
α-H3K18me3 ELISA
Peptide Competition
300 ng
150 ng
75 ng
37.5 ng
18.75 ng
0 ng
H3K18me3 peptide
H3K18me2 peptide
H3K18me1 peptide
H3K18me0 peptide
H3K18me3 peptide competitor
H3K18me2 peptide competitor
H3K18me1 peptide competitor
H3K18me0 peptide competitor
H3K18me3 peptide coated wells
Anti-H3K18me3 antibody ELISA with H3K18-methyl Peptides
Absorbance (492 nm)
Peptide Load (ng)
0
18.75
37.5
75
150
300
H3K18me3 Peptide
H3K18me2 Peptide
H3K18me1 Peptide
H3K18me0 Peptide
Anti-H3K18me3 antibody Peptide Competiton with H3K18-methyl Peptides
Absorbance (492 nm)
Peptide Load (ng)
H3K18me3 Peptide Competitor
H3K18me2 Peptide Competitor
H3K18me1 Peptide Competitor
H3K18me0 Peptide Competitor

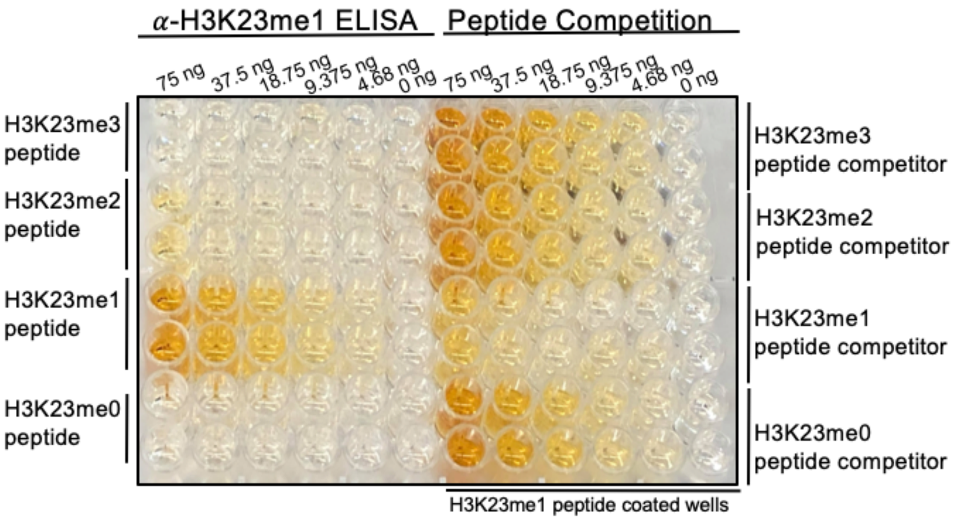
α-H3K23me1 ELISA
Peptide Competition
75 ng
37.5 ng
18.75 ng
9.375 ng
4.68 ng
0 ng
75 ng
37.5 ng
18.75 ng
9.375 ng
4.68 ng
0 ng
H3K23me3 peptide
H3K23me2 peptide
H3K23me1 peptide
H3K23me0 peptide
H3K23me3 peptide competitor
H3K23me2 peptide competitor
H3K23me1 peptide competitor
H3K23me0 peptide competitor
H3K23me1 peptide coated wells

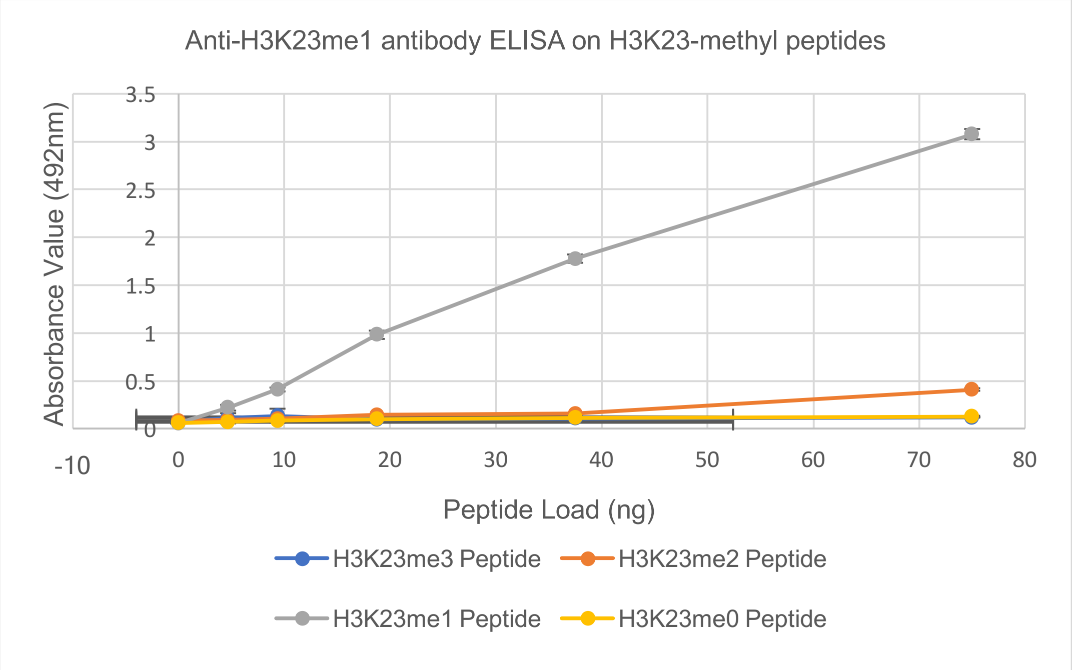
Anti-H3K23me1 antibody ELISA on H3K23-methyl peptides
Absorbance Value (492nm)
3.5
3
2.5
2
1.5
1
0.5
0
-10
0
10
20
30
40
50
60
70
80
Peptide Load (ng)
H3K23me3 Peptide
H3K23me2 Peptide
H3K23me1 Peptide
H3K23me0 Peptide

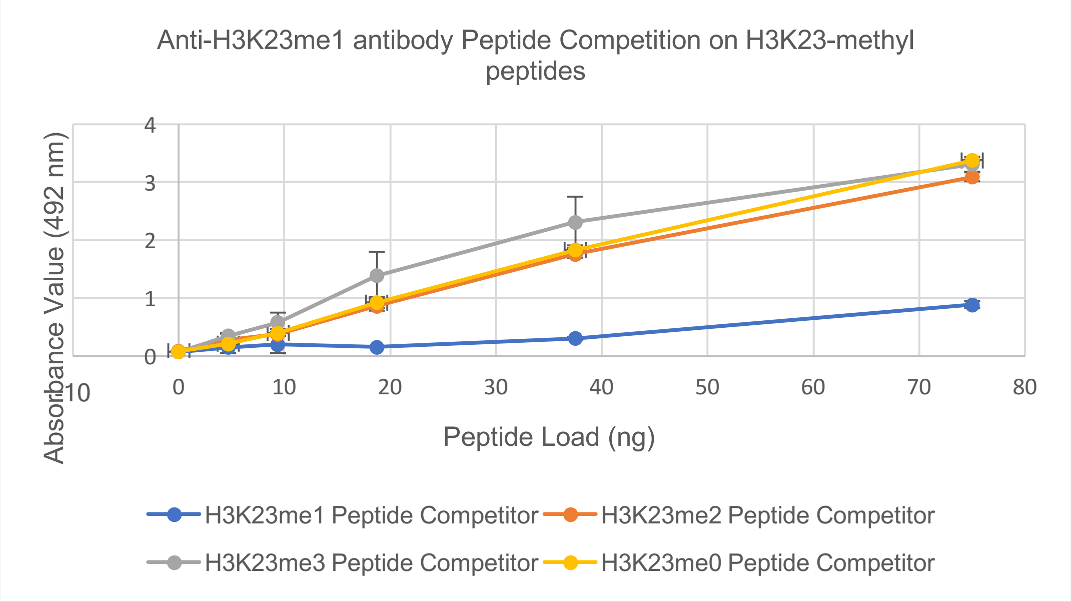
Anti-H3K23me1 antibody Peptide Competition on H3K23-methyl peptides
Absorbance Value (492 nm)
4
3
2
1
0
-10
0
10
20
30
40
50
60
70
80
Peptide Load (ng)
H3K23me1 Peptide Competitor
H3K23me2 Peptide Competitor
H3K23me3 Peptide Competitor
H3K23me0 Peptide Competitor

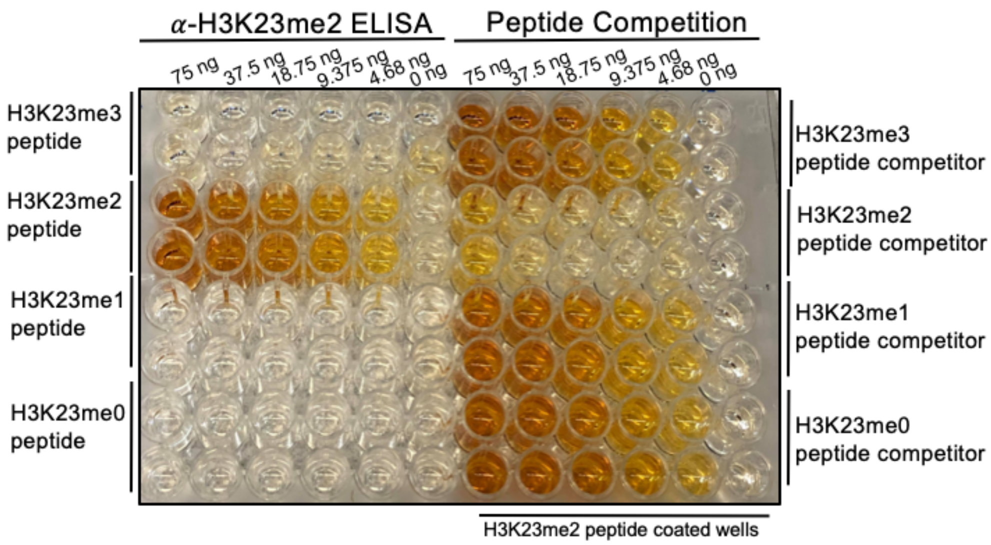
α-H3K23me2 ELISA
Peptide Competition
75 ng
37.5 ng
18.75 ng
9.375 ng
4.68 ng
0 ng
75 ng
37.5 ng
18.75 ng
9.375 ng
4.68 ng
0 ng
H3K23me3 peptide
H3K23me2 peptide
H3K23me1 peptide
H3K23me0 peptide
H3K23me3 peptide competitor
H3K23me2 peptide competitor
H3K23me1 peptide competitor
H3K23me0 peptide competitor
H3K23me2 peptide coated wells

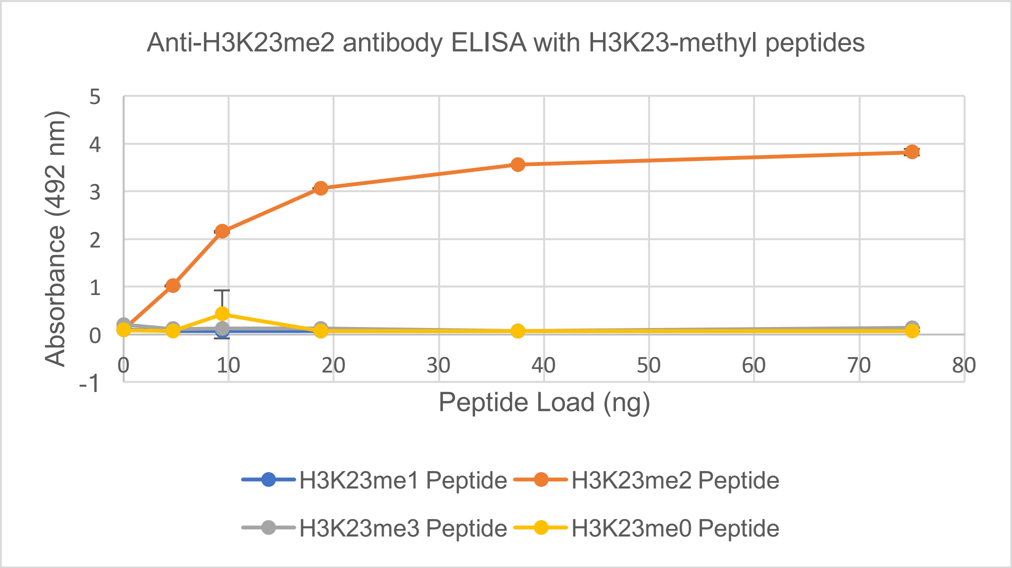
Anti-H3K23me2 antibody ELISA with H3K23-methyl peptides
Absorbance (492 nm)
5
4
3
2
1
0
-1
0
10
20
30
40
50
60
70
80
Peptide Load (ng)
H3K23me1 Peptide
H3K23me2 Peptide
H3K23me3 Peptide
H3K23me0 Peptide

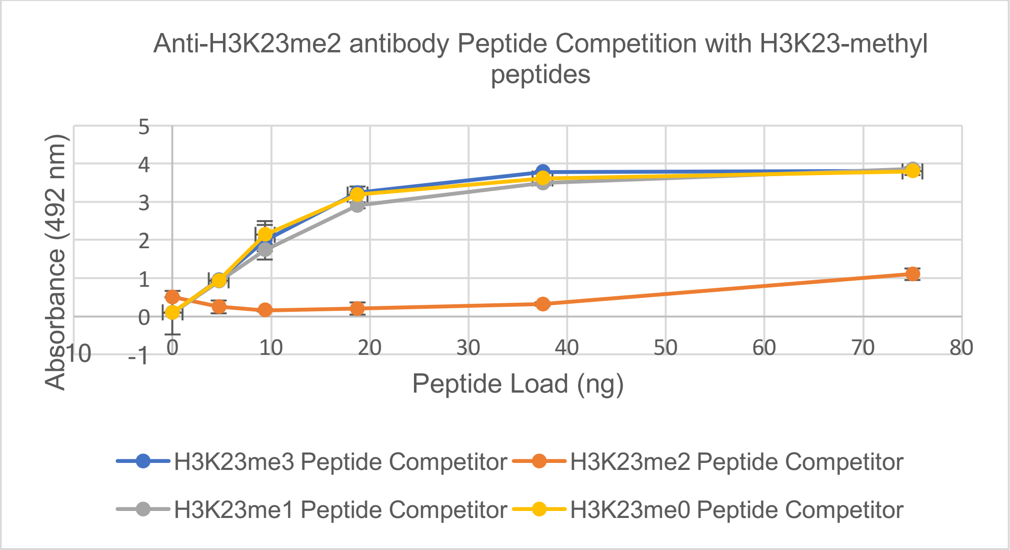
Anti-H3K23me2 antibody Peptide Competition with H3K23-methyl peptides
Absorbance (492 nm)
5
4
3
2
1
0
-1
-10
0
10
20
30
40
50
60
70
80
Peptide Load (ng)
H3K23me3 Peptide Competitor
H3K23me2 Peptide Competitor
H3K23me1 Peptide Competitor
H3K23me0 Peptide Competitor

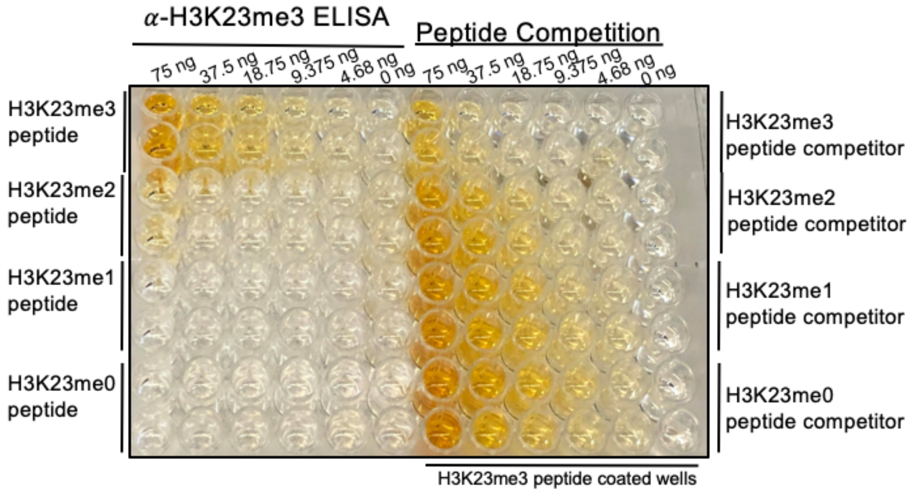

α-H3K23me3 ELISA
Peptide Competition
75 ng
37.5 ng
18.75 ng
9.375 ng
4.68 ng
0 ng
75 ng
37.5 ng
18.75 ng
9.375 ng
4.68 ng
0 ng
H3K23me3 peptide
H3K23me2 peptide
H3K23me1 peptide
H3K23me0 peptide
H3K23me3 peptide competitor
H3K23me2 peptide competitor
H3K23me1 peptide competitor
H3K23me0 peptide competitor
H3K23me3 peptide coated wells

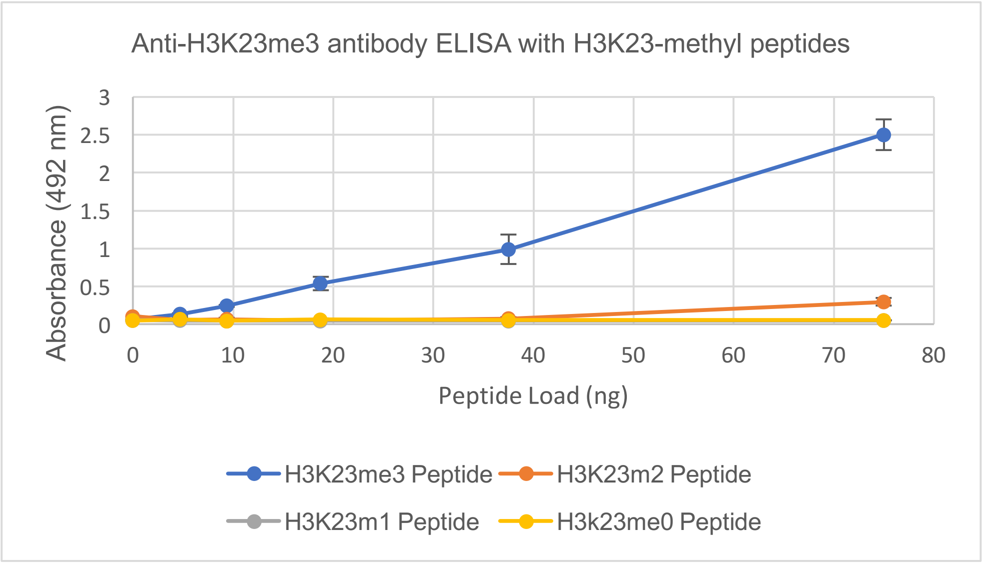

Anti-H3K23me3 antibody ELISA with H3K23-methyl peptides
Absorbance (492 nm)
3
2.5
2
1.5
1
0.5
0
0
10
20
30
40
50
60
70
80
Peptide Load (ng)
H3K23me3 Peptide
H3K23m2 Peptide
H3K23m1 Peptide
H3k23me0 Peptide

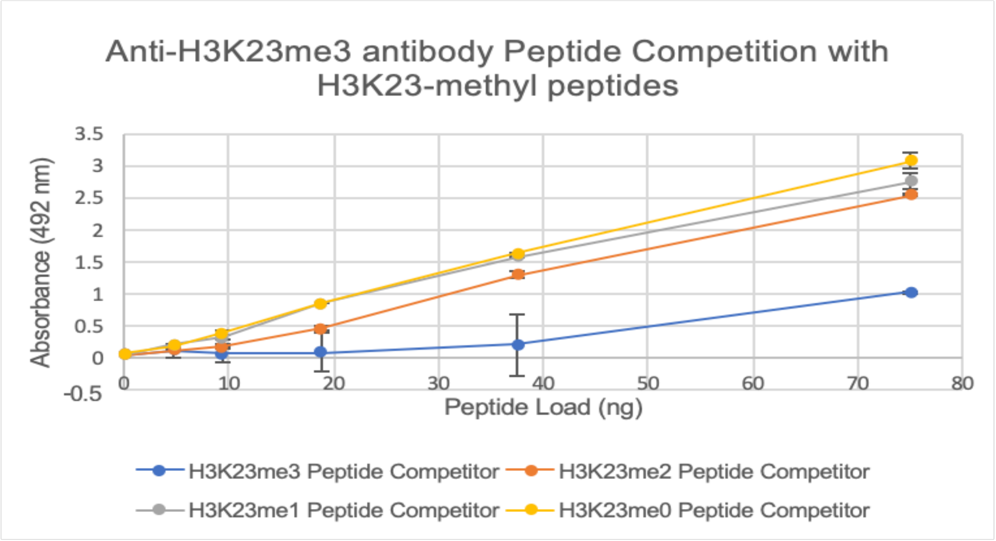

Anti-H3K23me3 antibody Peptide Competition with H3K23-methyl peptides
Absorbance (492 nm)
3.5
3
2.5
2
1.5
1
0.5
0
-0.5
0
10
20
30
40
50
60
70
80
Peptide Load (ng)
H3K23me3 Peptide Competitor
H3K23me2 Peptide Competitor
H3K23me1 Peptide Competitor
H3K23me0 Peptide Competitor

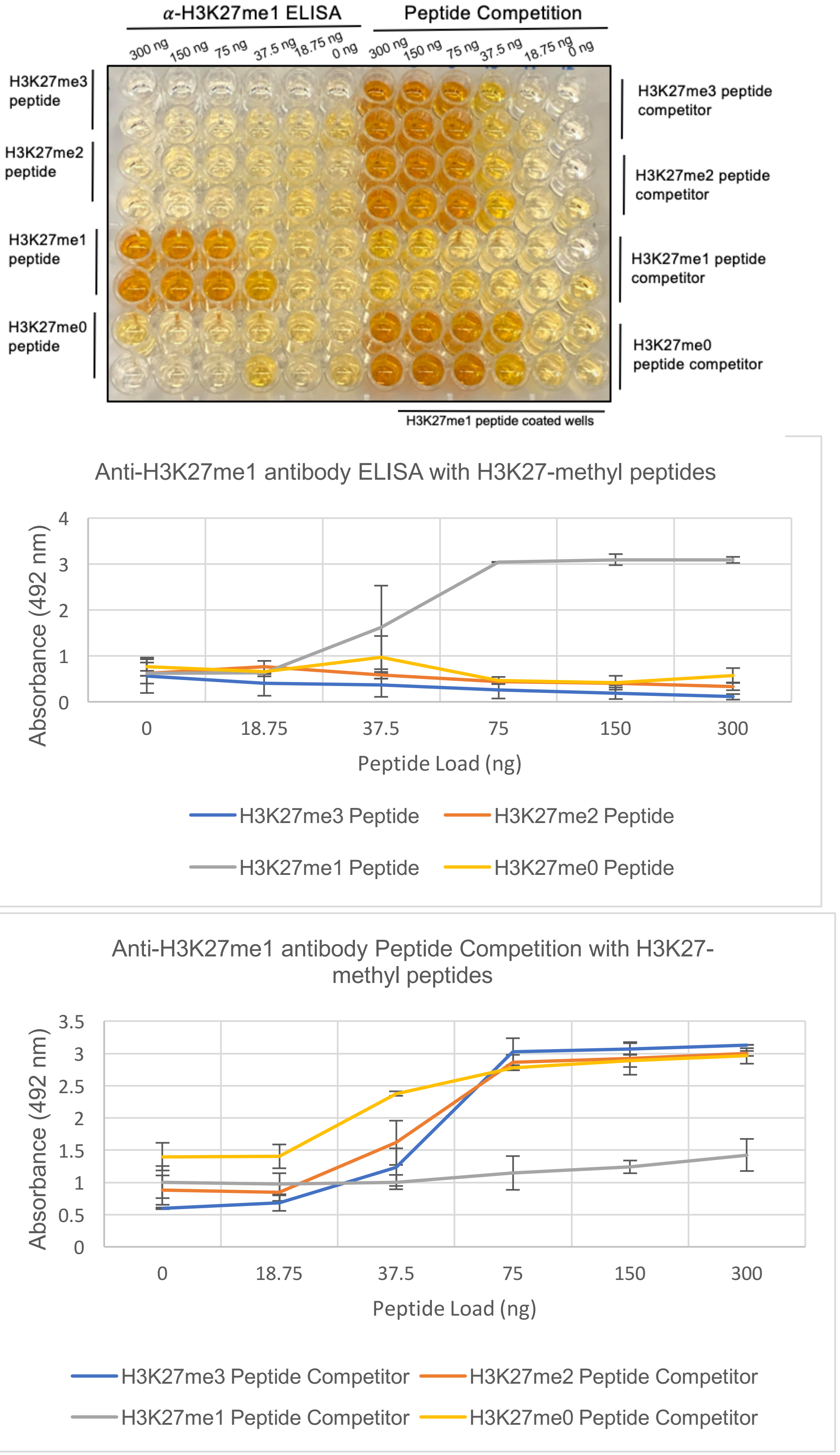
α-H3K27me1 ELISA
Peptide Competition
300 ng
150 ng
75 ng
37.5 ng
18.75 ng
0 ng
300 ng
150 ng
75 ng
37.5 ng
18.75 ng
0 ng
H3K27me3 peptide
H3K27me2 peptide
H3K27me1 peptide
H3K27me0 peptide
H3K27me3 peptide competitor
H3K27me2 peptide competitor
H3K27me1 peptide competitor
H3K27me0 peptide competitor
H3K27me1 peptide coated wells
Anti-H3K27me1 antibody ELISA with H3K27-methyl peptides
Absorbance (492 nm)
4
3
2
1
0
0
18.75
37.5
75
150
300
Peptide Load (ng)
H3K27me3 Peptide
H3K27me2 Peptide
H3K27me1 Peptide
H3K27me0 Peptide
Anti-H3K27me1 antibody Peptide Competition with H3K27-methyl peptides
Absorbance (492 nm)
3.5
3
2.5
2
1.5
1
0.5
0
0
18.75
37.5
75
150
300
Peptide Load (ng)
H3K27me3 Peptide Competitor
H3K27me2 Peptide Competitor
H3K27me1 Peptide Competitor
H3K27me0 Peptide Competitor

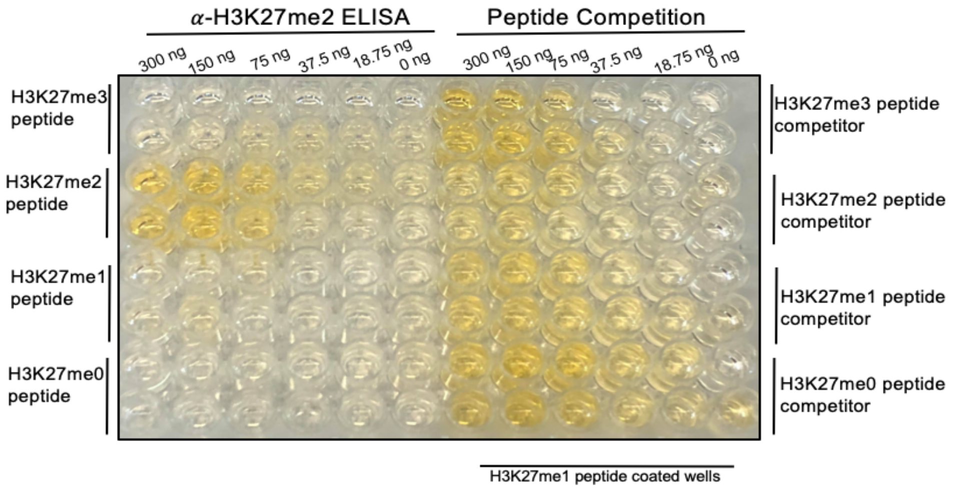
α-H3K27me2 ELISA
Peptide Competition
300 ng
150 ng
75 ng
37.5 ng
18.75 ng
0 ng
300 ng
150 ng
75 ng
37.5 ng
18.75 ng
0 ng
H3K27me3 peptide
H3K27me2 peptide
H3K27me1 peptide
H3K27me0 peptide
H3K27me3 peptide competitor
H3K27me2 peptide competitor
H3K27me1 peptide competitor
H3K27me0 peptide competitor
H3K27me1 peptide coated wells

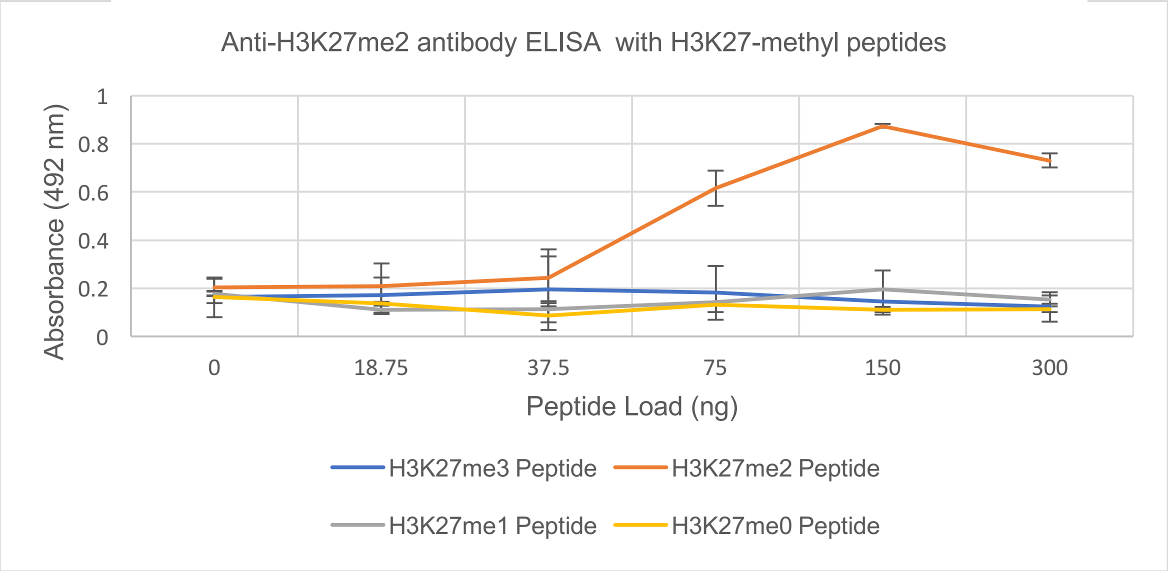
Anti-H3K27me2 antibody ELISA with H3K27-methyl peptides
Absorbance (492 nm)
1
0.8
0.6
0.4
0.2
0
0
18.75
37.5
75
150
300
Peptide Load (ng)
H3K27me3 Peptide
H3K27me2 Peptide
H3K27me1 Peptide
H3K27me0 Peptide

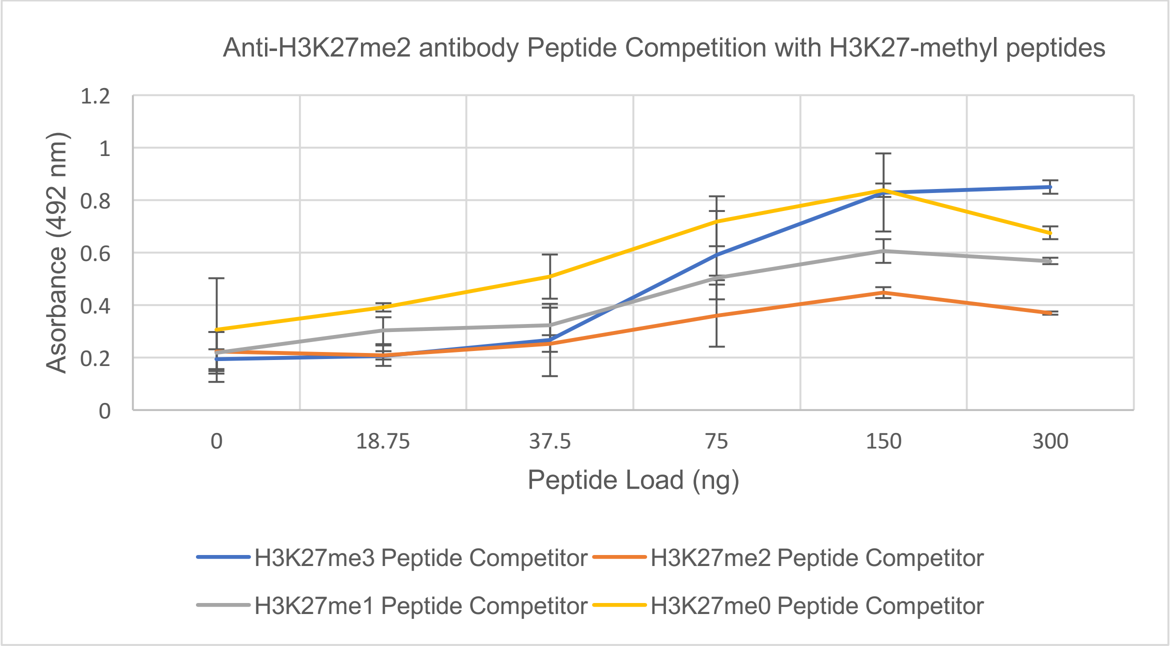
Anti-H3K27me2 antibody Peptide Competition with H3K27-methyl peptides
Asorbance (492 nm)
1.2
1
0.8
0.6
0.4
0.2
0
0
18.75
37.5
75
150
300
Peptide Load (ng)
H3K27me3 Peptide Competitor
H3K27me2 Peptide Competitor
H3K27me1 Peptide Competitor
H3K27me0 Peptide Competitor

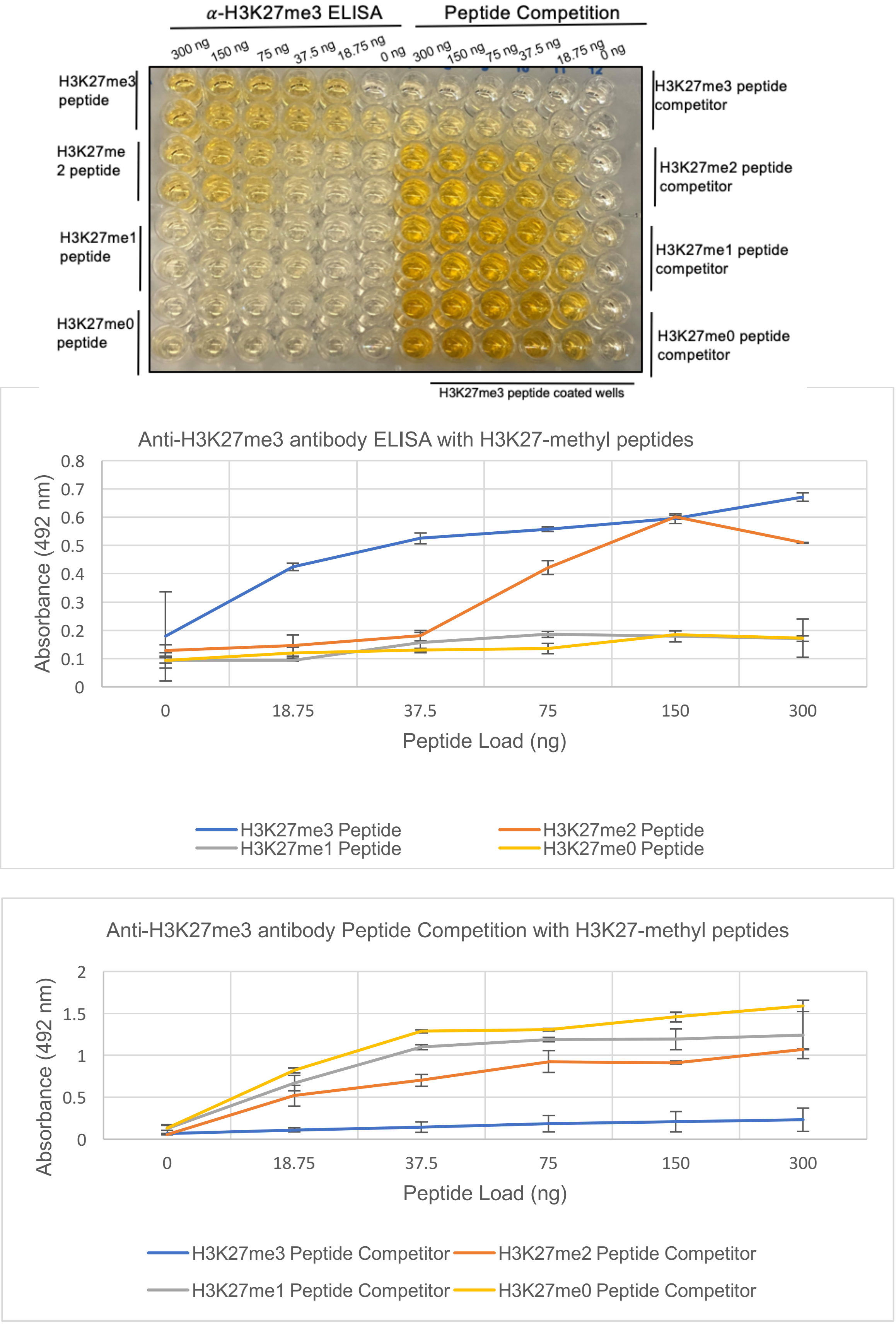

α-H3K27me3 ELISA
Peptide Competition
300 ng
150 ng
75 ng
37.5 ng
18.75 ng
0 ng
H3K27me3 peptide
H3K27me2 peptide
H3K27me1 peptide
H3K27me0 peptide
H3K27me3 peptide competitor
H3K27me2 peptide competitor
H3K27me1 peptide competitor
H3K27me0 peptide competitor
H3K27me3 peptide coated wells
Anti-H3K27me3 antibody ELISA with H3K27-methyl peptides
Absorbance (492 nm)
Peptide Load (ng)
0
18.75
37.5
75
150
300
H3K27me3 Peptide
H3K27me2 Peptide
H3K27me1 Peptide
H3K27me0 Peptide
Anti-H3K27me3 antibody Peptide Competition with H3K27-methyl peptides
H3K27me3 Peptide Competitor
H3K27me2 Peptide Competitor
H3K27me1 Peptide Competitor
H3K27me0 Peptide Competitor

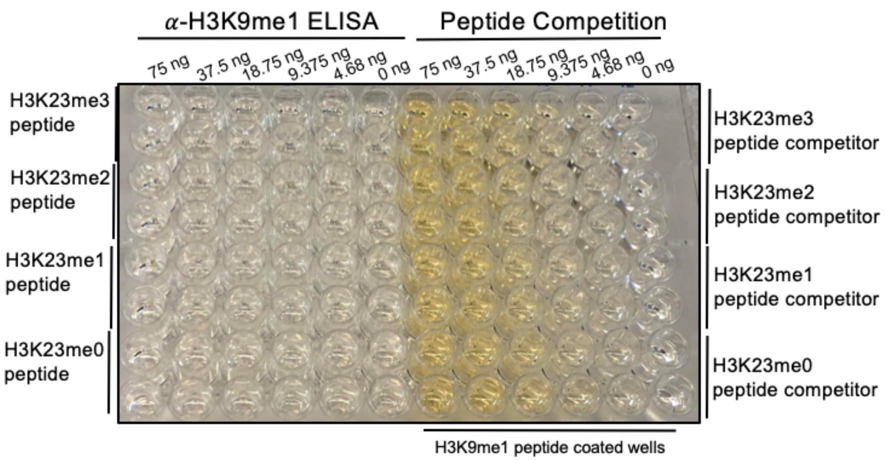

α-H3K9me1 ELISA
Peptide Competition
75 ng
37.5 ng
18.75 ng
9.375 ng
4.68 ng
0 ng
75 ng
37.5 ng
18.75 ng
9.375 ng
4.68 ng
0 ng
H3K23me3 peptide
H3K23me2 peptide
H3K23me1 peptide
H3K23me0 peptide
H3K23me3 peptide competitor
H3K23me2 peptide competitor
H3K23me1 peptide competitor
H3K23me0 peptide competitor
H3K9me1 peptide coated wells

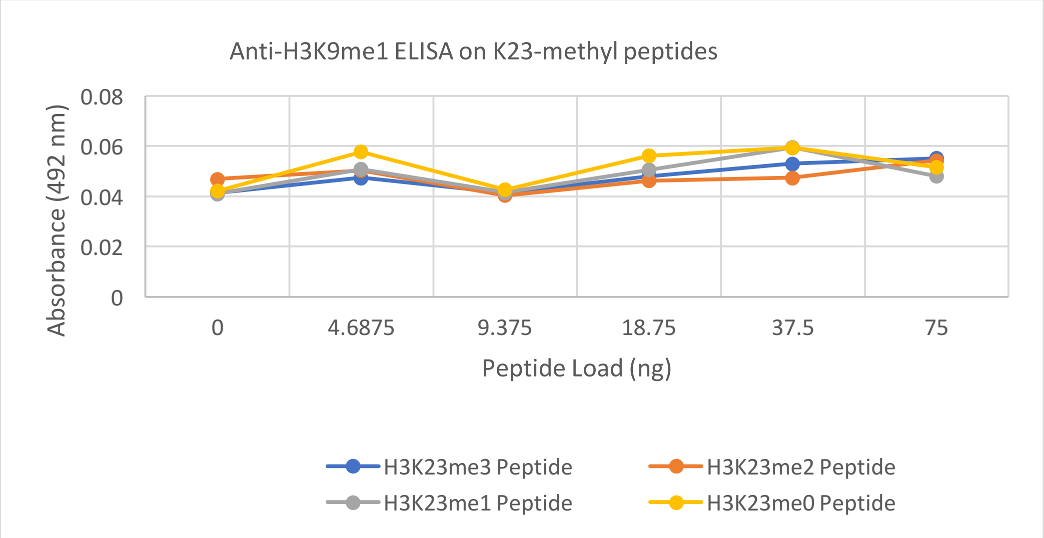

Anti-H3K9me1 ELISA on K23-methyl peptides
Absorbance (492 nm)
0.08
0.06
0.04
0.02
0
0
4.6875
9.375
18.75
37.5
75
Peptide Load (ng)
H3K23me3 Peptide
H3K23me2 Peptide
H3K23me1 Peptide
H3K23me0 Peptide

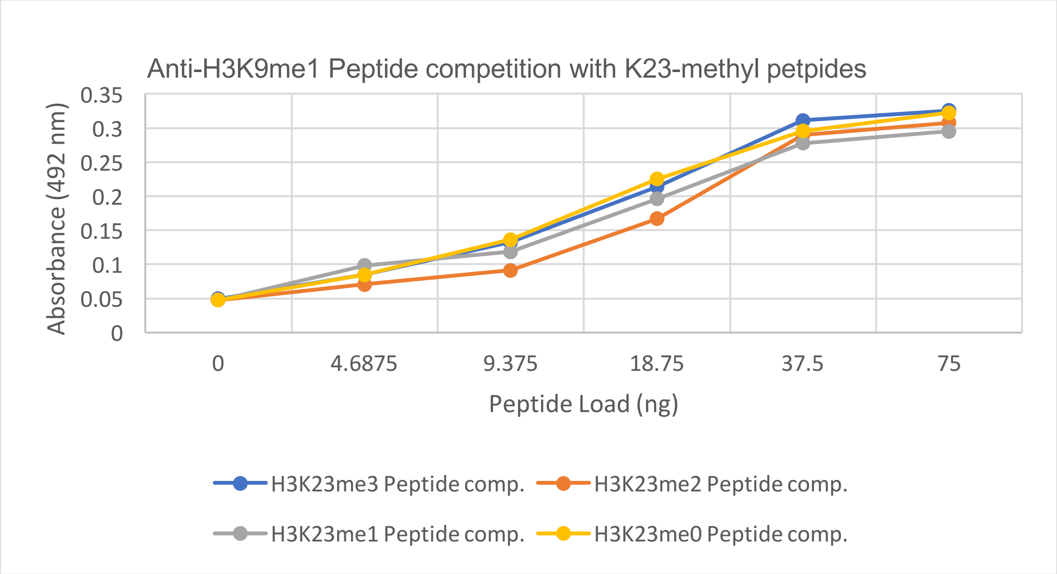

Anti-H3K9me1 Peptide competition with K23-methyl petpides
Absorbance (492 nm)
0.35
0.3
0.25
0.2
0.15
0.1
0.05
0
0
4.6875
9.375
18.75
37.5
75
Peptide Load (ng)
H3K23me3 Peptide comp.
H3K23me2 Peptide comp.
H3K23me1 Peptide comp.
H3K23me0 Peptide comp.

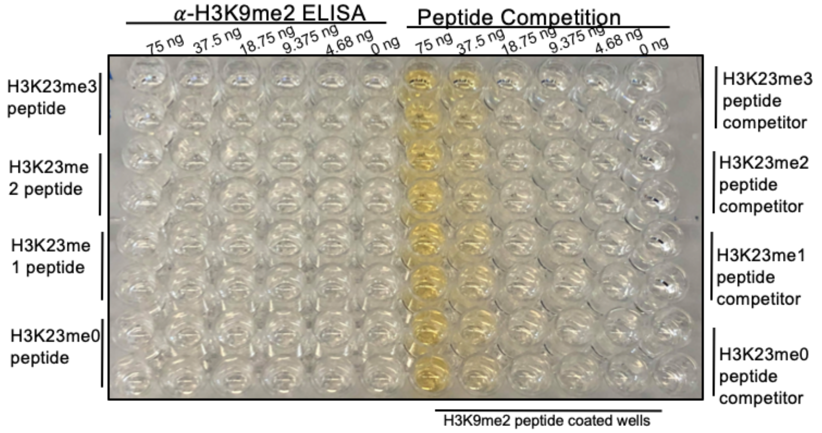
α-H3K9me2 ELISA
Peptide Competition
75 ng
37.5 ng
18.75 ng
9.375 ng
4.68 ng
0 ng
H3K23me3 peptide
H3K23me 2 peptide
H3K23me 1 peptide
H3K23me0 peptide
H3K23me3 peptide competitor
H3K23me2 peptide competitor
H3K23me1 peptide competitor
H3K23me0 peptide competitor
H3K9me2 peptide coated wells

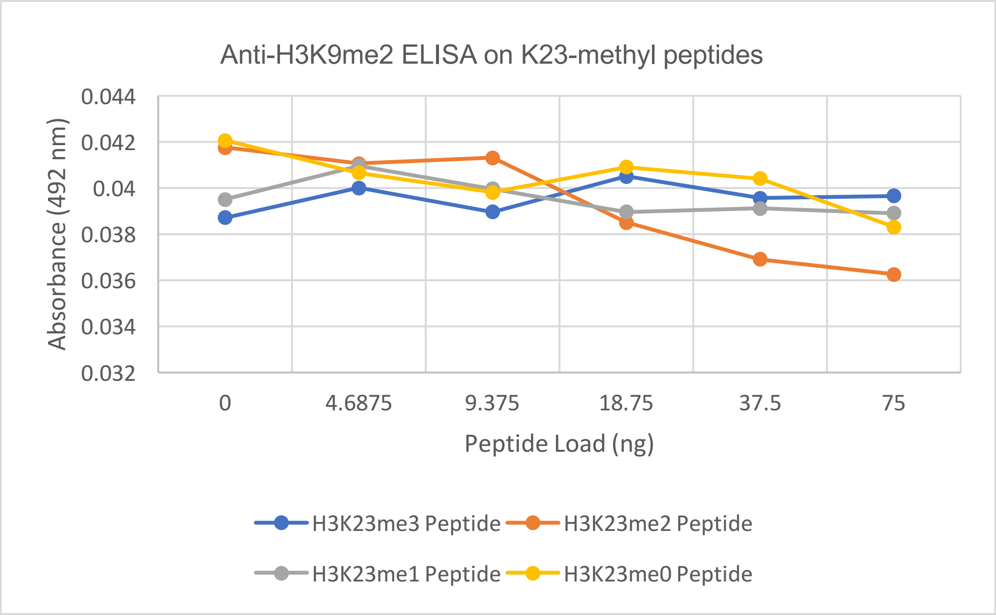
Anti-H3K9me2 ELISA on K23-methyl peptides
Absorbance (492 nm)
0.044
0.042
0.04
0.038
0.036
0.034
0.032
0
4.6875
9.375
18.75
37.5
75
Peptide Load (ng)
H3K23me3 Peptide
H3K23me2 Peptide
H3K23me1 Peptide
H3K23me0 Peptide

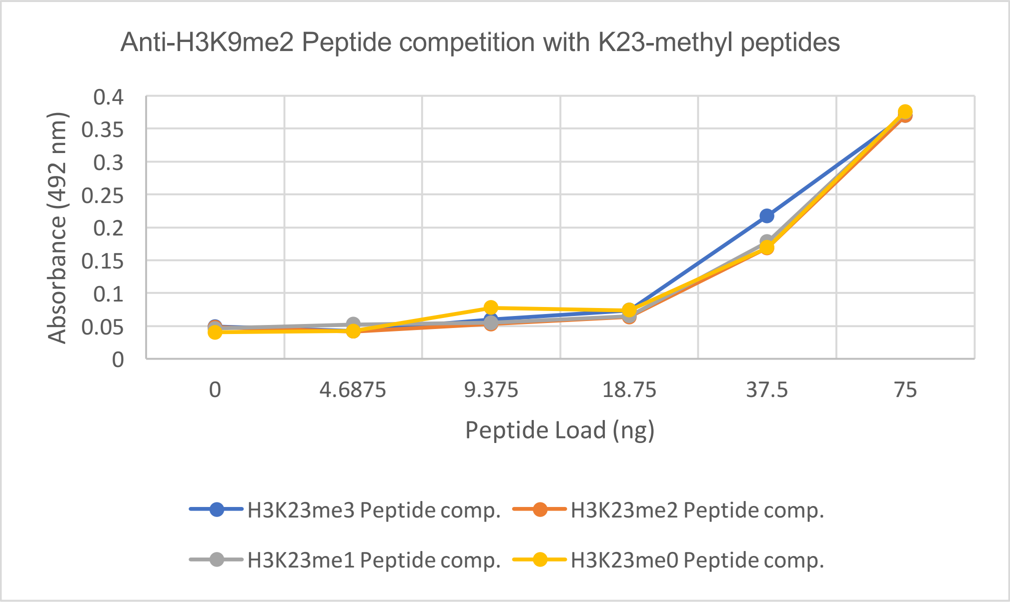
Anti-H3K9me2 Peptide competition with K23-methyl peptides
Absorbance (492 nm)
0.4
0.35
0.3
0.25
0.2
0.15
0.1
0.05
0
0
4.6875
9.375
18.75
37.5
75
Peptide Load (ng)
H3K23me3 Peptide comp.
H3K23me2 Peptide comp.
H3K23me1 Peptide comp.
H3K23me0 Peptide comp.

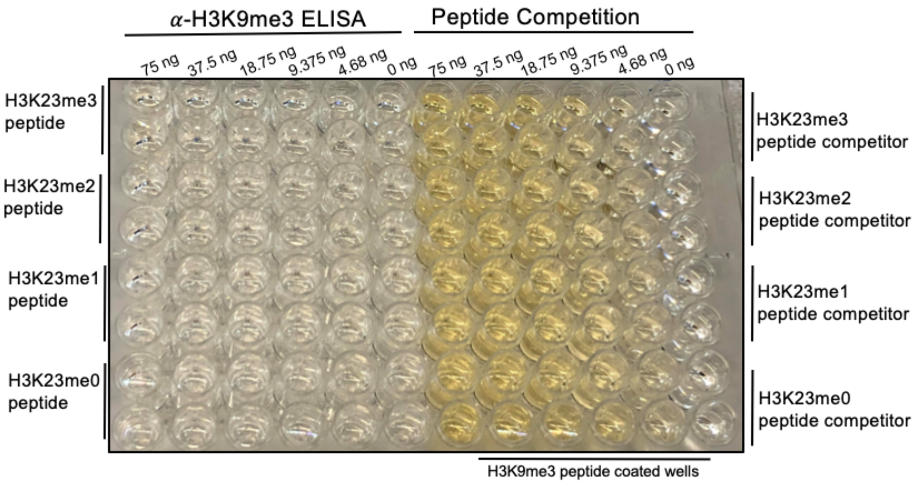

α-H3K9me3 ELISA
Peptide Competition
75 ng
37.5 ng
18.75 ng
9.375 ng
4.68 ng
0 ng
75 ng
37.5 ng
18.75 ng
9.375 ng
4.68 ng
0 ng
H3K23me3 peptide
H3K23me2 peptide
H3K23me1 peptide
H3K23me0 peptide
H3K23me3 peptide competitor
H3K23me2 peptide competitor
H3K23me1 peptide competitor
H3K23me0 peptide competitor
H3K9me3 peptide coated wells

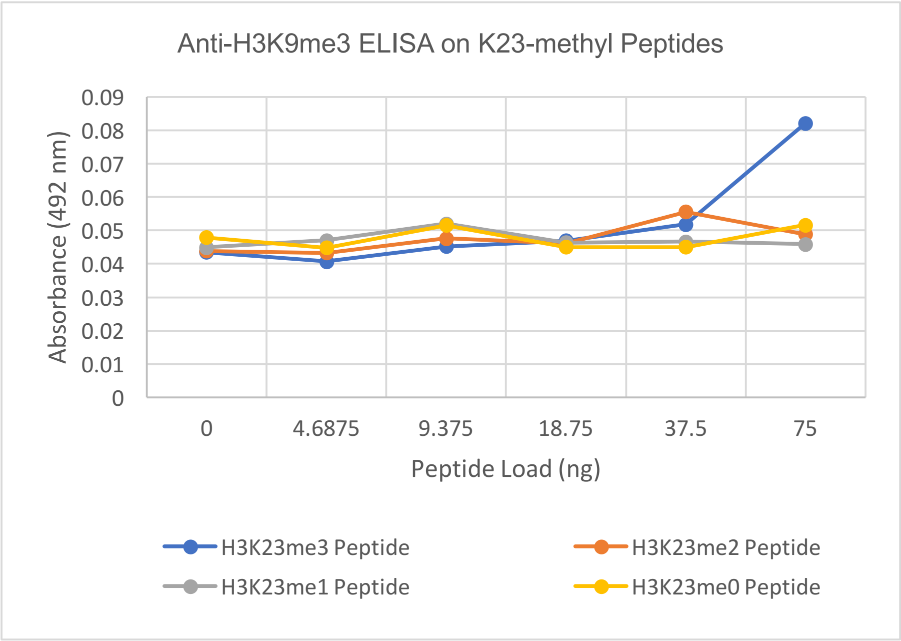

Anti-H3K9me3 ELISA on K23-methyl Peptides
Absorbance (492 nm)
0.09
0.08
0.07
0.06
0.05
0.04
0.03
0.02
0.01
0
0
4.6875
9.375
18.75
37.5
75
Peptide Load (ng)
H3K23me3 Peptide
H3K23me2 Peptide
H3K23me1 Peptide
H3K23me0 Peptide

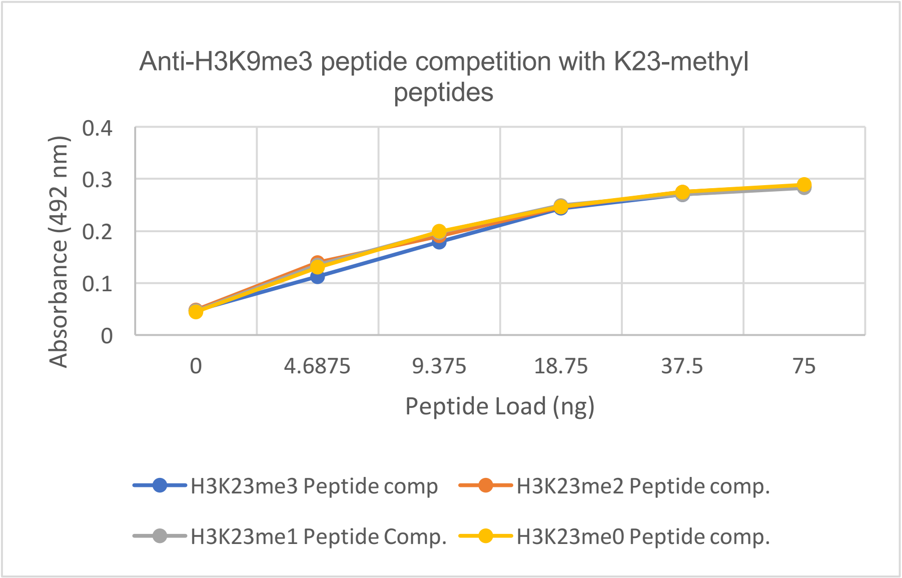

Anti-H3K9me3 peptide competition with K23-methyl peptides
Absorbance (492 nm)
0.4
0.3
0.2
0.1
0
0
4.6875
9.375
18.75
37.5
75
Peptide Load (ng)
H3K23me3 Peptide comp
H3K23me2 Peptide comp.
H3K23me1 Peptide Comp.
H3K23me0 Peptide comp.

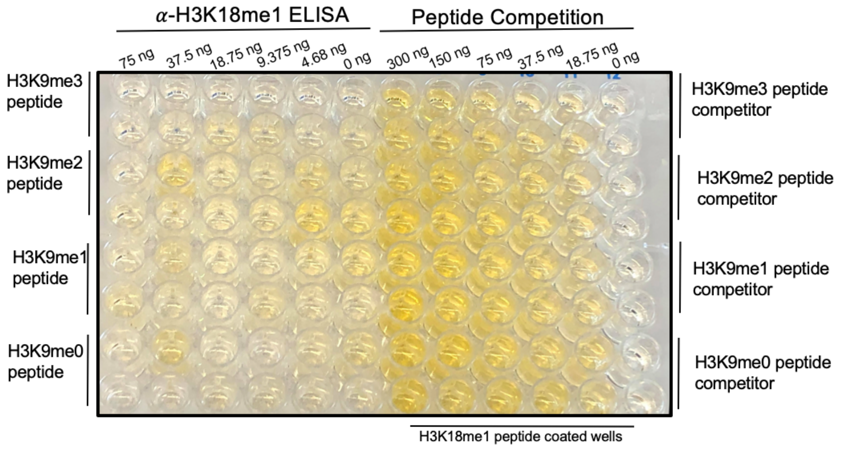
α-H3K18me1 ELISA
Peptide Competition
75 ng
37.5 ng
18.75 ng
9.375 ng
4.68 ng
0 ng
300 ng
150 ng
75 ng
37.5 ng
18.75 ng
0 ng
H3K9me3 peptide
H3K9me2 peptide
H3K9me1 peptide
H3K9me0 peptide
H3K9me3 peptide competitor
H3K9me2 peptide competitor
H3K9me1 peptide competitor
H3K9me0 peptide competitor
H3K18me1 peptide coated wells

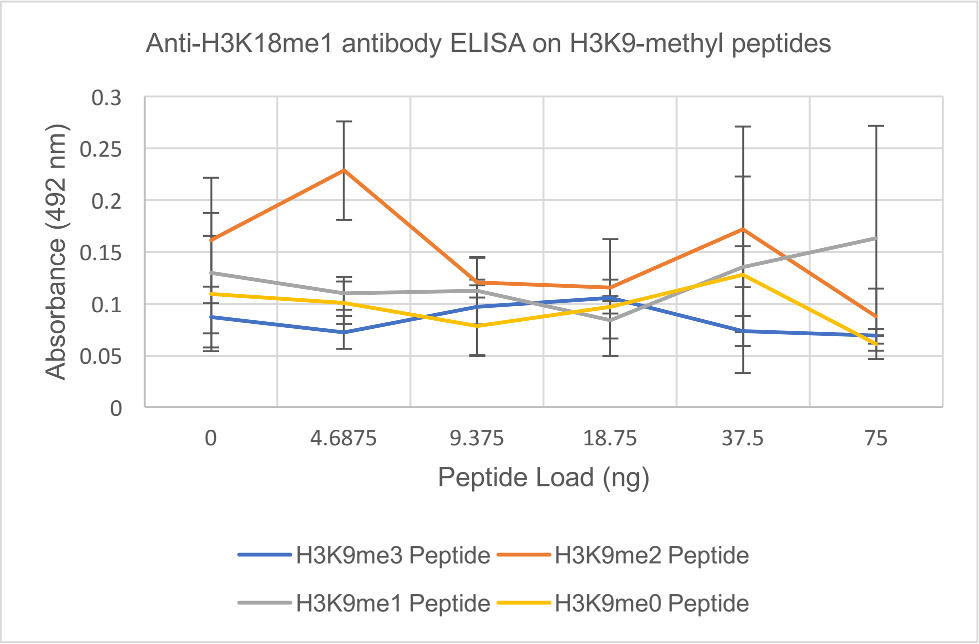
Anti-H3K18me1 antibody ELISA on H3K9-methyl peptides
Absorbance (492 nm)
0.3
0.25
0.2
0.15
0.1
0.05
0
0
4.6875
9.375
18.75
37.5
75
Peptide Load (ng)
H3K9me3 Peptide
H3K9me2 Peptide
H3K9me1 Peptide
H3K9me0 Peptide

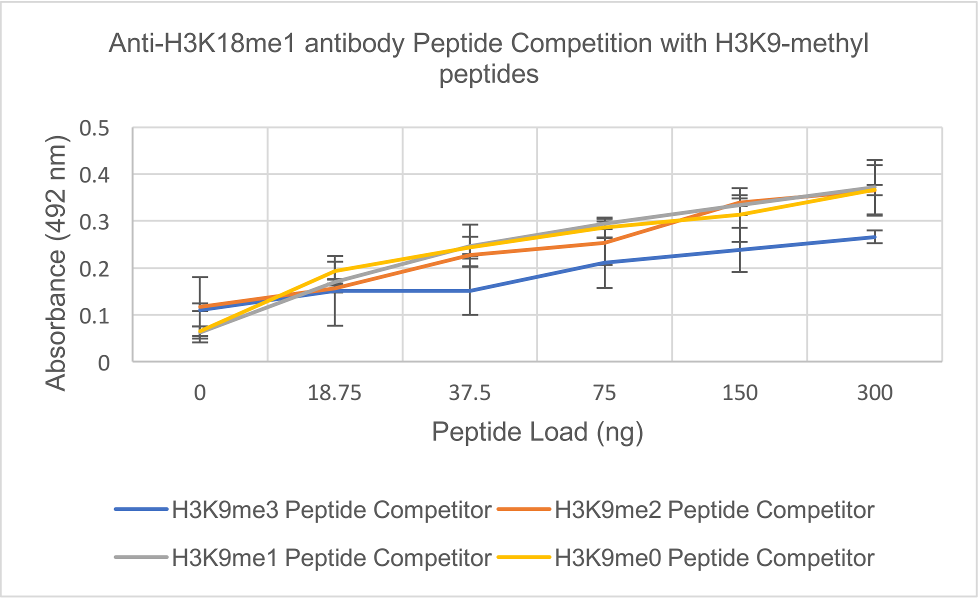
Anti-H3K18me1 antibody Peptide Competition with H3K9-methyl peptides
Absorbance (492 nm)
0.5
0.4
0.3
0.2
0.1
0
0
18.75
37.5
75
150
300
Peptide Load (ng)
H3K9me3 Peptide Competitor
H3K9me2 Peptide Competitor
H3K9me1 Peptide Competitor
H3K9me0 Peptide Competitor

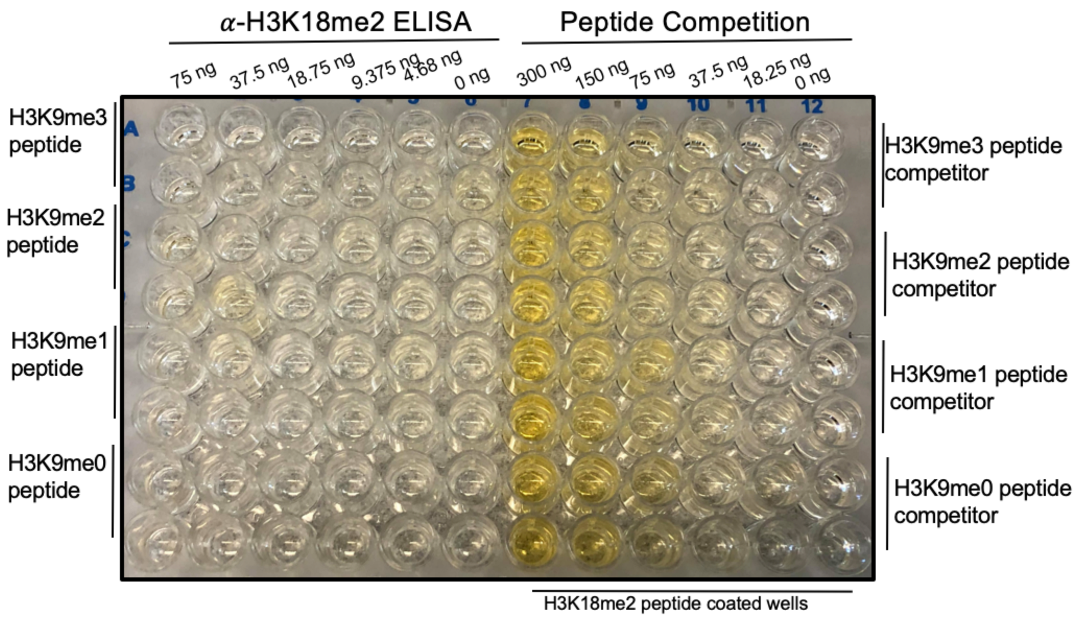
α-H3K18me2 ELISA
Peptide Competition
75 ng
37.5 ng
18.75 ng
9.375 ng
4.68 ng
0 ng
300 ng
150 ng
75 ng
37.5 ng
18.25 ng
0 ng
H3K9me3 peptide
H3K9me2 peptide
H3K9me1 peptide
H3K9me0 peptide
H3K9me3 peptide competitor
H3K9me2 peptide competitor
H3K9me1 peptide competitor
H3K9me0 peptide competitor
H3K18me2 peptide coated wells

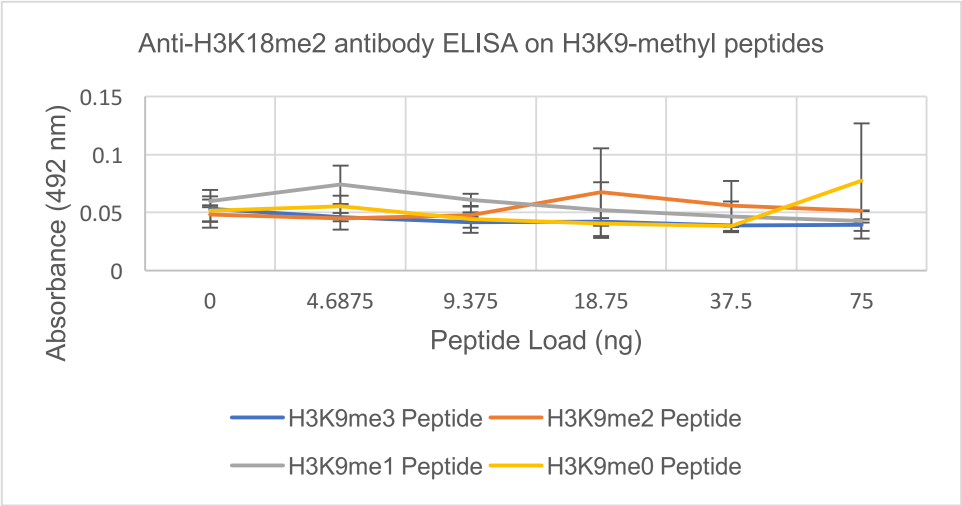
Anti-H3K18me2 antibody ELISA on H3K9-methyl peptides
Absorbance (492 nm)
0.15
0.1
0.05
0
0
4.6875
9.375
18.75
37.5
75
Peptide Load (ng)
H3K9me3 Peptide
H3K9me2 Peptide
H3K9me1 Peptide
H3K9me0 Peptide

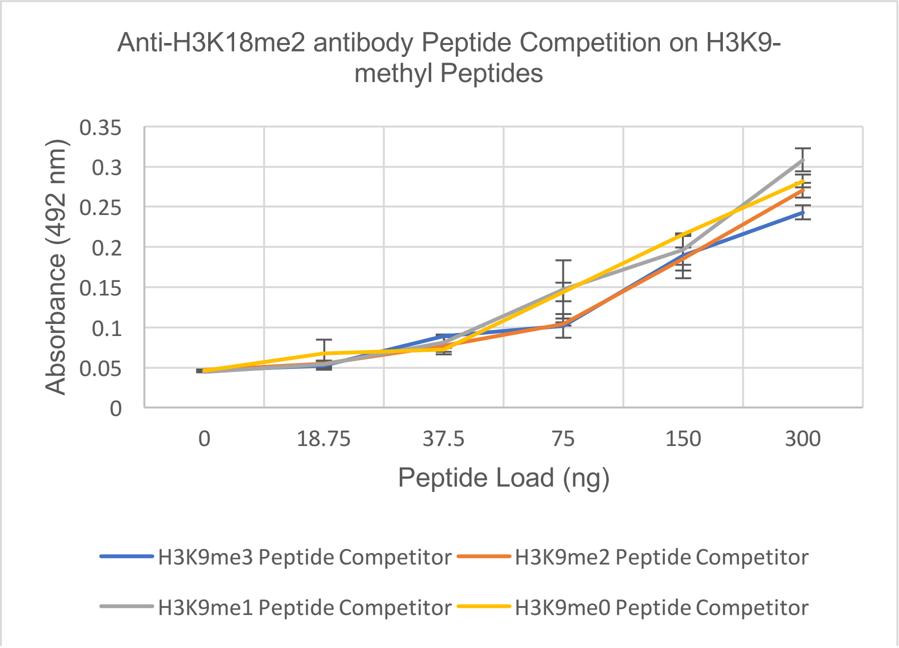
Anti-H3K18me2 antibody Peptide Competition on H3K9-methyl Peptides
Absorbance (492 nm)
0.35
0.3
0.25
0.2
0.15
0.1
0.05
0
0
18.75
37.5
75
150
300
Peptide Load (ng)
H3K9me3 Peptide Competitor
H3K9me2 Peptide Competitor
H3K9me1 Peptide Competitor
H3K9me0 Peptide Competitor

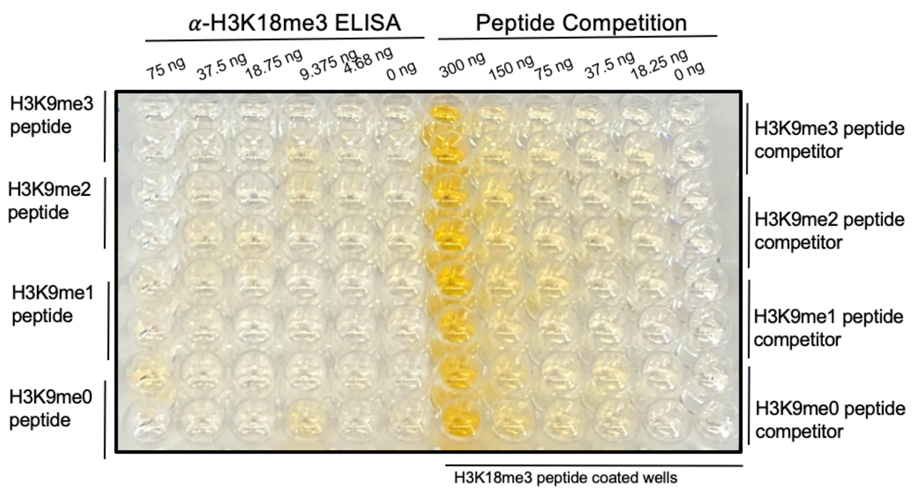
α-H3K18me3 ELISA
Peptide Competition
75 ng
37.5 ng
18.75 ng
9.375 ng
4.68 ng
0 ng
300 ng
150 ng
75 ng
37.5 ng
18.25 ng
0 ng
H3K9me3 peptide
H3K9me2 peptide
H3K9me1 peptide
H3K9me0 peptide
H3K9me3 peptide competitor
H3K9me2 peptide competitor
H3K9me1 peptide competitor
H3K9me0 peptide competitor
H3K18me3 peptide coated wells

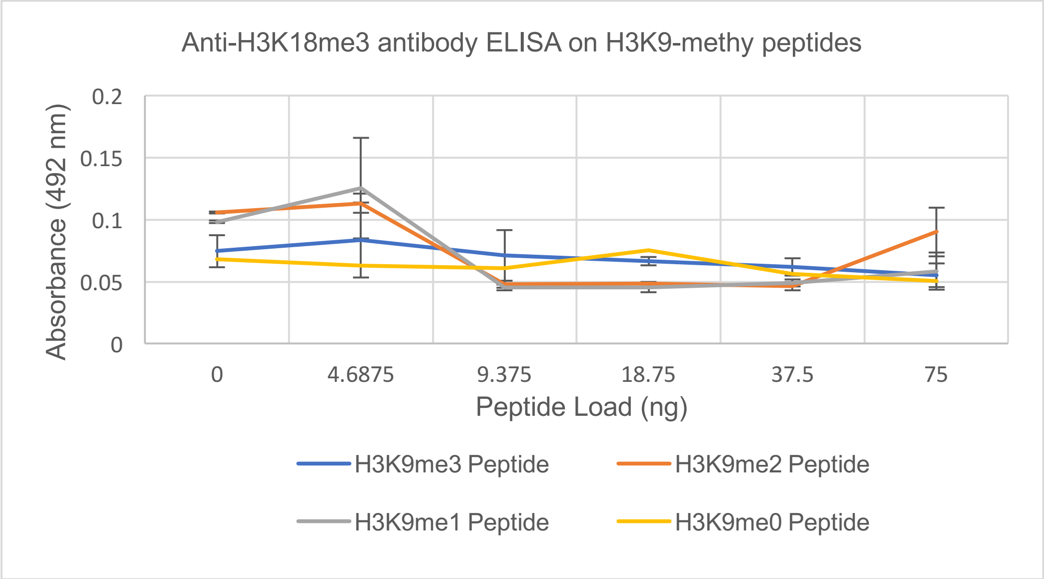
Anti-H3K18me3 antibody ELISA on H3K9-methy peptides
Absorbance (492 nm)
0.2
0.15
0.1
0.05
0
0
4.6875
9.375
18.75
37.5
75
Peptide Load (ng)
H3K9me3 Peptide
H3K9me2 Peptide
H3K9me1 Peptide
H3K9me0 Peptide

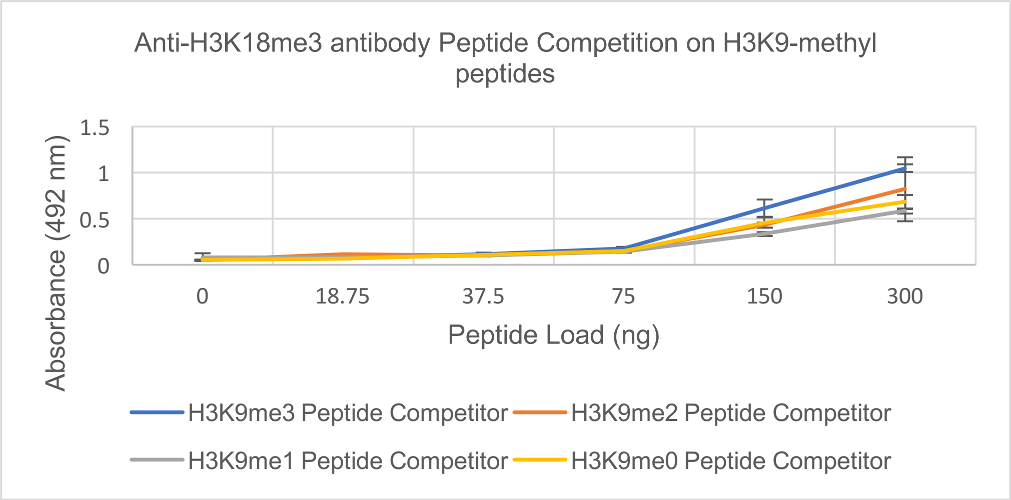
Anti-H3K18me3 antibody Peptide Competition on H3K9-methyl peptides
Absorbance (492 nm)
1.5
1
0.5
0
0
18.75
37.5
75
150
300
Peptide Load (ng)
H3K9me3 Peptide Competitor
H3K9me2 Peptide Competitor
H3K9me1 Peptide Competitor
H3K9me0 Peptide Competitor

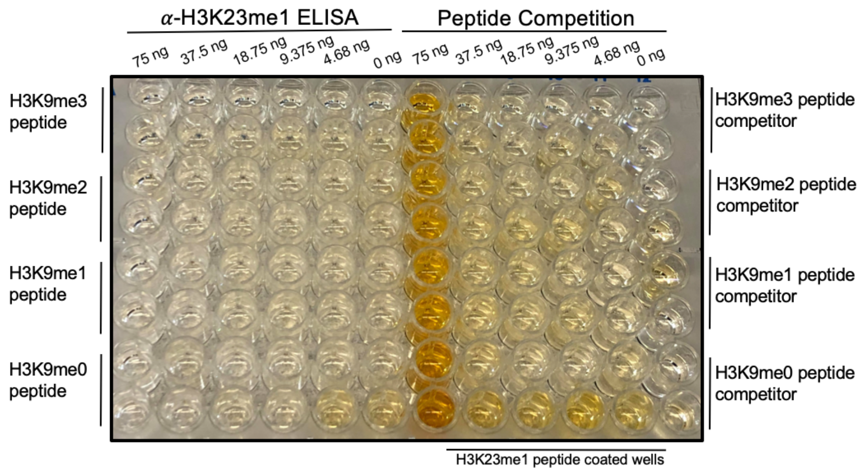

α-H3K23me1 ELISA
Peptide Competition
75 ng
37.5 ng
18.75 ng
9.375 ng
4.68 ng
0 ng
75 ng
37.5 ng
18.75 ng
9.375 ng
4.68 ng
0 ng
H3K9me3 peptide
H3K9me2 peptide
H3K9me1 peptide
H3K9me0 peptide
H3K9me3 peptide competitor
H3K9me2 peptide competitor
H3K9me1 peptide competitor
H3K9me0 peptide competitor
H3K23me1 peptide coated wells

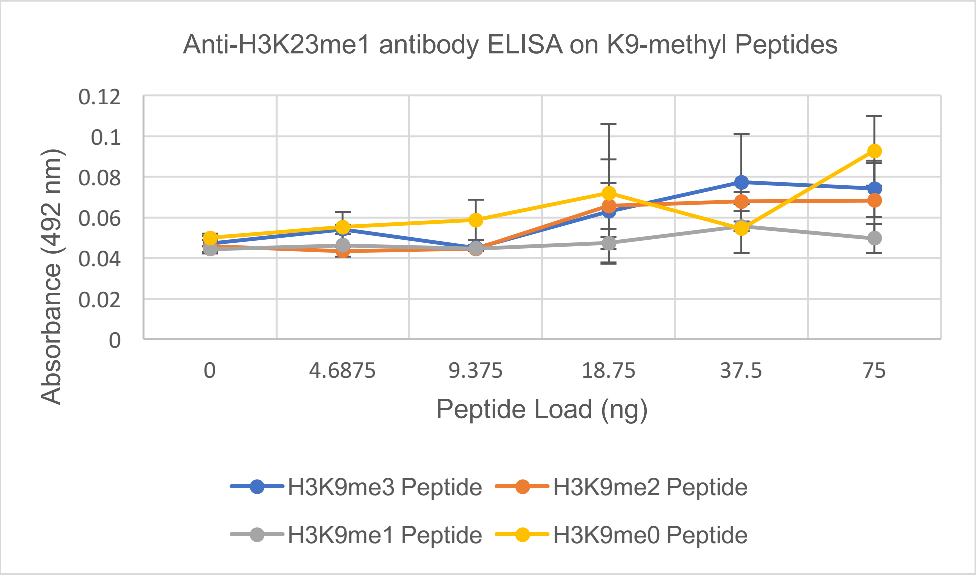

Anti-H3K23me1 antibody ELISA on K9-methyl Peptides
Absorbance (492 nm)
0.12
0.1
0.08
0.06
0.04
0.02
0
0
4.6875
9.375
18.75
37.5
75
Peptide Load (ng)
H3K9me3 Peptide
H3K9me2 Peptide
H3K9me1 Peptide
H3K9me0 Peptide

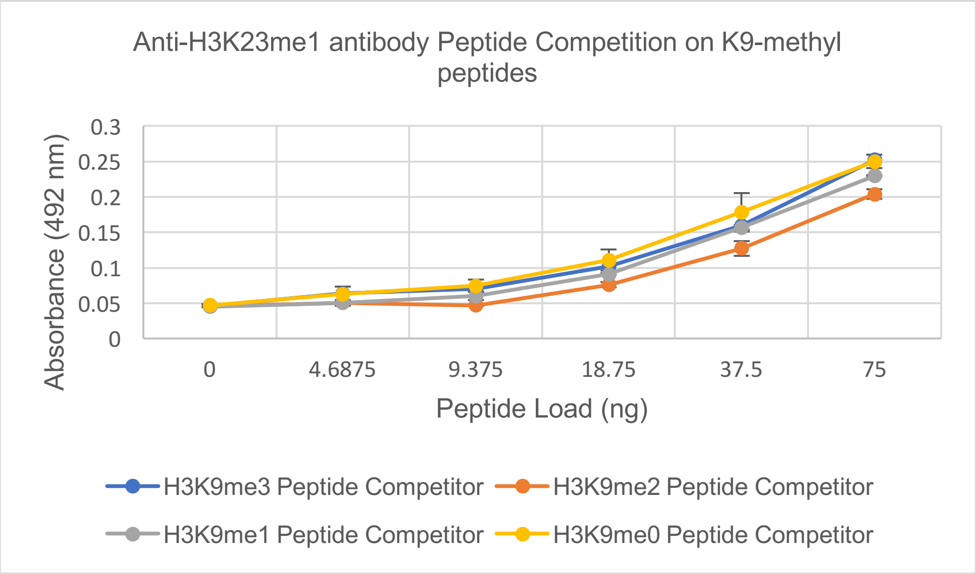

Anti-H3K23me1 antibody Peptide Competition on K9-methyl peptides
Absorbance (492 nm)
0.3
0.25
0.2
0.15
0.1
0.05
0
0
4.6875
9.375
18.75
37.5
75
Peptide Load (ng)
H3K9me3 Peptide Competitor
H3K9me2 Peptide Competitor
H3K9me1 Peptide Competitor
H3K9me0 Peptide Competitor

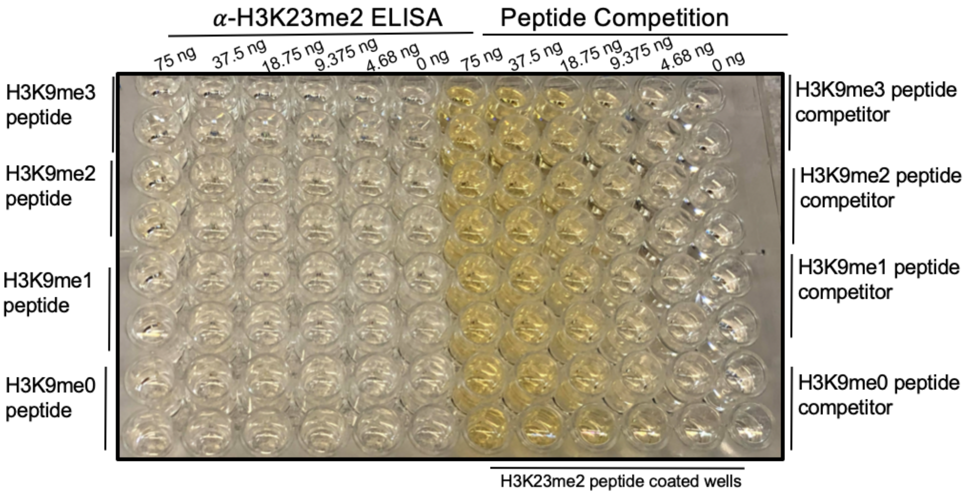

α-H3K23me2 ELISA
Peptide Competition
75 ng
37.5 ng
18.75 ng
9.375 ng
4.68 ng
0 ng
75 ng
37.5 ng
18.75 ng
9.375 ng
4.68 ng
0 ng
H3K9me3 peptide
H3K9me2 peptide
H3K9me1 peptide
H3K9me0 peptide
H3K9me3 peptide competitor
H3K9me2 peptide competitor
H3K9me1 peptide competitor
H3K9me0 peptide competitor
H3K23me2 peptide coated wells

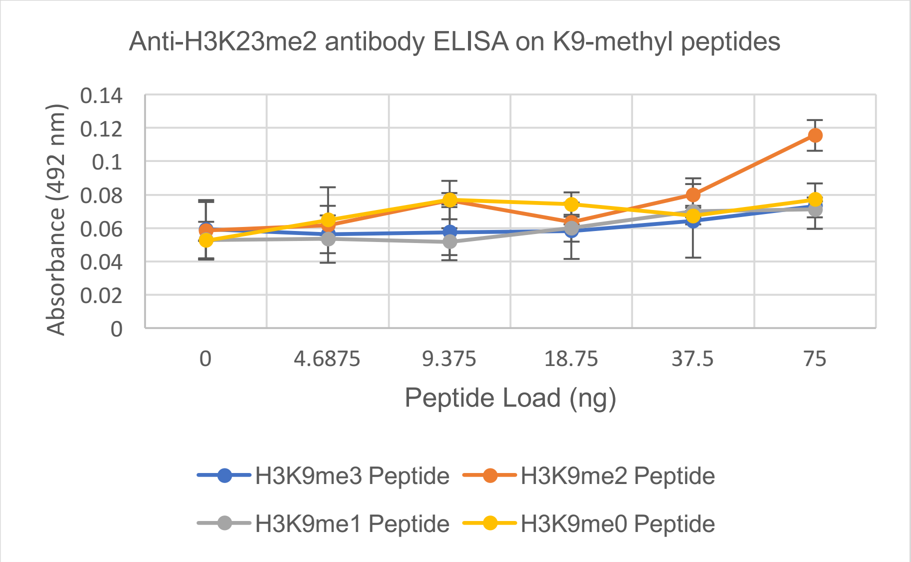

Anti-H3K23me2 antibody ELISA on K9-methyl peptides
Absorbance (492 nm)
0.14
0.12
0.1
0.08
0.06
0.04
0.02
0
0
4.6875
9.375
18.75
37.5
75
Peptide Load (ng)
H3K9me3 Peptide
H3K9me2 Peptide
H3K9me1 Peptide
H3K9me0 Peptide

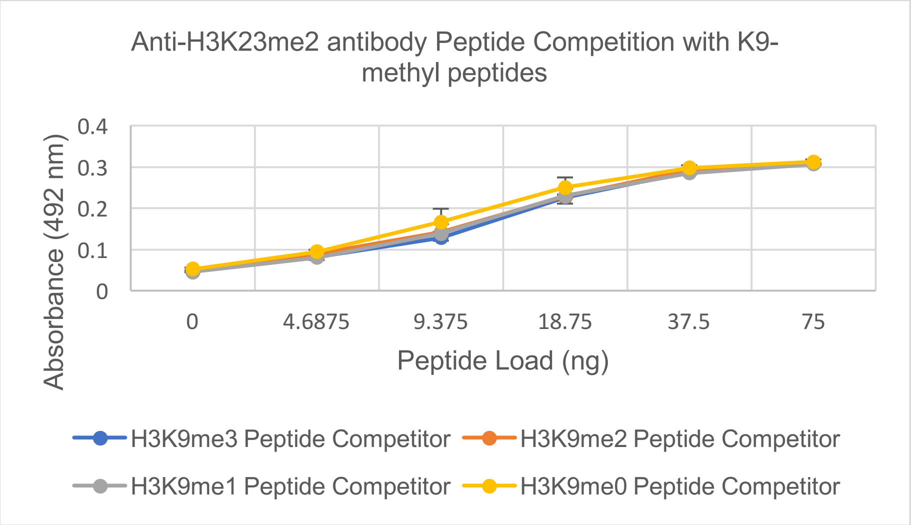

Anti-H3K23me2 antibody Peptide Competition with K9-methyl peptides
Absorbance (492 nm)
0.4
0.3
0.2
0.1
0
0
4.6875
9.375
18.75
37.5
75
Peptide Load (ng)
H3K9me3 Peptide Competitor
H3K9me2 Peptide Competitor
H3K9me1 Peptide Competitor
H3K9me0 Peptide Competitor

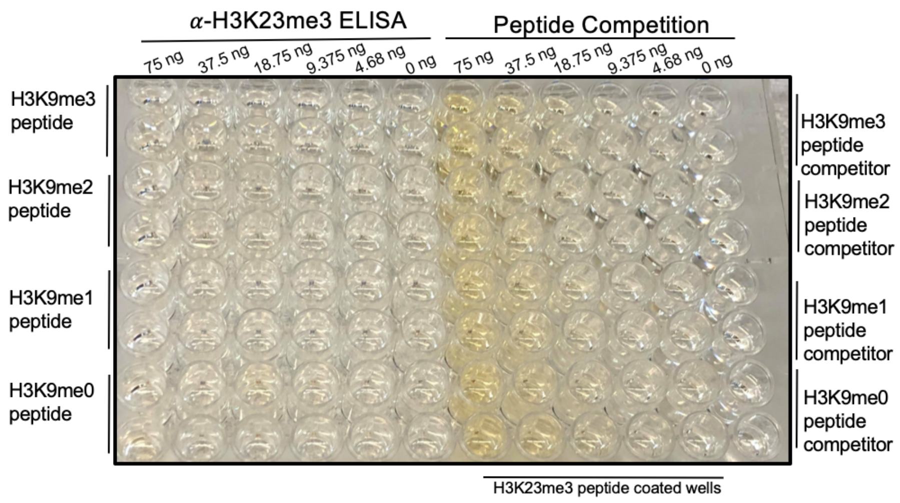
α-H3K23me3 ELISA
Peptide Competition
75 ng
37.5 ng
18.75 ng
9.375 ng
4.68 ng
0 ng
H3K9me3 peptide
H3K9me2 peptide
H3K9me1 peptide
H3K9me0 peptide
H3K9me3 peptide competitor
H3K9me2 peptide competitor
H3K9me1 peptide competitor
H3K9me0 peptide competitor
H3K23me3 peptide coated wells

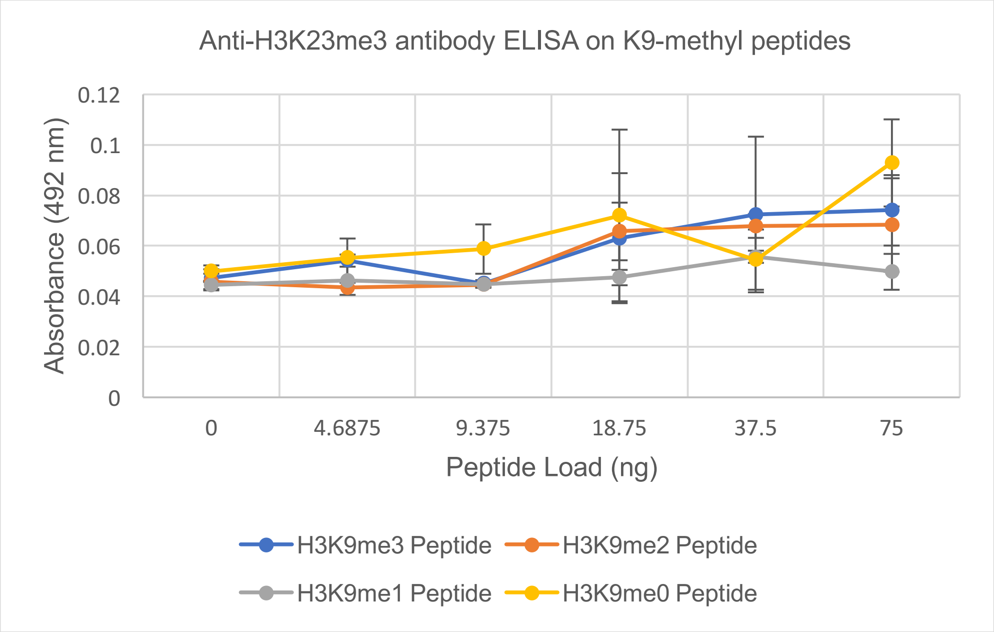
Anti-H3K23me3 antibody ELISA on K9-methyl peptides
Absorbance (492 nm)
0.12
0.1
0.08
0.06
0.04
0.02
0
0
4.6875
9.375
18.75
37.5
75
Peptide Load (ng)
H3K9me3 Peptide
H3K9me2 Peptide
H3K9me1 Peptide
H3K9me0 Peptide

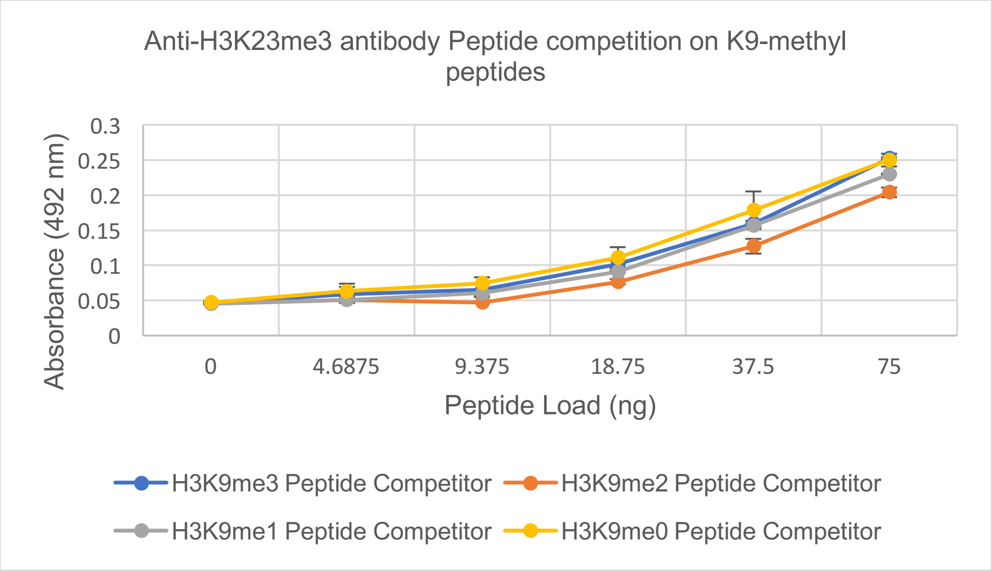
Anti-H3K23me3 antibody Peptide competition on K9-methyl peptides
Absorbance (492 nm)
0.3
0.25
0.2
0.15
0.1
0.05
0
0
4.6875
9.375
18.75
37.5
75
Peptide Load (ng)
H3K9me3 Peptide Competitor
H3K9me2 Peptide Competitor
H3K9me1 Peptide Competitor
H3K9me0 Peptide Competitor

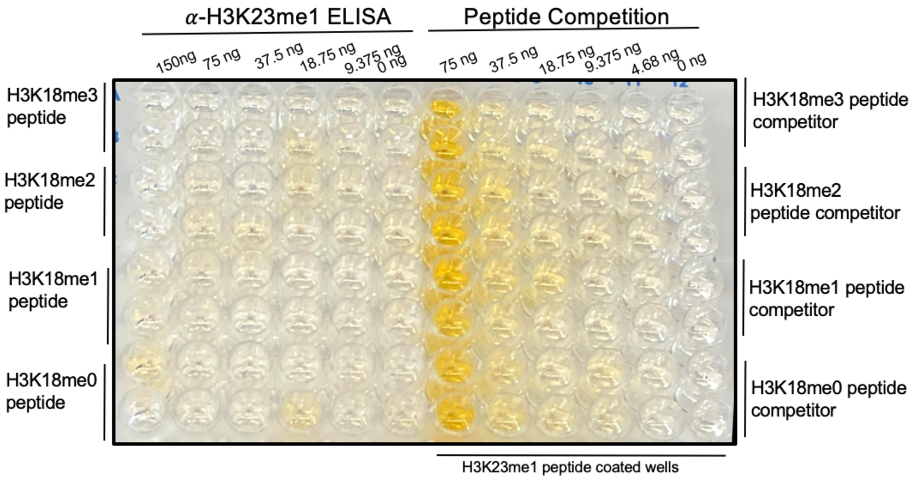

α-H3K23me1 ELISA
Peptide Competition
150ng
75 ng
37.5 ng
18.75 ng
9.375 ng
0 ng
75 ng
37.5 ng
18.75 ng
9.375 ng
4.68 ng
0 ng
H3K18me3 peptide
H3K18me2 peptide
H3K18me1 peptide
H3K18me0 peptide
H3K18me3 peptide competitor
H3K18me2 peptide competitor
H3K18me1 peptide competitor
H3K18me0 peptide competitor
H3K23me1 peptide coated wells

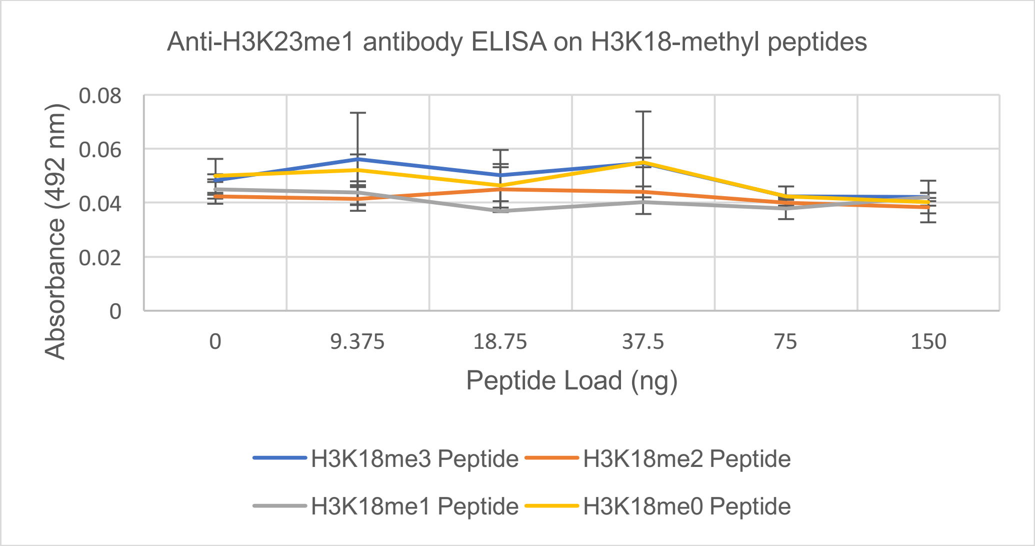

Anti-H3K23me1 antibody ELISA on H3K18-methyl peptides
Absorbance (492 nm)
0.08
0.06
0.04
0.02
0
0
9.375
18.75
37.5
75
150
Peptide Load (ng)
H3K18me3 Peptide
H3K18me2 Peptide
H3K18me1 Peptide
H3K18me0 Peptide

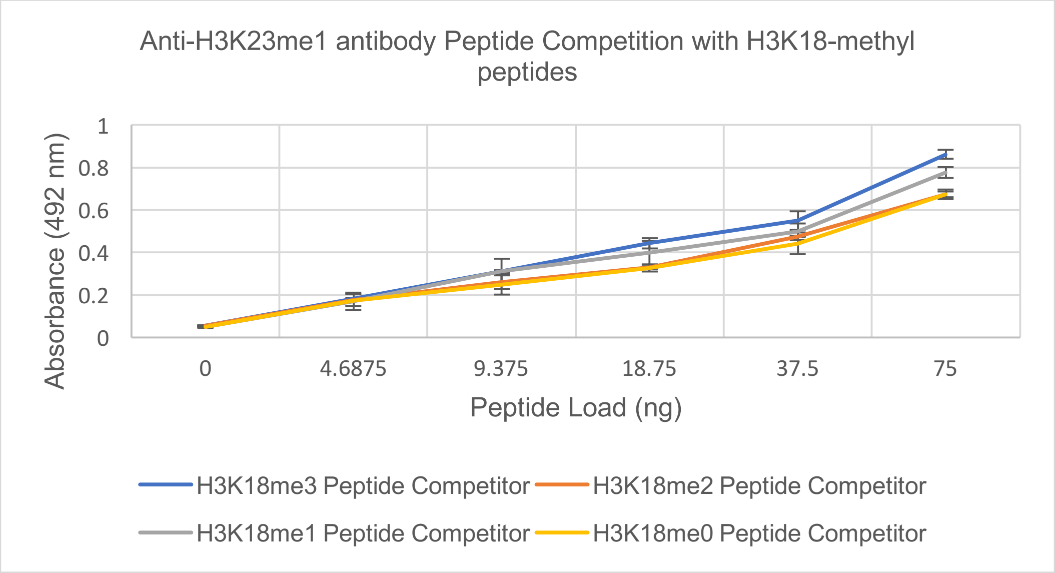

Anti-H3K23me1 antibody Peptide Competition with H3K18-methyl peptides
Absorbance (492 nm)
1
0.8
0.6
0.4
0.2
0
0
4.6875
9.375
18.75
37.5
75
Peptide Load (ng)
H3K18me3 Peptide Competitor
H3K18me2 Peptide Competitor
H3K18me1 Peptide Competitor
H3K18me0 Peptide Competitor

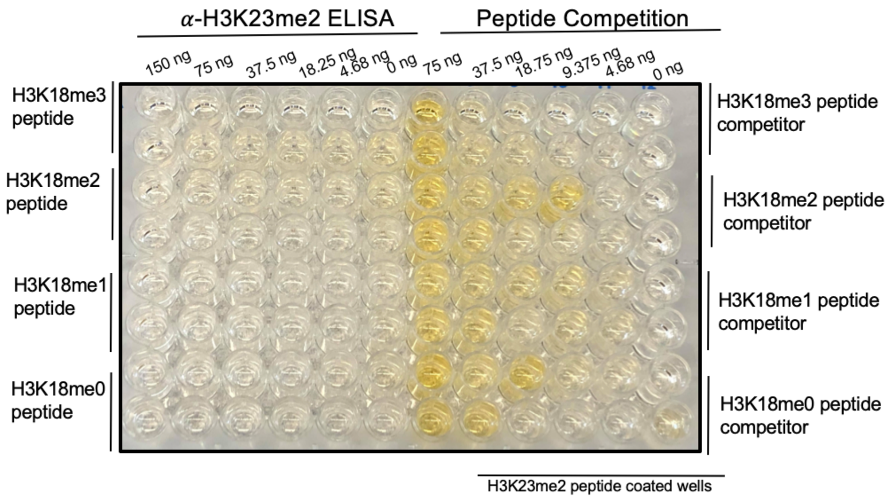
α-H3K23me2 ELISA
Peptide Competition
150 ng
75 ng
37.5 ng
18.25 ng
4.68 ng
0 ng
75 ng
37.5 ng
18.75 ng
9.375 ng
4.68 ng
0 ng
H3K18me3 peptide
H3K18me2 peptide
H3K18me1 peptide
H3K18me0 peptide
H3K18me3 peptide competitor
H3K18me2 peptide competitor
H3K18me1 peptide competitor
H3K18me0 peptide competitor
H3K23me2 peptide coated wells

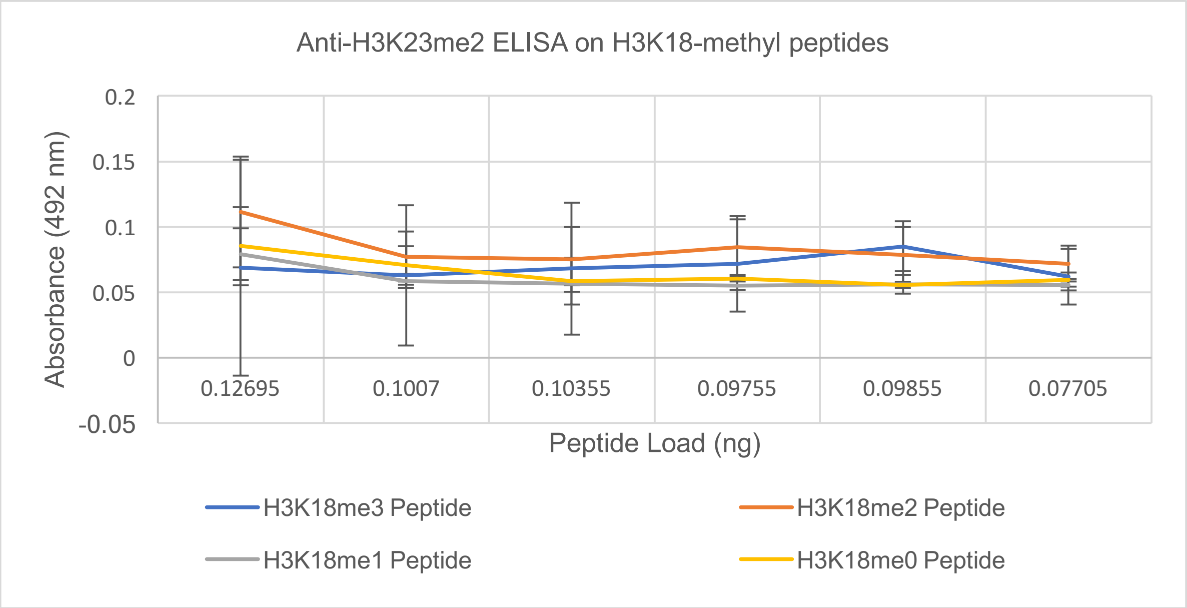
Anti-H3K23me2 ELISA on H3K18-methyl peptides
Absorbance (492 nm)
0.2
0.15
0.1
0.05
0
-0.05
0.12695
0.1007
0.10355
0.09755
0.09855
0.07705
Peptide Load (ng)
H3K18me3 Peptide
H3K18me2 Peptide
H3K18me1 Peptide
H3K18me0 Peptide

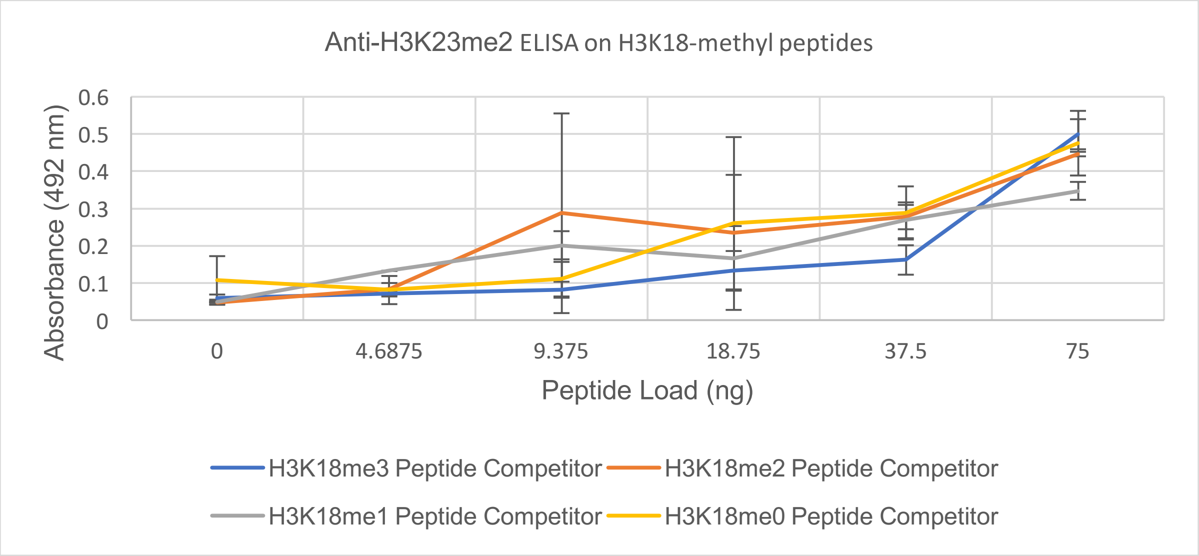
Anti-H3K23me2 ELISA on H3K18-methyl peptides
Absorbance (492 nm)
0.6
0.5
0.4
0.3
0.2
0.1
0
0
4.6875
9.375
18.75
37.5
75
Peptide Load (ng)
H3K18me3 Peptide Competitor
H3K18me2 Peptide Competitor
H3K18me1 Peptide Competitor
H3K18me0 Peptide Competitor

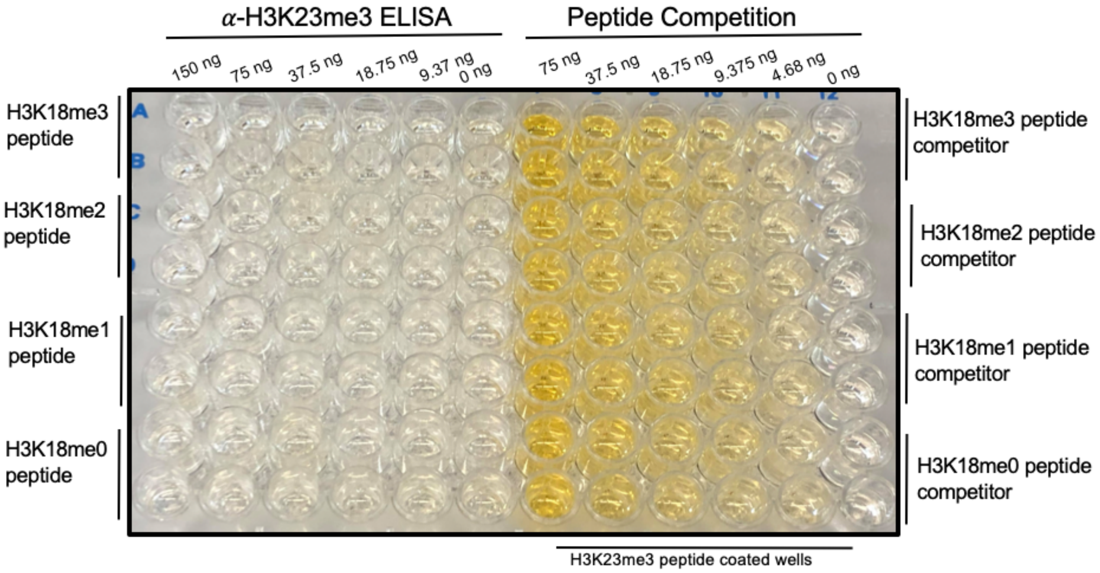
α-H3K23me3 ELISA
Peptide Competition
150 ng
75 ng
37.5 ng
18.75 ng
9.37 ng
0 ng
75 ng
37.5 ng
18.75 ng
9.375 ng
4.68 ng
0 ng
H3K18me3 peptide
H3K18me2 peptide
H3K18me1 peptide
H3K18me0 peptide
H3K18me3 peptide competitor
H3K18me2 peptide competitor
H3K18me1 peptide competitor
H3K18me0 peptide competitor
H3K23me3 peptide coated wells

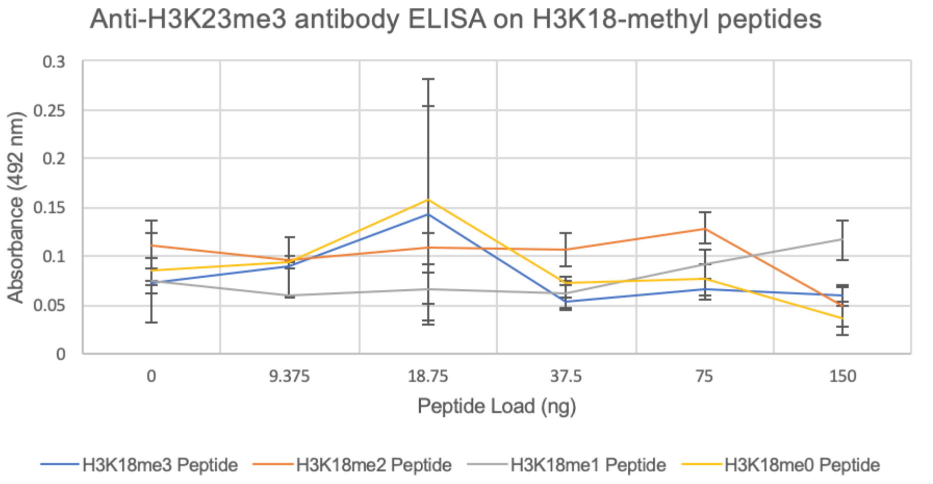
Anti-H3K23me3 antibody ELISA on H3K18-methyl peptides
Absorbance (492 nm)
0.3
0.25
0.2
0.15
0.1
0.05
0
0
9.375
18.75
37.5
75
150
Peptide Load (ng)
H3K18me3 Peptide
H3K18me2 Peptide
H3K18me1 Peptide
H3K18me0 Peptide

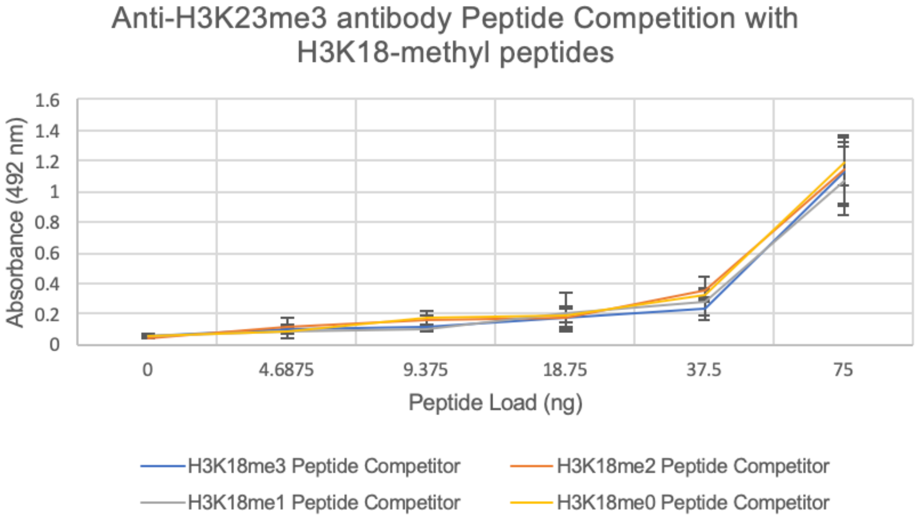
Anti-H3K23me3 antibody Peptide Competition with H3K18-methyl peptides
Absorbance (492 nm)
1.6
1.4
1.2
1
0.8
0.6
0.4
0.2
0
0
4.6875
9.375
18.75
37.5
75
Peptide Load (ng)
H3K18me3 Peptide Competitor
H3K18me2 Peptide Competitor
H3K18me1 Peptide Competitor
H3K18me0 Peptide Competitor

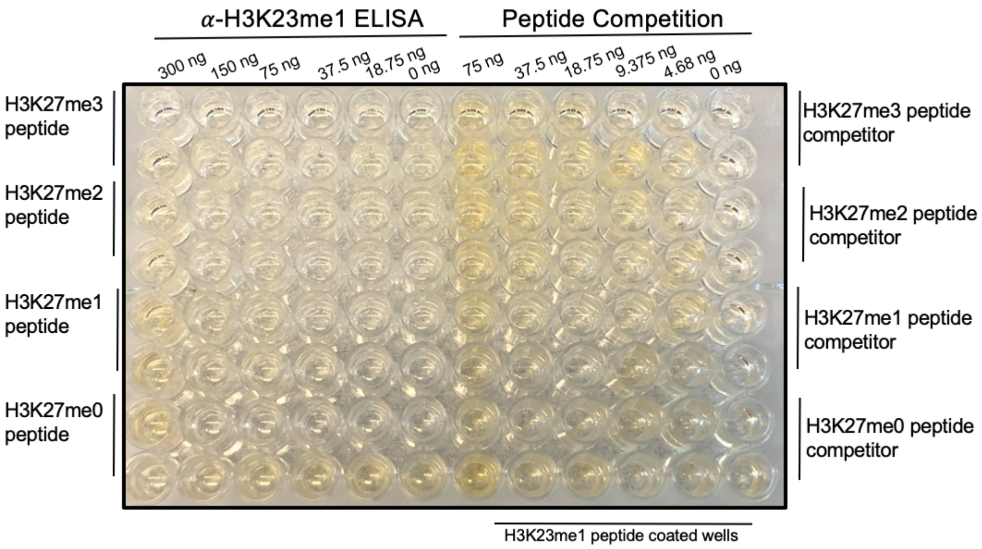
α-H3K23me1 ELISA
Peptide Competition
300 ng
150 ng
75 ng
37.5 ng
18.75 ng
0 ng
75 ng
37.5 ng
18.75 ng
9.375 ng
4.68 ng
0 ng
H3K27me3 peptide
H3K27me2 peptide
H3K27me1 peptide
H3K27me0 peptide
H3K27me3 peptide competitor
H3K27me2 peptide competitor
H3K27me1 peptide competitor
H3K27me0 peptide competitor
H3K23me1 peptide coated wells

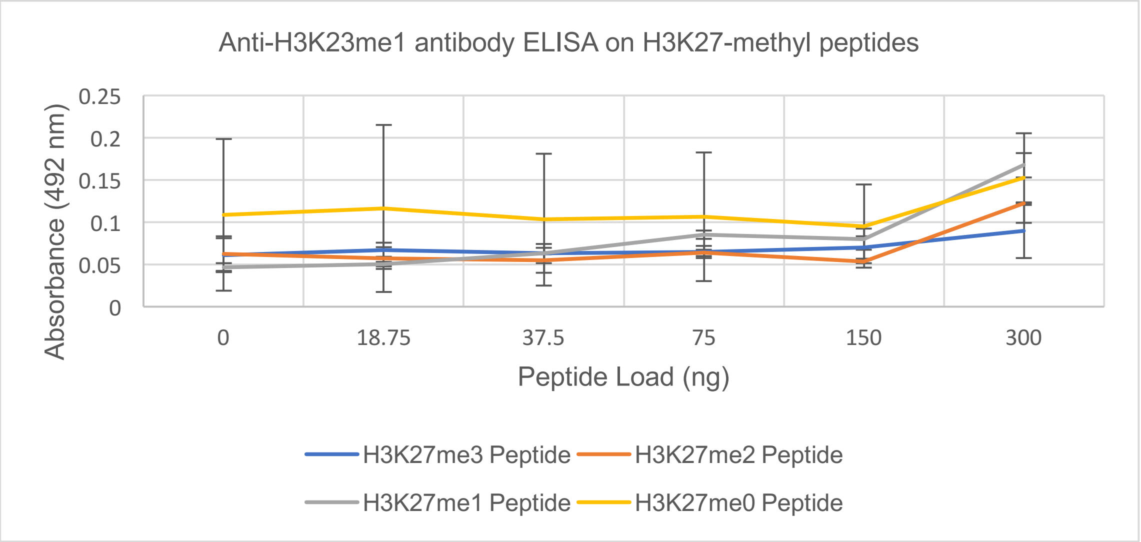
Anti-H3K23me1 antibody ELISA on H3K27-methyl peptides
Absorbance (492 nm)
0.25
0.2
0.15
0.1
0.05
0
0
18.75
37.5
75
150
300
Peptide Load (ng)
H3K27me3 Peptide
H3K27me2 Peptide
H3K27me1 Peptide
H3K27me0 Peptide

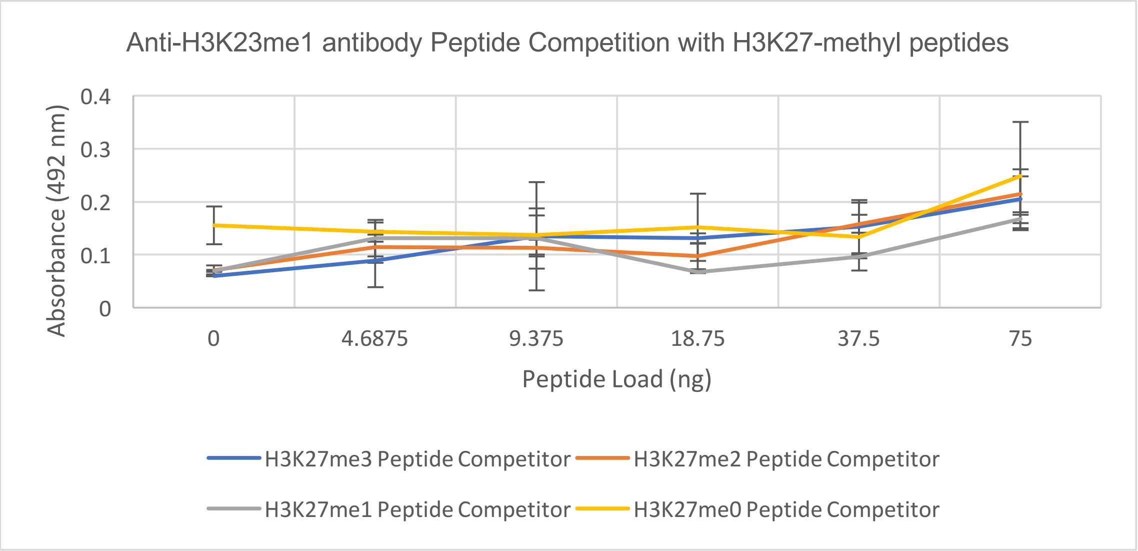
Anti-H3K23me1 antibody Peptide Competition with H3K27-methyl peptides
Absorbance (492 nm)
0.4
0.3
0.2
0.1
0
0
4.6875
9.375
18.75
37.5
75
Peptide Load (ng)
H3K27me3 Peptide Competitor
H3K27me2 Peptide Competitor
H3K27me1 Peptide Competitor
H3K27me0 Peptide Competitor

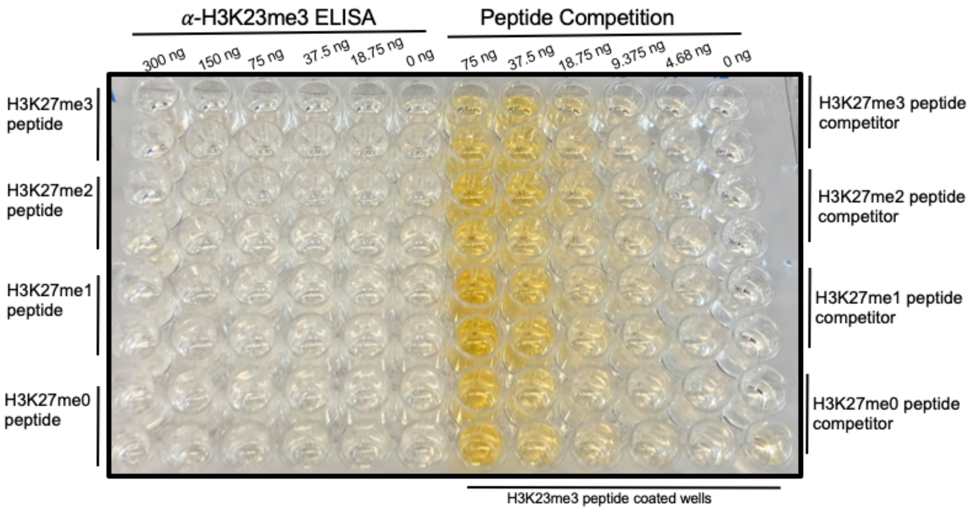
α-H3K23me3 ELISA
Peptide Competition
300 ng
150 ng
75 ng
37.5 ng
18.75 ng
0 ng
75 ng
37.5 ng
18.75 ng
9.375 ng
4.68 ng
0 ng
H3K27me3 peptide
H3K27me2 peptide
H3K27me1 peptide
H3K27me0 peptide
H3K27me3 peptide competitor
H3K27me2 peptide competitor
H3K27me1 peptide competitor
H3K27me0 peptide competitor
H3K23me3 peptide coated wells

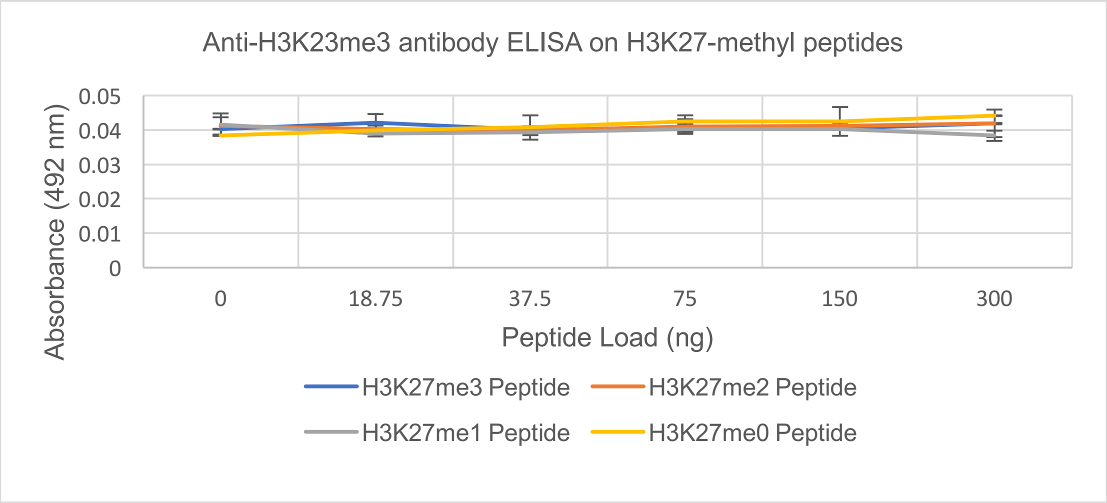
Anti-H3K23me3 antibody ELISA on H3K27-methyl peptides
Absorbance (492 nm)
0.05
0.04
0.03
0.02
0.01
0
0
18.75
37.5
75
150
300
Peptide Load (ng)
H3K27me3 Peptide
H3K27me2 Peptide
H3K27me1 Peptide
H3K27me0 Peptide

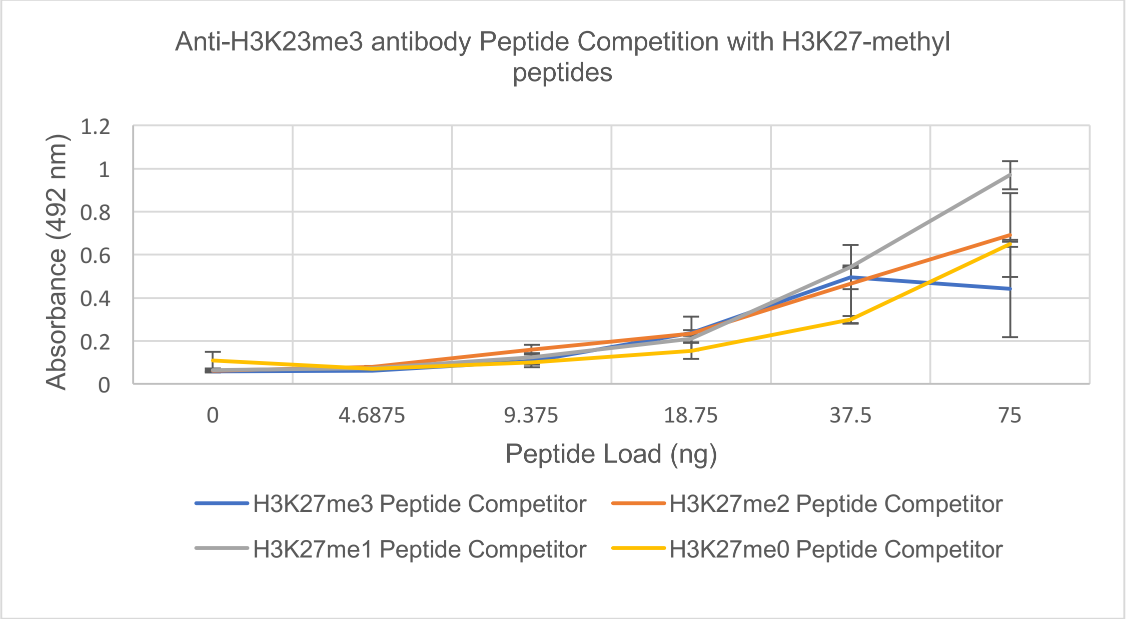
Anti-H3K23me3 antibody Peptide Competition with H3K27-methyl peptides
Absorbance (492 nm)
1.2
1
0.8
0.6
0.4
0.2
0
0
4.6875
9.375
18.75
37.5
75
Peptide Load (ng)
H3K27me3 Peptide Competitor
H3K27me2 Peptide Competitor
H3K27me1 Peptide Competitor
H3K27me0 Peptide Competitor

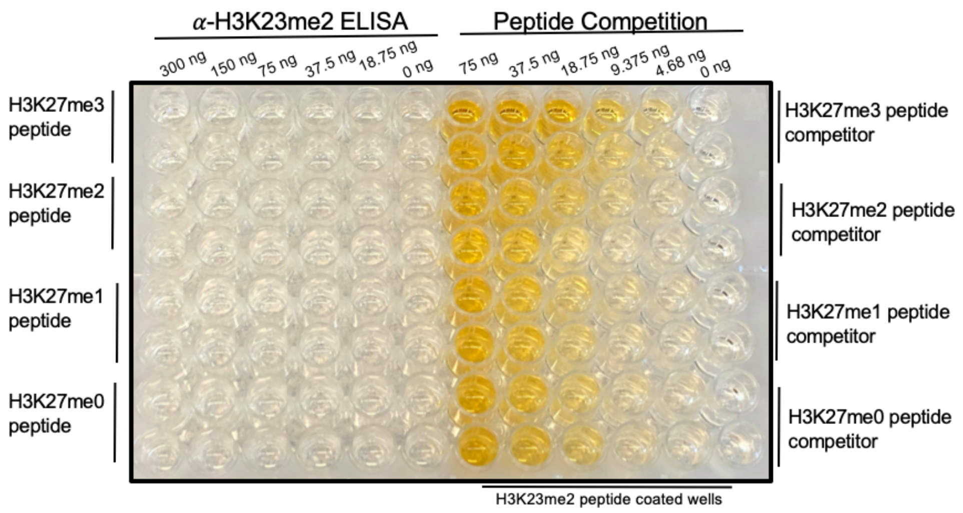

α-H3K23me2 ELISA
Peptide Competition
300 ng
150 ng
75 ng
37.5 ng
18.75 ng
0 ng
75 ng
37.5 ng
18.75 ng
9.375 ng
4.68 ng
0 ng
H3K27me3 peptide
H3K27me2 peptide
H3K27me1 peptide
H3K27me0 peptide
H3K27me3 peptide competitor
H3K27me2 peptide competitor
H3K27me1 peptide competitor
H3K27me0 peptide competitor
H3K23me2 peptide coated wells

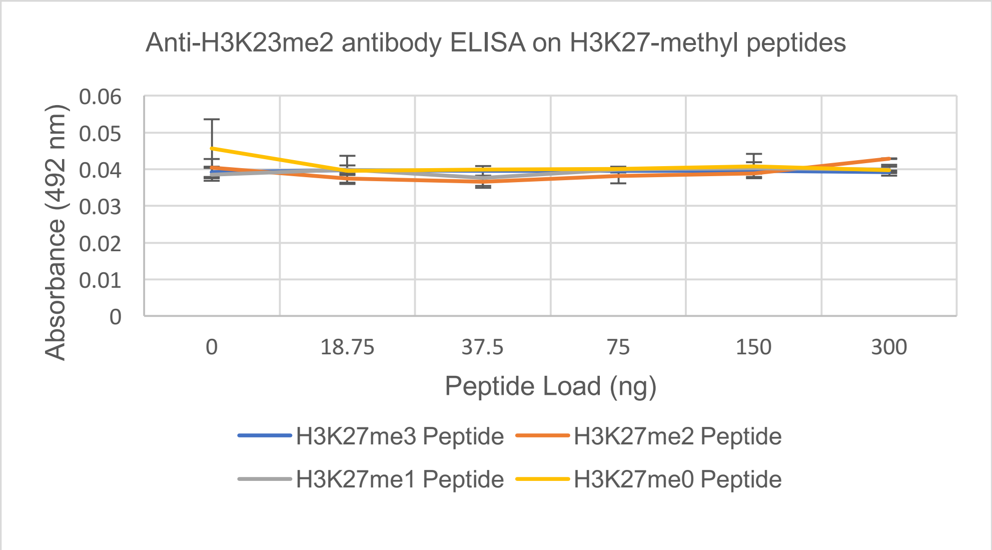

Anti-H3K23me2 antibody ELISA on H3K27-methyl peptides
Absorbance (492 nm)
0.06
0.05
0.04
0.03
0.02
0.01
0
0
18.75
37.5
75
150
300
Peptide Load (ng)
H3K27me3 Peptide
H3K27me2 Peptide
H3K27me1 Peptide
H3K27me0 Peptide

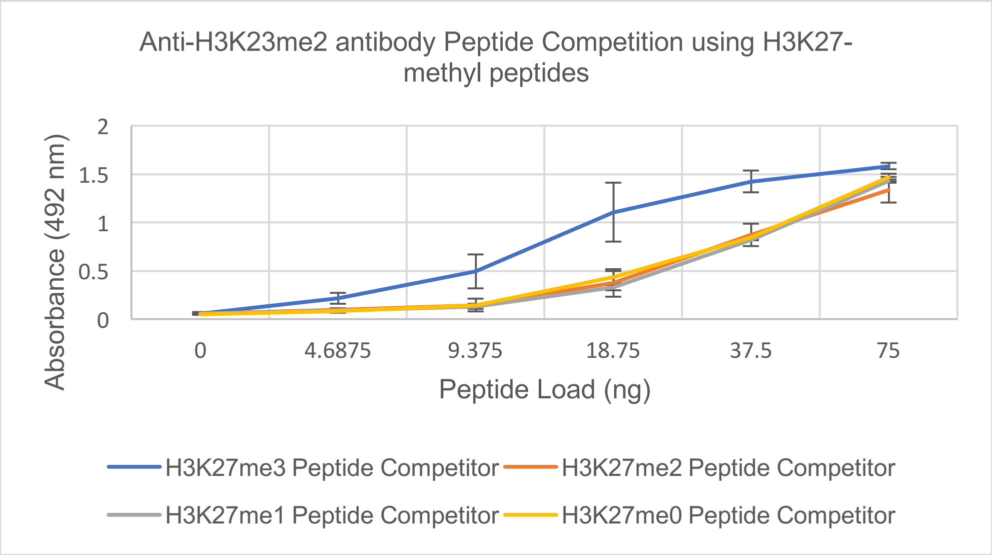

Anti-H3K23me2 antibody Peptide Competition using H3K27-methyl peptides
Absorbance (492 nm)
2
1.5
1
0.5
0
0
4.6875
9.375
18.75
37.5
75
Peptide Load (ng)
H3K27me3 Peptide Competitor
H3K27me2 Peptide Competitor
H3K27me1 Peptide Competitor
H3K27me0 Peptide Competitor

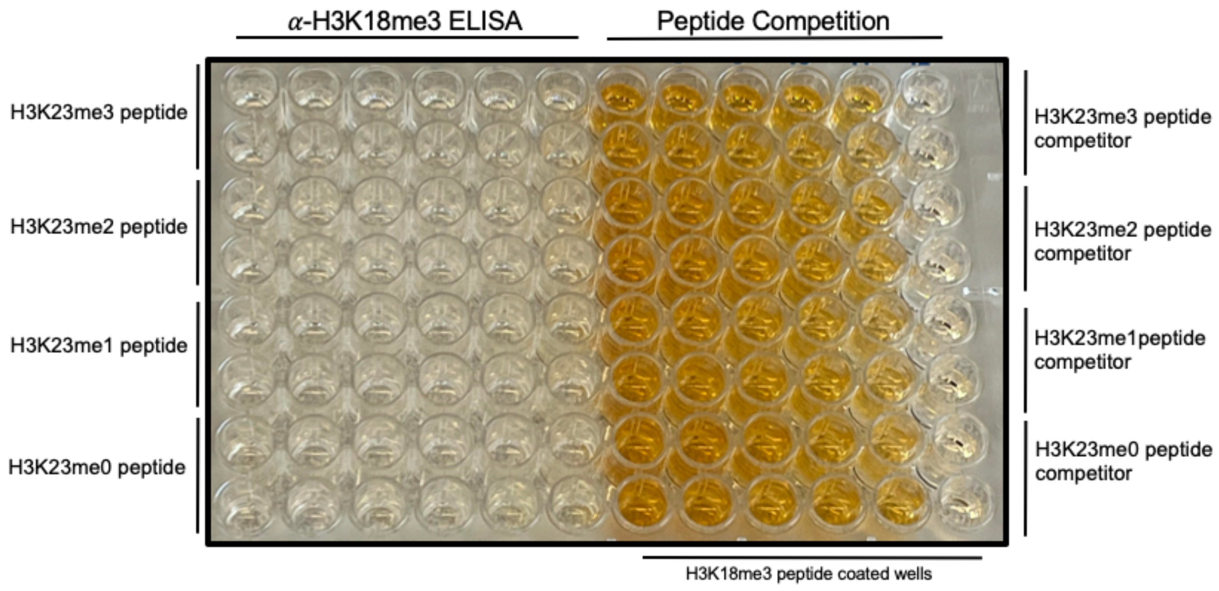
α-H3K18me3 ELISA
Peptide Competition
H3K23me3 peptide
H3K23me2 peptide
H3K23me1 peptide
H3K23me0 peptide
H3K23me3 peptide competitor
H3K23me2 peptide competitor
H3K23me1peptide competitor
H3K23me0 peptide competitor
H3K18me3 peptide coated wells

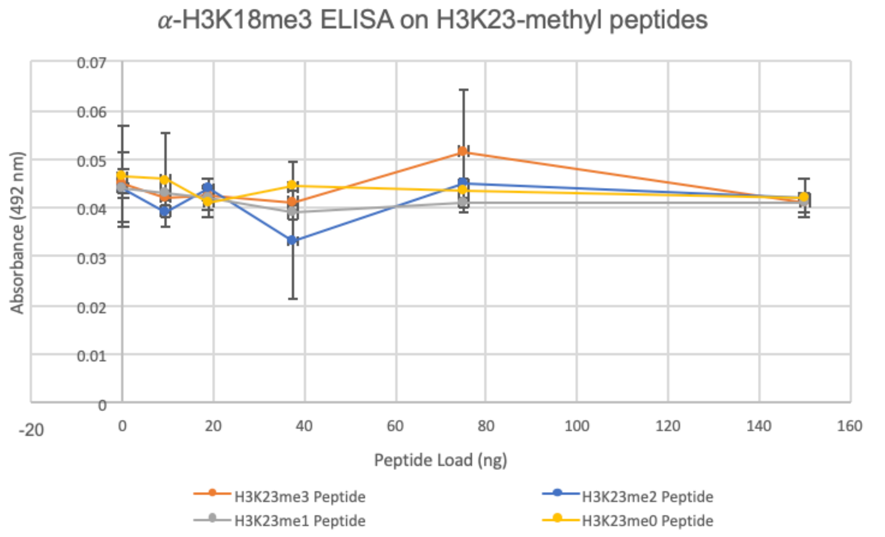
α-H3K18me3 ELISA on H3K23-methyl peptides
Absorbance (492 nm)
0.07
0.06
0.05
0.04
0.03
0.02
0.01
0
-20
0
20
40
60
80
100
120
140
160
Peptide Load (ng)
H3K23me3 Peptide
H3K23me2 Peptide
H3K23me1 Peptide
H3K23me0 Peptide

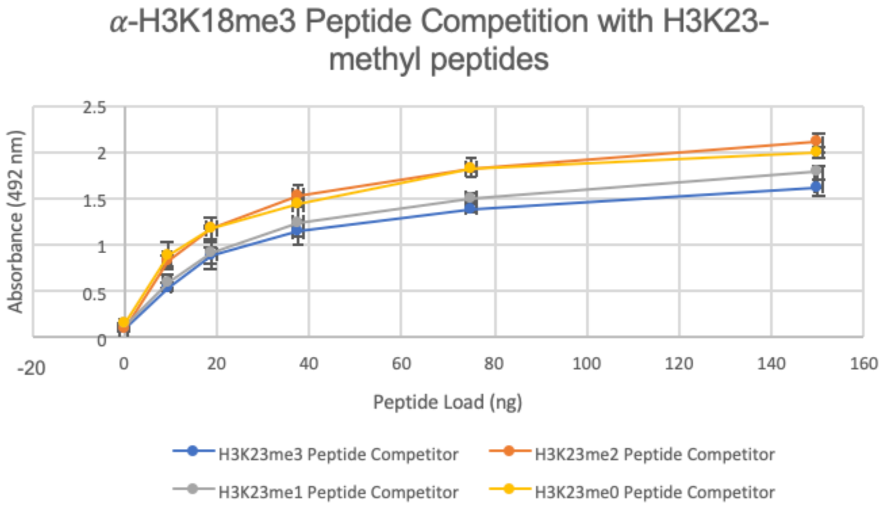
α-H3K18me3 Peptide Competition with H3K23-methyl peptides
Absorbance (492 nm)
2.5
2
1.5
1
0.5
0
-20
0
20
40
60
80
100
120
140
160
Peptide Load (ng)
H3K23me3 Peptide Competitor
H3K23me2 Peptide Competitor
H3K23me1 Peptide Competitor
H3K23me0 Peptide Competitor

Supplementary Figure 6. Antibody validation via ELISA and peptide competition assays

Using a 96-well plate ELISA format, ELISAs and peptide competition assays were done to validate H3K9me1, H3K9me2, H3K9me3, H3K18me1, H3K18me2, H3K18me3, H3K23me1, H3K23me2, H3K23me3, H3K27me1, H3K27me2 and H3K27me3 antibodies for their target methylation state, cross-reactivity with other methylation states of the same epitope and cross-reactivity with methyl-lysines of a different epitope (e.g. K9 antibodies with K23-methyl peptides, etc.)
